# Supplementary material for: New Nucleic Base-Tethered Trithiolato-Bridged Dinuclear Ruthenium(II)-Arene Compounds: Synthesis and Antiparasitic Activity
Source: Molecules. 2022 Nov 24;27(23):8173. doi: 10.3390/molecules27238173 (PMC9738179; doi:10.3390/molecules27238173)
Supplement: Supplementary file 1 [file molecules-27-08173-s001.zip › molecules-2052539-supplementary.pdf]

Supporting Information

New Nucleic Base-Tethered Trithiolato-Bridged Dinuclear Ruthenium(II)-Arene Compounds: Synthesis and Antiparasitic Activity

Oksana Desiatkina [1], Martin Mösching [1], Nicoleta Anghel [2], Ghalia Boubaker [2], Yosra Amdouni [2,3], Andrew Hemphill [2,*], Julien Furrer [1,*] and Emilia Păunescu [1,*]

[1] Department of Chemistry, Biochemistry and Pharmaceutical Sciences, University of Bern, Freiestrasse 3, 3012 Bern, Switzerland; oksankanahorna@gmail.com (O.D.); martin.moesching1@bluewin.ch (M.M.)

[2] Institute of Parasitology, Vetsuisse Faculty, University of Bern, Länggass-Strasse 122, 3012 Bern, Switzerland; nicoleta.anghel@vetsuisse.unibe.ch (N.A.); ghalia.boubaker@vetsuisse.unibe.ch (G.B.); amdouniyosra.ay@gmail.com (Y.A.)

[3] Laboratoire de Parasitologie, Institution de la Recherche et de l'Enseignement Supérieur Agricoles, Université de la Manouba, École Nationale de Médecine Vétérinaire de Sidi Thabet, Sidi Thabet 2020, Tunisia

* Correspondence: andrew.hemphill@vetsuisse.unibe.ch (A.H.); julien.furrer@unibe.ch (J.F.); paunescu_emilia@yahoo.com (E.P.); Tel.: +41-31-6842384 (A.H.); +41-31-6844383 (J.F.); Fax: +41-31-6312477 (A.H.)

Table of contents

Chemistry S2
1. General S2
2. Synthesis of the trithiolato-bridged dinuclear ruthenium(II)-arene intermediates **5-7** and **9**, **10** S2
3. Synthesis of the compounds **11-13** (family 1) S7
4. Synthesis of the compound **14** (family 2) S9
5. Synthesis of the compounds **15-26** (family 3) S10
6. Synthesis of compounds **27-33** (family 4) S19
7. Synthesis of compounds **34-39** (family 5) S24
8. Stability in DMSO-d_6 S30
Figure S1. ^{1}H NMR spectra of **4** and **24** recorded in DMSO-d_6 at 25°C; (A) recorded 5 min after sample preparation, and (B) sample after > 100 days storage at 0-5°C in the dark. S30
Figure S2. ^{1}H NMR spectra of **28**, **33** and **39** recorded in DMSO-d_6 at 25°C; (A) recorded 5 min after sample preparation, and (B) sample after > 100 days storage at 0-5°C in the dark. S31
Figure S3. Study of H-bond interactions with complementary nucleobases by ^{1}H NMR at r.t. for cytosine conjugate **25** and guanine. S32
Figure S4. Study of H-bond interactions with complementary nucleobases by ^{1}H NMR for uracil conjugate **23** and adenine. S33
Figure S5. Study of H-bond interactions with complementary nucleobases by ^{1}H NMR at r.t. for thymine conjugate **24** and adenine. S33
Figure S6. Study of H-bond interactions with complementary nucleobases by ^{1}H NMR for adenine conjugate **26** and uracil. S34
Figure S7. Study of H-bond interactions with complementary nucleobases by ^{1}H NMR for adenine conjugate **26** and thymine. S34

Chemistry

1. General

Chemicals were purchased from Aldrich, Alfa Aesar, Acros Organics, ABCR, and TCI Chemicals and used without further purification. Reactions were performed under inert atmosphere (N_2) using Schlenk techniques with dry solvents (Acros Organics) preserved over molecular sieves. ^1H (400.13 MHz) and ^{13}C (100.62 MHz) NMR spectra were recorded on a Bruker Avance II 400 spectrometer at 298 K. The chemical shifts are reported in parts per million (ppm) and referenced to residual solvent peaks [1] ($CDCl_3$, ^1H δ 7.26, $^{13}C\{^1H\}$ δ 77.16 ppm; MeOD-d_4, ^1H δ 3.31, $^{13}C\{^1H\}$ δ 49.00 ppm, DMSO-d_6, ^1H δ 2.50, $^{13}C\{^1H\}$ δ 39.52 ppm,), and coupling constants (J) are reported in hertz (Hz). High resolution electrospray ionization mass spectra (ESI-MS) were carried out by the Mass Spectrometry and Protein Analyses Services at DCBP and were obtained on a LTQ Orbitrap XL ESI (Thermo) operated in positive ion mode. Thermal elemental analyses were carried out by the Mass Spectrometry and Protein Analyses Services at DCBP and were obtained on a Flash 2000 Organic Elemental Analyzer (Thermo Scientific). Reactions were monitored by TLC using Merck TLC silica gel coated aluminium sheets 60 F254 and visualized with UV at 254 nm. Compounds were purified by column chromatography on silica gel using the elution systems indicated.

Abbreviations:

DIPEA - *N*,*N*-Diisopropylethylamine
DMAP - 4-(Dimethylamino)-pyridine
DMF - Dimethylformamide
EDCI - *N*-(3-Dimethylaminopropyl)-*N'*-ethylcarbodiimide hydrochloride
EtOAc - Ethyl acetate
Hex - *n*-Hexane
HOBt·H_2O - 1-Hydroxybenzotriazole hydrate
MsCl - Methanesulfonyl chloride
^{i}PrOH - 2-Propanol
TEA – Triethylamine

For the description of the NMR spectra: *Ar* – arene, *Tr* – triazole, *Py* – pyridine.

2. Synthesis of the trithiolato-bridged dinuclear ruthenium(II)-arene intermediates **5**-**7** and **9**, **10**

The dithiolato intermediate **1** ([(η^6-*p*-$MeC_6H_4Pr^i)_2Ru_2(\mu_2$-$SCH_2C_6H_4$-*p*-$Bu^t)Cl_2$]) was prepared and purified by adapting a previously described protocol[2,3]. Similarly, trithiolato diruthenium amino, carboxy and hydroxy derivatives **2** ([(η^6-*p*-$MeC_6H_4Pr^i)_2Ru_2(\mu_2$-$SCH_2C_6H_4$-*p*-$Bu^t)_2(\mu_2$-$SC_6H_4$-*p*-$NH_2$)]Cl), **3** ([($\eta^6$-*p*-$MeC_6H_4Pr^i)_2Ru_2(\mu_2$-$SCH_2C_6H_4$-*p*-$Bu^t)_2(\mu_2$-$SC_6H_4$-*p*-$CH_2CO_2H$)]Cl) and **4** ([($\eta^6$-*p*-$MeC_6H_4Pr^i)_2Ru_2(\mu_2$-$SCH_2C_6H_4$-*p*-$Bu^t)_2(\mu_2$-$SC_6H_4$-*o*-$CH_2OH$)]Cl) were synthesized following previously reported procedures [2,4].

Synthesis of [(η^6-*p*-$MeC_6H_4Pr^i)_2Ru_2(\mu_2$-$SCH_2C_6H_4$-*p*-$Bu^t)_2(\mu_2$-$SC_6H_4$-*p*-NH-R)]Cl (R = (C=O)-$(CH_2)_3$-C≡CH) (5)

Compound **5** was prepared and purified by adapting a literature protocol [2]. To a solution of 5-hexynoic acid (0.28 mL, 2.495 mmol, 1.3 equiv.) in dry CH_2Cl_2 (60 mL) at r.t. under inert atmosphere (N_2) was added EDCI (1.104 g, 5.757 mmol, 3 equiv.), and the resulting mixture was stirred for 10 min. Then, **2** (1.900 g, 1.919 mmol, 1 equiv.), HOBt·H_2O (0.778 g, 5.757 mmol, 3 equiv.) and DIPEA (1.67 mL,

9.959 mmol, 5 equiv.) were successively added and the resulting mixture was further stirred for 24 h. The reaction evolution was verified by TLC and the mixture was concentrated under reduced pressure. Purification by column chromatography (CH_2Cl_2/CH_3OH 10:1 (v/v)) afforded **5** as an orange solid (0.846 g, 0.781 mmol, yield 41%).

^{1}H-NMR ($CDCl_3$) δ_H, ppm: 7.57-7.63 (4H, m, 2xS-(*Ar*)C-C*H*-CH-C-NH-(C=O), 2xS-(*Ar*)C-CH-C*H*-C-NH-(C=O)), 7.33-7.42 (8H, m, 4xS-CH_2-(*Ar*)C-CH-C*H*-C-$C(CH_3)_3$, 4xS-CH_2-(*Ar*)C-C*H*-CH-C-$C(CH_3)_3$, $^3J_{H,H}$ = 7.1 Hz), 4.98 (2H, d, 2xCH_3-(*Ar*)C-CH-C*H*-C, $^3J_{H,H}$ = 5.4 Hz), 4.86 (2H, d, 2xCH_3-(*Ar*)C-CH-C*H*-C, $^3J_{H,H}$ = 5.5 Hz), 4.73 (2H, d, 2xCH_3-(*Ar*)C-C*H*-CH-C, $^3J_{H,H}$ = 5.4 Hz), 4.54 (2H, d, 2xCH_3-(*Ar*)C-C*H*-CH-C, $^3J_{H,H}$ = 5.5 Hz), 3.50 (2H, s, S-C$\underline{H_2}$-(*Ar*)C-CH-CH-C-$(CH_3)_3$), 3.31 (2H, s, S-C$\underline{H_2}$-(*Ar*)C-CH-CH-C-$(CH_3)_3$), 2.50 (2H, t, (C=O)-C$\underline{H_2}$-$(CH_2)_2$-C≡CH, $^3J_{H,H}$ = 7.3 Hz), 2.25 (2H, dt, (C=O)-$(CH_2)_2$-C$\underline{H_2}$-C≡CH, $^3J_{H,H}$ = 6.9 Hz, $^4J_{H,H}$ = 2.2 Hz), 2.00 (1H, t, (C=O)-$(CH_2)_3$-C≡C*H*, $^4J_{H,H}$ = 2.5 Hz), 1.84-1.94 (4H, m, 2x(*Ar*)C-CH-CH-C-C*H*$(CH_3)_2$, (C=O)-CH_2-C$\underline{H_2}$-CH_2-C≡CH), 1.63 (6H, s, 2xC$\underline{H_3}$-(*Ar*)C-CH-CH-C), 1.28 (9H, s, S-CH_2-(*Ar*)C-CH-CH-C-C(C$\underline{H_3}$)$_3$), 1.26 (9H, s, S-CH_2-(*Ar*)C-CH-CH-C-C(C$\underline{H_3}$)$_3$), 0.89 (6H, d, (*Ar*)C-CH-CH-C-CH(C$\underline{H_3}$)$_2$, $^3J_{H,H}$ = 6.8 Hz), 0.85 (6H, d, (*Ar*)C-CH-CH-C-CH(C$\underline{H_3}$)$_2$, $^3J_{H,H}$ = 6.8 Hz).

^{13}C-NMR ($CDCl_3$) δ_C, ppm: 172.2 (1C, (*C*=O)-$(CH_2)_2$-C≡CH), 151.9, 151.8 (2C, 2xS-CH_2-(*Ar*)C-CH-CH-*C*-$C(CH_3)_3$), 139.3 (1C, S-(*Ar*)C-CH-CH-*C*-NH), 136.6, 136.5 (2C, 2xS-CH_2-(*Ar*)*C*-CH-CH-C-$C(CH_3)_3$), 133.1 (2C, 2xS-(*Ar*)C-*C*H-CH-C-NH), 131.5 (1C, S-(*Ar*)*C*-CH-CH-C-NH), 129.2, 129.0 (4C, 4xS-CH_2-(*Ar*)C-*C*H-CH-C-$C(CH_3)_3$), 125.6, 125.4 (4C, 4xS-CH_2-(*Ar*)C-CH-*C*H-C-$C(CH_3)_3$), 119.9 (2C, 2xS-(*Ar*)C-CH-*C*H-C-NH), 107.3 (2C, 2xCH_3-(*Ar*)C-CH-CH-*C*), 100.3 (2C, 2xCH_3-(*Ar*)*C*-CH-CH-C), 83.9 (3C, 2xCH_3-(*Ar*)C-CH-*C*H-C, (C=O)-$(CH_2)_3$-*C*≡CH), 83.6 (2C, 2xCH_3-(*Ar*)C-*C*H-CH-C), 83.5 (2C, 2xCH_3-(*Ar*)C-CH-*C*H-C), 82.2 (2C, 2xCH_3-(*Ar*)C-*C*H-CH-C), 69.2 (1C, (C=O)-$(CH_2)_2$-C≡*C*H), 39.8 (1C, S-*C*H_2-(*Ar*)C-CH-CH-C-$C(CH_3)_3$), 39.3 (1C, S-*C*H_2-(*Ar*)C-CH-CH-C-$C(CH_3)_3$), 35.8 (1C, (C=O)-*C*H_2-$(CH_2)_2$-C≡CH), 34.73, 34.70 (2C, 2xS-CH_2-(*Ar*)C-CH-CH-C-*C*$(CH_3)_3$), 31.32, 31.30 (6C, 2xS-CH_2-(*Ar*)C-CH-CH-C-C(*C*H_3)$_3$), 30.9 (2C, 2x(*Ar*)CH-CH-C-*C*H$(CH_3)_2$), 24.2 (1C, (C=O)-CH_2-*C*H_2-CH_2-C≡CH), 22.9 (2C, (*Ar*)CH-CH-C-CH(*C*H_3)$_2$), 22.7 (2C, (*Ar*)CH-CH-C-CH(*C*H_3)$_2$), 18.0 (3C, 2x*C*H_3-(*Ar*)C-CH-CH, (C=O)-$(CH_2)_2$-*C*H_2-C≡CH).

R_f (CH_2Cl_2/CH_3OH 10:1 (v/v)) = 0.468.

ESI-MS(+): *m/z* found 1048.2739 $[M-Cl]^+$, calcd. for $C_{54}H_{70}NORu_2S_3^+$ 1048.2701.

Elemental analysis (%): calcd. for $C_{54}H_{70}ClNORu_2S_3\cdot CH_2Cl_2$ C 58.77, H 641, N 1.26; found C 58.79, H 6.42, N 1.32.

Synthesis of [(η^6-*p*-MeC$_6$H$_4$Pr^i)$_2$Ru$_2$(μ_2-SCH$_2$C$_6$H$_4$-*p*-Bu^t)$_2$(μ_2-SC$_6$H$_4$-*p*-NH-R)]Cl (R = (C=O)-C$_6$H$_4$-*p*-C≡CH) (6)

Compound **6** was prepared and purified by adapting a literature procedure [2]. To a solution of 4-ethynylbenzoic acid (0.084 g, 0.574 mmol, 1.3 equiv.) in dry CH_2Cl_2 (60 mL) at r.t. under inert atmosphere (N_2) was added EDCI (0.254 g, 1.329 mmol, 3 equiv.), and the resulting mixture was stirred for 10 min. Then **2** (0.440 g, 0.444 mmol, 1 equiv.), HOBt·H_2O (0.204 g, 1.333 mmol, 3 equiv.) and DIPEA (0.38 mL, 2.170 mmol, 5 equiv.) were successively added and the resulting mixture was further stirred for 24 h. The reaction evolution was verified by TLC and the mixture was concentrated under reduced pressure. Purification by column chromatography (CH_2Cl_2/CH_3OH 10:1 (v/v)) afforded **6** as an orange solid (0.170 g, 0.153 mmol, yield 34%).

^{1}H-NMR ($CDCl_3$) δ_H, ppm: 11.27 (1H, NH-(C=O)), 8.52 (2H, d, 2xS-(*Ar*)C-CH-C*H*-C-NH-(C=O), $^3J_{H,H}$ = 8.4 Hz), 8.50 (2H, d, 2x(C=O)-(*Ar*)C-C*H*-CH-C, $^3J_{H,H}$ = 8.6 Hz), 7.64 (2H, d, 2x(C=O)-(*Ar*)C-CH-C*H*-C, $^3J_{H,H}$ = 8.4 Hz), 7.60 (2H, d, 2xS-(*Ar*)C-C*H*-CH-C-NH-(C=O), $^3J_{H,H}$ = 8.2 Hz), 7.40-7.50 (8H, m, 4xS-CH_2-(*Ar*)C-CH-C*H*-C-$C(CH_3)_3$, 4xS-CH_2-(*Ar*)C-C*H*-CH-C-$C(CH_3)_3$, $^3J_{H,H}$ = 8.3 Hz), 5.00

(2H, d, 2xCH$_3$-(*Ar*)C-CH-C$\underline{H}$-C, $^3J_{H,H}$ = 5.7 Hz), 4.89 (2H, d, 2xCH$_3$-(*Ar*)C-CH-C$\underline{H}$-C, $^3J_{H,H}$ = 5.8 Hz), 4.73 (2H, d, 2xCH$_3$-(*Ar*)C-C$\underline{H}$-CH-C, $^3J_{H,H}$ = 5.7 Hz), 4.62 (2H, d, 2xCH$_3$-(*Ar*)C-C$\underline{H}$-CH-C, $^3J_{H,H}$ = 5.8 Hz), 3.56 (2H, s, S-C$\underline{H_2}$-(*Ar*)C-CH-CH-C-(CH$_3$)$_3$, 3.36 (2H, s, S-C$\underline{H_2}$-(*Ar*)C-CH-CH-C-(CH$_3$)$_3$, 3.14 (1H, s, (C=O)-(*Ar*)C-CH-CH-C-C≡C$\underline{H}$), 2.02 (2H, sept, 2x(*Ar*)C-CH-CH-C-C$\underline{H}$(CH$_3$)$_2$, $^3J_{H,H}$ = 6.8 Hz), 1.69 (6H, s, 2xC$\underline{H_3}$-(*Ar*)C-CH-CH-C), 1.37 (9H, s, S-CH$_2$-(*Ar*)C-CH-CH-C-C(C$\underline{H_3}$)$_3$), 1.35 (9H, s, S-CH$_2$-(*Ar*)C-CH-CH-C-C(C$\underline{H_3}$)$_3$), 0.99 (6H, d, (*Ar*)C-CH-CH-C-CH(C$\underline{H_3}$)$_2$, $^3J_{H,H}$ = 6.8 Hz), 0.95 (6H, d, (*Ar*)C-CH-CH-C-CH(C$\underline{H_3}$)$_2$, $^3J_{H,H}$ = 6.8 Hz).

^{13}C-NMR (CDCl$_3$) δ_C, ppm: 166.4 (($\underline{C}$=O)-(*Ar*)C-CH-CH-C), 152.01, 151.99 (2C, 2xS-CH$_2$-(*Ar*)C-CH-CH-$\underline{C}$-C(CH$_3$)$_3$), 141.6 (1C, (C=O)-(*Ar*)$\underline{C}$-CH-CH-C-C≡CH), 136.9, 136.5 (2C, 2xS-CH$_2$-(*Ar*)$\underline{C}$-CH-CH-C-C(CH$_3$)$_3$), 134.7 (1C, S-(*Ar*)C-CH-CH-$\underline{C}$-NH-(C=O)), 132.6 (2C, 2x(C=O)-(*Ar*)C-CH-$\underline{C}$H-C-C≡CH), 132.1 (2C, 2xS-(*Ar*)C-$\underline{C}$H-CH-C-NH-(C=O)), 130.1 (1C, (C=O)-(*Ar*)C-CH-CH-$\underline{C}$-C≡CH), 129.3, 129.1 (4C, 4xS-CH$_2$-(*Ar*)C-$\underline{C}$H-CH-C-C(CH$_3$)$_3$), 129.0 (2C, 2xS-(*Ar*)C-CH-$\underline{C}$H-C-NH-(C=O)), 125.72, 125.67 (4C, 4xS-CH$_2$-(*Ar*)C-CH-$\underline{C}$H-C-C(CH$_3$)$_3$), 125.1 (1C, S-(*Ar*)$\underline{C}$-CH-CH-C-NH-(C=O)), 121.9 (2C, 2x(C=O)-(*Ar*)C-$\underline{C}$H-CH-C-C≡CH), 107.7 (2C, 2xCH$_3$-(*Ar*)C-CH-CH-$\underline{C}$), 100.3 (2C, 2xCH$_3$-(*Ar*)$\underline{C}$-CH-CH-C), 84.3 (2C, 2xCH$_3$-(*Ar*)C-CH-$\underline{C}$H-C), 83.8 (2C, 2xCH$_3$-(*Ar*)C-$\underline{C}$H-CH-C), 83.3 (2C, 2xCH$_3$-(*Ar*)C-CH-$\underline{C}$H-C), 82.3 (2C, 2xCH$_3$-(*Ar*)C-$\underline{C}$H-CH-C), 78.8 (2C, (C=O)-(*Ar*)C-CH-CH-C-$\underline{C}$≡CH, (C=O)-(*Ar*)C-CH-CH-C-C≡$\underline{C}$H), 39.9 (1C, S-$\underline{C}$H$_2$-(*Ar*)C-CH-CH-C-C(CH$_3$)$_3$), 39.3 (1C, S-$\underline{C}$H$_2$-(*Ar*)C-CH-CH-C-C(CH$_3$)$_3$), 35.0, 34.9 (2C, 2xS-CH$_2$-(*Ar*)C-CH-CH-C-$\underline{C}$(CH$_3$)$_3$), 31.6, 31.5 (6C, 2xS-CH$_2$-(*Ar*)C-CH-CH-C-C($\underline{C}$H$_3$)$_3$), 31.2 (2C, 2x(*Ar*)CH-CH-C-$\underline{C}$H(CH$_3$)$_2$), 23.2 (2C, (*Ar*)CH-CH-C-CH($\underline{C}$H$_3$)$_2$), 23.0 (2C, (*Ar*)CH-CH-C-CH($\underline{C}$H$_3$)$_2$), 18.2 (2C, 2x$\underline{C}$H$_3$-(*Ar*)C-CH-CH).

R_f (CH$_2$Cl$_2$/CH$_3$OH 10:1) = 0.212.

ESI-MS(+): *m/z* found 1082.2569 [M-Cl]$^+$, calcd. for $C_{57}H_{68}NORu_2S_3^+$ 1082.2545.

Elemental analysis (%): calcd. for $C_{57}H_{68}NORu_2S_3Cl\cdot1.5CH_3OH$ C 60.31, H 6.40, N 1.20; found C 60.32, H 6.60, N 0.91.

Synthesis of [(η^6-*p*-MeC$_6$H$_4$Pr^i)$_2$Ru$_2$(μ_2-SCH$_2$C$_6$H$_4$-*p*-Bu^t)$_2$(μ_2-SC$_6$H$_4$-*p*-CH$_2$-(C=O)-O-R)]Cl (R = CH$_2$-C≡CH) (7)

Compound **7** was prepared and purified by adapting a literature procedure [2]. To a solution of **3** (1.000 g, 0.968 mmol, 1.0 equiv.) in dry CH$_2$Cl$_2$ (60 mL) at r.t. under inert atmosphere (N$_2$) was added EDCI (0.276 g, 1.445 mmol, 1.5 equiv.), and the resulting mixture was stirred for 10 min. Then, propargyl alcohol (0.08 mL, 1.444 mmol, 1.5 equiv.) and DMAP (0.035 g, 0.286 mmol, 0.3 equiv.) were successively added and the resulting mixture was further stirred for 24 h. The reaction evolution was verified by TLC and the mixture was concentrated under reduced pressure. Purification by column chromatography (CH$_2$Cl$_2$/CH$_3$OH 10:1 (v/v)) afforded **7** as an orange solid (0.618 g, 0.561 mmol, yield 60%).

^{1}H-NMR (CDCl$_3$) δ_H, ppm: 7.72 (2H, d, S-(*Ar*)C-CH-C$\underline{H}$-C-CH$_2$-(C=O)-O, $^3J_{H,H}$ = 7.9 Hz), 7.38-7.48 (8H, m, 4xS-CH$_2$-(*Ar*)C-C$\underline{H}$-CH-C-C(CH$_3$)$_3$, 4xS-CH$_2$-(*Ar*)C-CH-C$\underline{H}$-C-C(CH$_3$)$_3$, $^3J_{H,H}$ = 8.6 Hz), 7.23 (2H, d, S-(*Ar*)C-C$\underline{H}$-CH-C-CH$_2$-(C=O)-O, $^3J_{H,H}$ = 7.9 Hz), 5.11 (2H, d, 2xCH$_3$-(*Ar*)C-CH-C$\underline{H}$-C, $^3J_{H,H}$ = 5.5 Hz), 5.00 (2H, d, 2xCH$_3$-(*Ar*)C-CH-C$\underline{H}$-C, $^3J_{H,H}$ = 5.7 Hz), 4.91 (2H, d, 2xCH$_3$-(*Ar*)C-C$\underline{H}$-CH-C, $^3J_{H,H}$ = 5.5 Hz), 4.70 (2H, d, (C=O)-O-C$\underline{H_2}$-C≡CH, $^4J_{H,H}$ = 2.5 Hz), 4.61 (2H, d, 2xCH$_3$-(*Ar*)C-C$\underline{H}$-CH-C, $^3J_{H,H}$ = 5.7 Hz), 3.67 (2H, s, S-(*Ar*)C-CH-CH-C-C$\underline{H_2}$-(C=O)-O), 3.60 (2H, s, S-C$\underline{H_2}$-(*Ar*)C-CH-CH-C-(CH$_3$)$_3$, 3.42 (2H, s, S-C$\underline{H_2}$-(*Ar*)C-CH-CH-C-(CH$_3$)$_3$, 2.49 (1H, t, (C=O)-O-CH$_2$-C≡C$\underline{H}$, $^4J_{H,H}$ = 2.4 Hz), 1.88 (2H, sept, 2x(*Ar*)C-CH-CH-C-C$\underline{H}$(CH$_3$)$_2$, $^3J_{H,H}$ = 6.8 Hz), 1.74 (6H, s, 2xC$\underline{H_3}$-(*Ar*)C-CH-CH-C), 1.35 (9H, s, S-CH$_2$-(*Ar*)C-CH-CH-C-C(C$\underline{H_3}$)$_3$), 1.32 (9H, s, S-CH$_2$-(*Ar*)C-CH-CH-C-C(C$\underline{H_3}$)$_3$), 0.92 (6H, d, 2x(*Ar*)C-CH-CH-C-CH(C$\underline{H_3}$)$_2$, $^3J_{H,H}$ = 6.8 Hz), 0.88 (6H, d, 2x(*Ar*)C-CH-CH-C-CH(C$\underline{H_3}$)$_2$, $^3J_{H,H}$ = 6.8 Hz).

^{13}C-NMR ($CDCl_3$) δ_C, ppm: 170.4 (1C, S-(*Ar*)C-CH-CH-C-CH_2-(C=O)-O), 151.9, 151.8 (2C, 2xS-CH_2-(*Ar*)C-CH-CH-$\underline{C}$-C(CH_3)$_3$), 137.0 (1C, S-(*Ar*)$\underline{C}$-CH-CH-C-CH_2-(C=O)-O), 136.78, 136.75 (2C, 2xS-CH_2-(*Ar*)$\underline{C}$-CH-CH-C-C(CH_3)$_3$), 134.1 (1C, S-(*Ar*)C-CH-CH-$\underline{C}$-CH_2-(C=O)-O), 132.9 (2C, 2xS-(*Ar*)C-CH-$\underline{C}$H-C-CH_2-(C=O)-O), 130.1 (2C, 2xS-(*Ar*)C-$\underline{C}$H-CH-C-CH_2-(C=O)-O), 129.4, 129.2 (4C, 4xS-CH_2-(*Ar*)C-$\underline{C}$H-CH-C-C(CH_3)$_3$), 125.7, 125.5 (4C, 4xS-CH_2-(*Ar*)C-CH-$\underline{C}$H-C-C(CH_3)$_3$), 107.2 (2C, 2xCH_3-(*Ar*)C-CH-CH-$\underline{C}$), 100.7 (2C, 2xCH_3-(*Ar*)$\underline{C}$-CH-CH-C), 84.3 (2C, 2xCH_3-(*Ar*)C-CH-$\underline{C}$H-C), 83.7 (4C, 2xCH_3-(*Ar*)C-$\underline{C}$H-CH-C), 2xCH_3-(*Ar*)C-CH-$\underline{C}$H-C), 82.6 (2C, 2xCH_3-(*Ar*)C-$\underline{C}$H-CH-C), 77.2 (1C, (C=O)-O-CH_2-$\underline{C}$≡CH), 75.3 (1C, (C=O)-O-CH_2-C≡$\underline{C}$H), 52.6 (1C, (C=O)-O-$\underline{C}H_2$-C≡CH), 40.5 (1C, S-(*Ar*)C-CH-CH-C-$\underline{C}H_2$-(C=O)-O), 40.1 (1C, S-$\underline{C}H_2$-(*Ar*)C-CH-CH-C-C(CH_3)$_3$), 39.6 (1C, S-$\underline{C}H_2$-(*Ar*)C-CH-CH-C-C(CH_3)$_3$), 34.91, 34.86 (2C, 2xS-CH_2-(*Ar*)C-CH-CH-C-$\underline{C}$(CH_3)$_3$), 31.54, 31.53 (6C, 2xS-CH_2-(*Ar*)C-CH-CH-C-C($\underline{C}H_3$)$_3$), 30.9 (2C, 2x(*Ar*)CH-CH-C-$\underline{C}$H(CH_3)$_2$), 23.2 (2C, (*Ar*)CH-CH-C-CH($\underline{C}H_3$)$_2$), 22.7 (2C, (*Ar*)CH-CH-C-CH($\underline{C}H_3$)$_2$), 18.3 (2C, 2x$\underline{C}H_3$-(*Ar*)C-CH-CH).
R_f (CH_2Cl_2/CH_3OH 10:1) = 0.253.
ESI-MS(+): *m/z* found 1035.2361 $[M-Cl]^+$, calcd. for $C_{53}H_{67}O_2Ru_2S_3^+$ 1035.2385.
Elemental analysis (%): calcd. for $C_{53}H_{67}O_2Ru_2S_3Cl$ C 59.50, H 6.31; found C 59.48, H 6.31.

Synthesis of [(η^6-*p*-$MeC_6H_4Pr^i$)$_2Ru_2$(μ_2-$SCH_2C_6H_4$-*p*-Bu^t)$_2$(μ_2-SC_6H_4-*p*-CH_2-(C=O)-NH-R)]Cl (R = CH_2-C≡CH) (8)
Compound **8** was synthesized and purified as previously reported [5].

Synthesis of [(η^6-*p*-$MeC_6H_4Pr^i$)$_2Ru_2$(μ_2-$SCH_2C_6H_4$-*p*-Bu^t)$_2$(μ_2-SC_6H_4-*o*-$CH_2O(SO_2)CH_3$)]Cl (9A) and [(η^6-*p*-$MeC_6H_4Pr^i$)$_2Ru_2$(μ_2-$SCH_2C_6H_4$-*p*-Bu^t)$_2$(μ_2-SC_6H_4-*o*-CH_2N_3)]Cl (9)
To a solution of **4** (1.000 g, 0.996 mmol, 1 equiv.) in dry CH_2Cl_2 (50 mL) at 0°C under inert atmosphere (N_2), were added successively MsCl (0.115 mL, 1.492 mmol, 1.5 equiv.), and TEA (0.414 mL, 2.984 mmol, 3 equiv.). The mixture was further stirred at 0°C for another 2 h and then at r.t. overnight, the reaction evolution was verified by TLC. The reaction mixture was diluted with 100 mL CH_2Cl_2, washed with H_2O (100 mL) and brine (100 mL), dried over anhydrous Na_2SO_4 and concentrated under reduced pressure. The obtained mesylate **9A** was solubilized in dry DMF (20 mL) under inert atmosphere (N_2), and NaN_3 (0.240 g, 3.691 mmol, 4 equiv.) was added and the mixture was stirred at 60°C for 24 h. The reaction evolution was verified by TLC; the mixture was diluted with EtOAc (100 mL), washed with H_2O (200 mL) and brine (100 mL), dried over anhydrous Na_2SO_4 and concentrated under reduced pressure. Purification by column chromatography (CH_2Cl_2/CH_3OH 9.5:0.5 (v/v)) afforded **9** as an orange solid (0.678 g, 0.658 mmol, yield 66%).
9A:
^{1}H-NMR ($CDCl_3$) δ_H, ppm: 7.82 (1H, d, S-(*Ar*)C-C$\underline{H}$-CH-CH-CH, $^3J_{H,H}$ = 5.8 Hz), 7.39-7.52 (9H, m, 4xS-CH_2-(*Ar*)C-CH-C$\underline{H}$-C-C(CH_3)$_3$, 4xS-CH_2-(*Ar*)C-C$\underline{H}$-CH-C-C(CH_3)$_3$, S-(*Ar*)C-CH-CH-CH-C$\underline{H}$), 7.30-7.35 (2H, m, S-(*Ar*)C-CH-C$\underline{H}$-CH-CH, S-(*Ar*)C-CH-CH-C$\underline{H}$-CH, $^3J_{H,H}$ = 5.7 Hz), 5.13 (2H, d, 2xCH_3-(*Ar*)C-CH-C$\underline{H}$-C, $^3J_{H,H}$ = 3.3 Hz), 4.95 (2H, d, 2xCH_3-(*Ar*)C-CH-C$\underline{H}$-C, $^3J_{H,H}$ = 4.9 Hz), 4.84 (2H, d, 2xCH_3-(*Ar*)C-C$\underline{H}$-CH-C, $^3J_{H,H}$ = 3.8 Hz), 4.58 (2H, d, 2xCH_3-(*Ar*)C-C$\underline{H}$-CH-C, $^3J_{H,H}$ = 4.7 Hz), 3.65 (2H, s, C$\underline{H_2}$-(*Ar*)C-CH-CH-C-C(CH_3)$_3$), 3.42 (2H, s, C$\underline{H_2}$-(*Ar*)C-CH-CH-C-C(CH_3)$_3$), 3.14 (2H, s, S-(*Ar*)C-C-C$\underline{H_2}$-O-(SO_2)-CH_3), 2.91 (3H, s, S-(*Ar*)C-C-CH_2-O-(SO_2)-C$\underline{H_3}$), 1.94 (2H, sept, 2x(*Ar*)C-CH-CH-C-C$\underline{H}$(CH_3)$_2$, $^3J_{H,H}$ = 6.3 Hz), 1.68 (6H, s, 2xC$\underline{H_3}$-(*Ar*)C-CH-CH-C), 1.36 (9H, s, S-CH_2-(*Ar*)C-CH-CH-C-C(C$\underline{H_3}$)$_3$), 1.34 (9H, s, S-CH_2-(*Ar*)C-CH-CH-C-C(C$\underline{H_3}$)$_3$), 0.93 (6H, d, (*Ar*)C-CH-CH-C-CH(C$\underline{H_3}$)$_2$, $^3J_{H,H}$ = 5.8 Hz), 0.91 (6H, d, (*Ar*)C-CH-CH-C-CH(C$\underline{H_3}$)$_2$, $^3J_{H,H}$ = 6.1 Hz).
^{13}C-NMR ($CDCl_3$) δ_C, ppm: 152.0, 151.9 (2C, 2xS-CH_2-(*Ar*)C-CH-CH-$\underline{C}$-C(CH_3)$_3$), 140.9 (1C, S-(*Ar*)$\underline{C}$-C-CH-CH-CH-CH), 138.2 (1C, S-(*Ar*)C-$\underline{C}$-CH-CH-CH-CH), 136.7, 136.6 (2C, 2xS-CH_2-(*Ar*)$\underline{C}$-

CH-CH-C-C(CH$_3$)$_3$), 134.1 (1C, S-(*Ar*)C-$\underline{C}$H-CH-CH-CH),130.9 (1C, S-(*Ar*)C-CH-CH-CH-$\underline{C}$H), 129.9 (1C, S-(*Ar*)C-CH-CH-$\underline{C}$H-CH), 129.6, 129.2 (4C, 4xS-CH$_2$-(*Ar*)C-CH-$\underline{C}$H-C-C(CH$_3$)$_3$), 129.3 (1C, S-(*Ar*)C-CH-$\underline{C}$H-CH-CH), 125.8, 125.6 (4C, 4xS-CH$_2$-(*Ar*)C-$\underline{C}$H-CH-C-C(CH$_3$)$_3$), 107.0 (2C, 2xCH$_3$-(*Ar*)C-CH-CH-$\underline{C}$), 100.8 (2C, 2xCH$_3$-(*Ar*)$\underline{C}$-CH-CH-C), 83.7 (2C, 2xCH$_3$-(*Ar*)C-CH-$\underline{C}$H-C), 83.6 (2C, 2xCH$_3$-(*Ar*)C-$\underline{C}$H-CH-C), 83.5 (2C, 2xCH$_3$-(*Ar*)C-CH-$\underline{C}$H-C), 82.7 (2C, 2xCH$_3$-(*Ar*)C-$\underline{C}$H-CH-C), 60.3 (1C, S-(*Ar*)C-C-$\underline{C}$H$_2$-O-(SO$_2$)-CH$_3$), 40.3 (1C, S-$\underline{C}$H$_2$-(*Ar*)C-CH-CH-C-C(CH$_3$)$_3$), 39.5 (1C, S-(*Ar*)C-C-CH$_2$-O-(SO$_2$)-$\underline{C}$H$_3$), 38.9 (1C, S-$\underline{C}$H$_2$-(*Ar*)C-CH-CH-C-C(CH$_3$)$_3$), 34.90, 34.86 (2C, 2xS-CH$_2$-(*Ar*)C-CH-CH-C-$\underline{C}$(CH$_3$)$_3$), 31.52, 31.50 (6C, 2xS-CH$_2$-(*Ar*)C-CH-CH-C-C($\underline{C}$H$_3$)$_3$), 30.9 (2C, 2x(*Ar*)CH-CH-C-$\underline{C}$H(CH$_3$)$_2$), 23.0 (2C, (*Ar*)CH-CH-C-CH($\underline{C}$H$_3$)$_2$), 22.7 (2C, (*Ar*)CH-CH-C-CH($\underline{C}$H$_3$)$_2$), 18.3 (2C, 2x$\underline{C}$H$_3$-(*Ar*)C-CH-CH).
R*f* (CH$_2$Cl$_2$/MeOH 9.5:0.5) = 0.414.

9:
^{1}H-NMR (CDCl$_3$) δ*H*, ppm: 7.85-7.90 (1H, m, S-(*Ar*)C-C$\underline{H}$-CH-CH-CH), 7.42-7.50 (8H, m, 4xS-CH$_2$-(*Ar*)C-CH-C$\underline{H}$-C-C(CH$_3$)$_3$, 4xS-CH$_2$-(*Ar*)C-C$\underline{H}$-CH-C-C(CH$_3$)$_3$), 7.28-7.37 (3H, m, S-(*Ar*)C-CH-C$\underline{H}$-CH-CH, S-(*Ar*)C-CH-CH-C$\underline{H}$-CH, S-(*Ar*)C-CH-CH-CH-C$\underline{H}$), 5.16 (2H, d, 2xCH$_3$-(*Ar*)C-CH-C$\underline{H}$-C, $^3J_{H,H}$ = 5.7 Hz), 5.00 (2H, d, 2xCH$_3$-(*Ar*)C-CH-C$\underline{H}$-C, $^3J_{H,H}$ = 5.8 Hz), 4.96 (2H, s, S-(*Ar*)C-C-C$\underline{H_2}$-N$_3$), 4.92 (2H, d, 2xCH$_3$-(*Ar*)C-C$\underline{H}$-CH-C, $^3J_{H,H}$ = 5.7 Hz), 4.62 (2H, d, 2xCH$_3$-(*Ar*)C-C$\underline{H}$-CH-C, $^3J_{H,H}$ = 5.8 Hz), 3.67 (2H, s, C$\underline{H_2}$-(*Ar*)C-CH-CH-C-C(CH$_3$)$_3$), 3.45 (2H, s, C$\underline{H_2}$-(*Ar*)C-CH-CH-C-C(CH$_3$)$_3$), 1.90 (2H, sept, 2x(*Ar*)C-CH-CH-C-C$\underline{H}$(CH$_3$)$_2$, $^3J_{H,H}$ = 6.9 Hz), 1.69 (6H, s, 2xC$\underline{H_3}$-(*Ar*)C-CH-CH-C), 1.36 (9H, s, S-CH$_2$-(*Ar*)C-CH-CH-C-C(C$\underline{H_3}$)$_3$), 1.33 (9H, s, S-CH$_2$-(*Ar*)C-CH-CH-C-C(C$\underline{H_3}$)$_3$), 0.92 (6H, d, (*Ar*)C-CH-CH-C-CH(C$\underline{H_3}$)$_2$, $^3J_{H,H}$ = 6.9 Hz), 0.90 (6H, d, (*Ar*)C-CH-CH-C-CH(C$\underline{H_3}$)$_2$, $^3J_{H,H}$ = 6.9 Hz).
^{13}C-NMR (CDCl$_3$) δ*C*, ppm: 151.9, 151.8 (2C, 2xS-CH$_2$-(*Ar*)C-CH-CH-$\underline{C}$-C(CH$_3$)$_3$), 139.0 (1C, S-(*Ar*)$\underline{C}$-C-CH-CH-CH-CH), 138.3 (1C, S-(*Ar*)C-$\underline{C}$-CH-CH-CH-CH), 136.8, 136.6 (2C, 2xS-CH$_2$-(*Ar*)$\underline{C}$-CH-CH-C-C(CH$_3$)$_3$), 134.3 (1C, S-(*Ar*)C-$\underline{C}$H-CH-CH-CH), 130.3 (1C, S-(*Ar*)C-CH-CH-CH-$\underline{C}$H), 129.8 (1C, S-(*Ar*)C-CH-CH-$\underline{C}$H-CH), 129.6, 129.2 (4C, 4xS-CH$_2$-(*Ar*)C-CH-$\underline{C}$H-C-C(CH$_3$)$_3$), 129.0 (1C, S-(*Ar*)C-CH-$\underline{C}$H-CH-CH), 125.7, 125.6 (4C, 4xS-CH$_2$-(*Ar*)C-$\underline{C}$H-CH-C-C(CH$_3$)$_3$), 106.9 (2C, 2xCH$_3$-(*Ar*)C-CH-CH-$\underline{C}$), 100.9 (2C, 2xCH$_3$-(*Ar*)$\underline{C}$-CH-CH-C), 83.9 (2C, 2xCH$_3$-(*Ar*)C-CH-$\underline{C}$H-C), 83.7 (2C, 2xCH$_3$-(*Ar*)C-$\underline{C}$H-CH-C), 83.5 (2C, 2xCH$_3$-(*Ar*)C-CH-$\underline{C}$H-C), 82.7 (2C, 2xCH$_3$-(*Ar*)C-$\underline{C}$H-CH-C), 53.8 (1C, s, S-(*Ar*)C-C-$\underline{C}$H$_2$-N$_3$), 40.3 (1C, S-$\underline{C}$H$_2$-(*Ar*)C-CH-CH-C-C(CH$_3$)$_3$), 39.1 (1C, S-$\underline{C}$H$_2$-(*Ar*)C-CH-CH-C-C(CH$_3$)$_3$), 34.91, 34.87 (2C, 2xS-CH$_2$-(*Ar*)C-CH-CH-C-$\underline{C}$(CH$_3$)$_3$), 31.6 (6C, 2xS-CH$_2$-(*Ar*)C-CH-CH-C-C($\underline{C}$H$_3$)$_3$), 30.9 (2C, 2x(*Ar*)CH-CH-C-$\underline{C}$H(CH$_3$)$_2$), 23.0 (2C, (*Ar*)CH-CH-C-CH($\underline{C}$H$_3$)$_2$), 22.7 (2C, (*Ar*)CH-CH-C-CH($\underline{C}$H$_3$)$_2$), 18.3 (2C, 2x$\underline{C}$H$_3$-(*Ar*)C-CH-CH).
R*f* (CH$_2$Cl$_2$/CH$_3$OH 9.5:0.5 (v/v)) = 0.410.
ESI-MS(+): m/z found 994.2329 [M-Cl]$^+$, calcd. for $C_{49}H_{64}N_3Ru_2S_3^+$ 994.2344.
Elemental analysis (%): calcd. for $C_{49}H_{64}ClN_3Ru_2S_3$·0.25CH_2Cl_2 C 56.33, H 6.19, N 4.00; found C 56.20, H 5.73, N 4.33.

Synthesis of [(η^6-*p*-MeC$_6$H$_4$Pr^i)$_2$Ru$_2$(μ_2-SCH$_2$C$_6$H$_4$-*p*-Bu^t)$_2$(μ_2-SC$_6$H$_4$-*p*-N$_3$)]Cl (10)
Compound **10** was prepared by adapting a literature procedure [6]. To a suspension of **2** (0.300 g, 0.303 mmol, 1 equiv.) in HCl aq. (17%, 10 mL), $NaNO_2$ (0.025 g, 0.364 mmol, 1.2 equiv.) was added at 0°C under inert atmosphere (N_2), and the mixture was further stirred for 1 h. Then NaN_3 (0.024 g, 0.364 mmol, 1.2 equiv.) was added and the reaction mixture was further stirred at 0°C under N_2 for another 5 h, and subsequently at r.t. for 18 h. The reaction evolution was verified by TLC. The reaction mixture was filtered, and precipitate was solubilised in CH_2Cl_2 (30 mL), and the organic solution was

washed with H_2O (2x30 mL), with brine (30 mL), dried over anhydrous Na_2SO_4, filtered and concentrated to dryness. Compound **10** (0.325 g) was recovered as an orange solid still containing traces of the starting amine **2** and was used in click reactions without further purification. A small quantity was purified by column chromatography to be used for the biological activity assessment.

^{1}H-NMR ($CDCl_3$) δ_H, ppm: 7.87 (2H, d, 2xS-(*Ar*)C-C$\underline{H}$-CH-C-N_3, $^3J_{H,H}$ = 8.3 Hz), 7.41-7.54 (8H, m, 4xS-CH_2-(*Ar*)C-C$\underline{H}$-CH-C-C$(CH_3)_3$, 4xS-CH_2-(*Ar*)C-CH-C$\underline{H}$-C-C$(CH_3)_3$, $^3J_{H,H}$ = 8.6 Hz), 7.01 (2H, d, 2xS-(*Ar*)C-CH-C$\underline{H}$-C-N_3, $^3J_{H,H}$ = 8.3 Hz), 5.13 (2H, d, 2xCH_3-(*Ar*)C-CH-C$\underline{H}$-C, $^3J_{H,H}$ = 5.6 Hz), 5.05 (2H, d, 2xCH_3-(*Ar*)C-CH-C$\underline{H}$-C, $^3J_{H,H}$ = 5.9 Hz), 4.91 (2H, d, 2xCH_3-(*Ar*)C-C$\underline{H}$-CH-C, $^3J_{H,H}$ = 5.6 Hz), 4.66 (2H, d, 2xCH_3-(*Ar*)C-C$\underline{H}$-CH-C, $^3J_{H,H}$ = 5.7 Hz), 3.67 (2H, s, S-C$\underline{H_2}$-(*Ar*)C-CH-CH-C-C$(CH_3)_3$), 3.44 (2H, s, S-C$\underline{H_2}$-(*Ar*)C-CH-CH-C-C$(CH_3)_3$), 1.95 (2H, sept, 2x(*Ar*)C-CH-CH-C-C$\underline{H}(CH_3)_2$, $^3J_{H,H}$ = 6.8 Hz), 1.75 (6H, s, 2xC$\underline{H_3}$-(*Ar*)C-CH-CH-C), 1.36 (9H, s, S-CH_2-(*Ar*)C-CH-CH-C-C$(C\underline{H_3})_3$), 1.34 (9H, s, S-CH_2-(*Ar*)C-CH-CH-C-C$(C\underline{H_3})_3$), 0.97 (6H, d, (*Ar*)C-CH-CH-C-CH$(C\underline{H_3})_2$, $^3J_{H,H}$ = 6.8 Hz), 0.92 (6H, d, (*Ar*)C-CH-CH-C-CH$(C\underline{H_3})_2$, $^3J_{H,H}$ = 6.8 Hz).

^{13}C-NMR ($CDCl_3$) δ_H, ppm: 151.82, 151.76 (2C, 2xS-CH_2-(*Ar*)C-CH-CH-$\underline{C}$-C$(CH_3)_3$), 140.6 (1C, S-(*Ar*)C-CH-CH-$\underline{C}$-N_3), 136.8 (2C, 2xS-CH_2-(*Ar*)$\underline{C}$-CH-CH-C-C$(CH_3)_3$), 134.5 (2C, 2xS-(*Ar*)C-$\underline{C}$H-CH-C-N_3), 134.4 (1C, S-(*Ar*)$\underline{C}$-CH-CH-C-N_3), 129.6, 129.3 (4C, 4xS-CH_2-(*Ar*)C-$\underline{C}$H-CH-C-C$(CH_3)_3$), 125.7, 125.6 (4C, 4xS-CH_2-(*Ar*)C-CH-$\underline{C}$H-C-C$(CH_3)_3$), 119.6 (2C, 2xS-(*Ar*)C-CH-$\underline{C}$H-C-N_3), 107.3 (2C, 2xCH_3-(*Ar*)C-CH-CH-$\underline{C}$), 100.6 (2C, 2xCH_3-(*Ar*)$\underline{C}$-CH-CH-C), 84.0 (4C, 2xCH_3-(*Ar*)C-$\underline{C}$H-CH-C, 2xCH_3-(*Ar*)C-CH-$\underline{C}$H-C), 83.9 (2C, 2xCH_3-(*Ar*)C-CH-$\underline{C}$H-C), 82.6 (2C, 2xCH_3-(*Ar*)C-$\underline{C}$H-CH-C), 40.2 (1C, S-$\underline{C}H_2$-(*Ar*)C-CH-CH-C-C$(CH_3)_3$), 39.8 (1C, S-$\underline{C}H_2$-(*Ar*)C-CH-CH-C-C$(CH_3)_3$), 34.92, 34.89 (2C, 2xS-CH_2-(*Ar*)C-CH-CH-C-$\underline{C}(CH_3)_3$), 31.58, 31.56 (6C, 2xS-CH_2-(*Ar*)C-CH-CH-C-C$(\underline{C}H_3)_3$), 31.1 (2C, 2x(*Ar*)CH-CH-C-$\underline{C}H(CH_3)_2$), 23.2 (2C, (*Ar*)CH-CH-C-CH$(\underline{C}H_3)_2$), 22.9 (2C, (*Ar*)CH-CH-C-CH$(\underline{C}H_3)_2$), 18.4 (2C, 2x$\underline{C}H_3$-(*Ar*)C-CH-CH).

R_f (CH_2Cl_2/CH_3OH 10:1) = 0.327.

ESI-MS(+): *m/z* found 980.2222 $[M-Cl]^+$, calcd. for $C_{48}H_{62}N_3Ru_2S_3^+$ 980.2187.

Elemental analysis (%): calcd. for $C_{48}H_{62}ClN_3Ru_2S_3\cdot0.5CH_2Cl_2\cdot2CH_3OH$ C 54.09, H 6.38, N 3.75; found: C 54.04, H 6.43, N 3.95.

3. Synthesis of the compounds **11-13** (family 1)

Synthesis of $[(\eta^6\text{-}p\text{-}MeC_6H_4Pr^i)_2Ru_2(\mu_2\text{-}SCH_2C_6H_4\text{-}p\text{-}Bu^t)_2(\mu_2\text{-}SR)]Cl$ (R = 4-aminopyrimidin-2-yl) (11)

Compound **11** was prepared and purified by adapting a literature procedure [2,3,7]. To a solution of **1** (0.300 g, 0.333 mmol, 1 equiv.) in EtOH (200 mL) was added 2-thiocytosine (4-aminopyrimidine-2-thiol) (0.175 g, 1.332 mmol, 4 equiv.), the suspension was heated at reflux for 96 h and the reaction evolution was verified by TLC. The reaction mixture was concentrated to dryness under reduced pressure. Purification by column chromatography (CH_2Cl_2/CH_3OH 9.5:0.5 (v/v)) afforded **11** as a brown solid (0.044 g, 0.044 mmol, yield 13%).

^{1}H-NMR ($CDCl_3$) δ_H, ppm: 8.02 (1H, d, S-(*Ar*)C-N-C$\underline{H}$-CH, $^3J_{H,H}$ = 5.4 Hz), 7.37-7.59 (8H, m, 4xS-CH_2-(*Ar*)C-C$\underline{H}$-CH-C-C$(CH_3)_3$, 4xS-CH_2-(*Ar*)C-CH-C$\underline{H}$-C-C$(CH_3)_3$), 6.40 (1H, d, S-(*Ar*)C-N-CH-C$\underline{H}$, $^3J_{H,H}$ = 5.9 Hz), 5.19 (2H, d, 2xCH_3-(*Ar*)C-CH-C$\underline{H}$-C, $^3J_{H,H}$ = 5.8 Hz), 5.16 (2H, d, 2xCH_3-(*Ar*)C-CH-C$\underline{H}$-C, $^3J_{H,H}$ = 5.7 Hz), 4.84-4.94 (4H, m br, 4xCH_3-(*Ar*)C-$\underline{CH}$-CH-C), 3.58 (2H, s br, S-C$\underline{H_2}$-(*Ar*)C-CH-CH-C-C$(CH_3)_3$), 3.49 (2H, s, S-C$\underline{H_2}$-(*Ar*)C-CH-CH-C-C$(CH_3)_3$), 2.46 (2H, sept, 2x(*Ar*)C-CH-CH-C-C$\underline{H}(CH_3)_2$, $^3J_{H,H}$ = 7.0 Hz), 1.95 (6H, s, 2xC$\underline{H_3}$-(*Ar*)C-CH-CH-C), 1.37 (9H, s, S-CH_2-(*Ar*)C-CH-CH-C-C$(C\underline{H_3})_3$), 1.35 (9H, s, S-CH_2-(*Ar*)C-CH-CH-C-C$(C\underline{H_3})_3$), 1.08 (6H, d, (*Ar*)C-CH-CH-C-CH$(C\underline{H_3})_2$, $^3J_{H,H}$ = 6.8 Hz), 0.94-1.04 (6H, m, (*Ar*)C-CH-CH-C-CH$(C\underline{H_3})_2$).

R_f (CH_2Cl_2/CH_3OH 10:1) = 0.170.

ESI-MS(+): *m/z* found 956.2179 [M-Cl]$^+$, calcd. for $C_{46}H_{62}N_3Ru_2S_3^+$ 956.2187.
Elemental analysis (%): calcd. for $C_{46}H_{62}ClN_3Ru_2S_3 \cdot CH_3OH$ C 55.19, H 6.50, N 4.11; found C 55.24, H 6.52, N 4.83.

Synthesis of [(η^6-*p*-MeC$_6$H$_4$Pr^i)$_2$Ru$_2$(μ_2-SCH$_2$C$_6$H$_4$-*p*-Bu^t)$_2$(μ_2-SR)]Cl (R = 2-oxo-1,2-dihydropyrimidin-4-yl (12)

Compound **12** was prepared and purified by adapting a literature procedure [2,3,7]. To a solution of dithiolato precursor **1** (0.300 g, 0.333 mmol, 1 equiv.) in EtOH (130 mL) at reflux was added a suspension of 4-thiouracil (4-mercaptopyrimidin-2(1*H*)-one) (0.064 g, 0.500 mmol, 1.5 equiv.) in EtOH (20 mL), and the mixture was further heated at reflux for 18 h and the reaction evolution was verified by TLC. The reaction mixture was concentrated to dryness under reduced pressure. Purification by column chromatography (CH_2Cl_2/CH_3OH 10:1 (v/v)) afforded as an orange solid (0.076 g, 0.077 mmol, yield 23%).

^{1}H-NMR (CDCl$_3$) δ_H, ppm: 11.93 (1H, s br, N$\underline{H}$-(C=O)-N), 8.42 (1H, d, S-C-CH-C$\underline{H}$, $^3J_{H,H}$ = 4.9 Hz), 7.35-7.52 (8H, m, 4xS-CH$_2$-(*Ar*)C-CH-C$\underline{H}$-C-C(CH$_3$)$_3$, 4xS-CH$_2$-(*Ar*)C-C$\underline{H}$-CH-C-C(CH$_3$)$_3$), 6.87 (1H, d, S-C-C$\underline{H}$-CH, $^3J_{H,H}$ = 4.9 Hz), 5.34 (2H, d, 2xCH$_3$-(*Ar*)C-CH-C$\underline{H}$-C, $^3J_{H,H}$ = 4.6 Hz), 5.09 (4H, m, 2xCH$_3$-(*Ar*)C-CH-C$\underline{H}$-C, 2xCH$_3$-(*Ar*)C-C$\underline{H}$-CH-C), 4.66 (2H, d, 2xCH$_3$-(*Ar*)C-C$\underline{H}$-CH-C, $^3J_{H,H}$ = 5.1 Hz), 3.61 (2H, s, S-C$\underline{H_2}$-(*Ar*)C-CH-CH-C-C(CH$_3$)$_3$), 3.37 (2H, s, S-C$\underline{H_2}$-(*Ar*)C-CH-CH-C-C(CH$_3$)$_3$), 2.20 (2H, sept, 2x(*Ar*)C-CH-CH-C-C$\underline{H}$(CH$_3$)$_2$, $^3J_{H,H}$ = 6.9 Hz), 1.94 (6H, s, 2xC$\underline{H_3}$-(*Ar*)C-CH-CH-C), 1.34 (9H, s, S-CH$_2$-(*Ar*)C-CH-CH-C-C(C$\underline{H_3}$)$_3$), 1.32 (9H, s, S-CH$_2$-(*Ar*)C-CH-CH-C-C(C$\underline{H_3}$)$_3$), 1.03 (6H, d, (*Ar*)C-CH-CH-C-CH(C$\underline{H_3}$)$_2$, $^3J_{H,H}$ = 6.7 Hz), 0.98 (6H, d, (*Ar*)C-CH-CH-C-CH(C$\underline{H_3}$)$_2$, $^3J_{H,H}$ = 6.8 Hz).
R$_f$ (CH_2Cl_2/CH_3OH 10:1) = 0.273.
ESI-MS(+): *m/z* found 957.2065 [M-Cl]$^+$, calcd. for $C_{46}H_{61}N_2ORu_2S_3^+$ 957.2028.

Synthesis of [(η^6-*p*-MeC$_6$H$_4$Pr^i)$_2$Ru$_2$(μ_2-SCH$_2$C$_6$H$_4$-*p*-Bu^t)$_2$(μ_2-SR)]Cl (R = 6-oxo-6,9-dihydro-1*H*-purin-2-yl*)* (13)

Compound **13** was prepared and purified by adapting a literature procedure [2,3,7]. To a solution of **1** (0.300 g, 0.333 mmol, 1 equiv.) in EtOH (50 mL) was added 2-thioxanthine (2-mercapto-1,9-dihydro-6*H*-purin-6-one) (0.171 g, 0.999 mmol, 3 equiv.), The suspension was heated under reflux for 24 h and the reaction evolution was verified by TLC. The reaction mixture was filtered and the filtrate was concentrated to dryness under reduced pressure. Purification by column chromatography (CH_2Cl_2/CH_3OH 9:1 (v/v)) afforded **13** as an orange solid (0.152 g, 0.147 mmol, yield 44%).

^{1}H-NMR (CDCl$_3$) δ_H, ppm: 8.11 (1H, s, S-(*Ar*)C-N-C-NH-C$\underline{H}$-N), 7.40-7.46 (4H, m, 2xS-CH$_2$-(*Ar*)C-CH-C$\underline{H}$-C-C(CH$_3$)$_3$, 2xS-CH$_2$-(*Ar*)C-C$\underline{H}$-CH-C-C(CH$_3$)$_3$), 7.33-7.40 (4H, m, 2xS-CH$_2$-(*Ar*)C-C$\underline{H}$-CH-C-C(CH$_3$)$_3$), 2xS-CH$_2$-(*Ar*)C-CH-C$\underline{H}$-C-C(CH$_3$)$_3$), 5.50 (2H, d, 2xCH$_3$-(*Ar*)C-CH-C$\underline{H}$-C, $^3J_{H,H}$ = 5.0 Hz), 5.42 (2H, d, 2xCH$_3$-(*Ar*)C-CH-C$\underline{H}$-C, $^3J_{H,H}$ = 5.4 Hz), 5.04 (2H, d, 2xCH$_3$-(*Ar*)C-C$\underline{H}$-CH-C, $^3J_{H,H}$ = 5.6 Hz), 4.54 (2H, d, 2xCH$_3$-(*Ar*)C-C$\underline{H}$-CH-C, $^3J_{H,H}$ = 4.7 Hz), 3.67 (2H, s, S-C$\underline{H_2}$-(*Ar*)C-CH-CH-C-C(CH$_3$)$_3$), 3.32 (2H, s, S-C$\underline{H_2}$-(*Ar*)C-CH-CH-C-C(CH$_3$)$_3$), 2.11 (2H, sept, 2x(*Ar*)C-CH-CH-C-C$\underline{H}$(CH$_3$)$_2$, $^3J_{H,H}$ = 6.7 Hz), 1.92 (6H, s, 2xC$\underline{H_3}$-(*Ar*)C-CH-CH-C), 1.32 (9H, s, S-CH$_2$-(*Ar*)C-CH-CH-C-C(C$\underline{H_3}$)$_3$), 1.27 (9H, s, S-CH$_2$-(*Ar*)C-CH-CH-C-C(C$\underline{H_3}$)$_3$), 0.84 (6H, d, (*Ar*)C-CH-CH-C-CH(C$\underline{H_3}$)$_2$, $^3J_{H,H}$ = 6.7 Hz), 0.81 (6H, d, (*Ar*)C-CH-CH-C-CH(C$\underline{H_3}$)$_2$, $^3J_{H,H}$ = 6.8 Hz).

^{13}C-NMR (CDCl$_3$) δ_C, ppm: 157.5 (1C, S-(*Ar*)C-NH-$\underline{C}$=O), 155.2 (1C, S-(*Ar*)C-N-$\underline{C}$-NH-CH-N), 153.3 (1C, S-(*Ar*)$\underline{C}$-NH-C=O), 151.84, 151.79 (2C, 2xS-CH$_2$-(*Ar*)C-CH-CH-$\underline{C}$-C(CH$_3$)$_3$), 141.3 (1C, S-(*Ar*)C-N-C-NH-$\underline{C}$H-N), 137.0, 136.7 (2C, 2xS-CH$_2$-(*Ar*)$\underline{C}$-CH-CH-C-C(CH$_3$)$_3$), 129.3, 129.1 (4C, 4xS-CH$_2$-(*Ar*)C-$\underline{C}$H-CH-C-C(CH$_3$)$_3$), 125.6, 125.5 (4C, 4xS-CH$_2$-(*Ar*)C-CH-$\underline{C}$H-C-C(CH$_3$)$_3$), 118.4 (1C, S-

(*Ar*)C-NH-(C=O)-$\underline{C}$-N), 106.6 (2C, 2xCH_3-(*Ar*)C-CH-CH-$\underline{C}$), 102.4 (2C, 2xCH_3-(*Ar*)$\underline{C}$-CH-CH-C), 85.2 (2C, 2xCH_3-(*Ar*)C-CH-$\underline{C}$H-C), 84.6 (2C, 2xCH_3-(*Ar*)C-$\underline{C}$H-CH-C), 83.3 (2C, 2xCH_3-(*Ar*)C-$\underline{C}$H-CH-C), 82.3 (2C, 2xCH_3-(*Ar*)C-CH-$\underline{C}$H-C), 40.4 (1C, S-$\underline{C}H_2$-(*Ar*)C-CH-CH-C-C$(CH_3)_3$), 40.3 (1C, S-$\underline{C}H_2$-(*Ar*)C-CH-CH-C-C$(CH_3)_3$), 34.8 (2C, 2xS-CH_2-(*Ar*)C-CH-CH-C-$\underline{C}(CH_3)_3$), 31.43, 31.41 (6C, 2xS-CH_2-(*Ar*)C-CH-CH-C-C$(\underline{C}H_3)_3$), 31.3 (2C, 2x(*Ar*)CH-CH-C-$\underline{C}H(CH_3)_2$), 23.1 (2C, (*Ar*)CH-CH-C-CH$(\underline{C}H_3)_2$), 22.5 (2C, (*Ar*)CH-CH-C-CH$(\underline{C}H_3)_2$), 18.5 (2C, 2x$\underline{C}H_3$-(*Ar*)C-CH-CH).
R*f* (CH_2Cl_2/CH_3OH 10:1) = 0.402.
ESI-MS(+): *m/z* found 997.2109 $[M-Cl]^+$, calcd. for $C_{47}H_{61}N_4ORu_2S_3^+$ 997.2089.
Elemental analysis (%): calcd. for $C_{47}H_{61}ClN_4ORu_2S_3\cdot 1.5CH_2Cl_2$ C 50.25, H 5.57, N 4.83; found C 50.98, H 5.34, N 4.82.

4. Synthesis of the compound **14** (family 2)

Synthesis of [(η^6-*p*-$MeC_6H_4Pr^i$)$_2$$Ru_2$($\mu_2$-$SCH_2C_6H_4$-p-$Bu^t$)$_2$($\mu_2$-$SC_6H_4$-*p*-$CH_2CO_2R$)]Cl (R = 2-(6-amino-9*H*-purin-9-yl)ethyl) (14)

Compound **14** was prepared and purified by adapting a literature protocol [2]. To a solution of **3** (0.300 g, 0.291 mmol, 1 equiv.) in dry CH_2Cl_2 (50 mL) at r.t. under inert atmosphere (N_2) were added successively EDCI (0.112 g, 0.582 mmol, 2 equiv.), 9-(2-hydroxyethyl)adenine (2-(6-amino-9*H*-purin-9-yl)ethan-1-ol) (**14A**) (0.080 g, 0.437 mmol, 1.5 equiv.) and DMAP (0.018 g, 0.146 mmol, 0.5 equiv.). The reaction mixture was stirred at r.t. under inert atmosphere (N_2) for 72 h and the reaction evolution was verified by TLC. The reaction mixture was concentrated to dryness under reduced pressure. Purification by column chromatography (CH_2Cl_2/CH_3OH 9:1 (v/v)) afforded **14** as an orange solid (0.148 g, 0.124 mmol, yield 43%).
^{1}H-NMR (MeOD-*d$_4$*) δ_H, ppm: 8.25 (1H, s, N-C$\underline{H}$-N-C-NH_2), 8.19 (1H, s, CH_2-N-C$\underline{H}$-N-C-C-NH_2), 7.71 (2H, d, S-(*Ar*)C-CH-C$\underline{H}$-C-CH_2-(C=O)-O, $^3J_{H,H}$ = 8.1 Hz), 7.55 (2H, d, 2xS-CH_2-(*Ar*)C-CH-C$\underline{H}$-C-$C(CH_3)_3$, $^3J_{H,H}$ = 8.3 Hz), 7.50 (4H, d, 2xS-CH_2-(*Ar*)C-CH-C$\underline{H}$-C-$C(CH_3)_3$, 2xS-CH_2-(*Ar*)C-C$\underline{H}$-CH-C-$C(CH_3)_3$, $^3J_{H,H}$ = 8.4 Hz), 7.45 (2H, d, 2xS-CH_2-(*Ar*)C-C$\underline{H}$-CH-C-$C(CH_3)_3$, $^3J_{H,H}$ = 8.4 Hz), 7.08 (2H, d, S-(*Ar*)C-C$\underline{H}$-CH-C-CH_2-(C=O)-O, $^3J_{H,H}$ = 8.2 Hz), 5.20 (2H, d, 2xCH_3-(*Ar*)C-CH-C$\underline{H}$-C, $^3J_{H,H}$ = 5.8 Hz), 5.11 (2H, d, 2xCH_3-(*Ar*)C-CH-C$\underline{H}$-C, $^3J_{H,H}$ = 5.9 Hz), 5.04 (2H, d, 2xCH_3-(*Ar*)C-C$\underline{H}$-CH-C, $^3J_{H,H}$ = 5.8 Hz), 4.69 (2H, d, 2xCH_3-(*Ar*)C-C$\underline{H}$-CH-C, $^3J_{H,H}$ = 5.9 Hz), 4.56 (2H, d, (C=O)-O-C$\underline{H_2}$-CH_2-N, $^3J_{H,H}$ = 4.9 Hz), 4.48 (2H, d, (C=O)-O-CH_2-C$\underline{H_2}$-N, $^3J_{H,H}$ = 4.9 Hz), 3.68 (2H, s, S-C$\underline{H_2}$-(*Ar*)C-CH-CH-C-$(CH_3)_3$), 3.61 (2H, s, S-(*Ar*)C-CH-CH-C-C$\underline{H_2}$-(C=O)-O), 3.49 (2H, s, S-C$\underline{H_2}$-(*Ar*)C-CH-CH-C-$(CH_3)_3$), 1.80 (2H, sept, 2x(*Ar*)C-CH-CH-C-C$\underline{H}(CH_3)_2$, $^3J_{H,H}$ = 6.8 Hz), 1.73 (6H, s, 2xC$\underline{H_3}$-(*Ar*)C-CH-CH-C), 1.39 (9H, s, S-CH_2-(*Ar*)C-CH-CH-C-C(C$\underline{H_3}$)$_3$), 1.35 (9H, s, S-CH_2-(*Ar*)C-CH-CH-C-C(C$\underline{H_3}$)$_3$), 0.85 (6H, d, 2x(*Ar*)C-CH-CH-C-CH(C$\underline{H_3}$)$_2$, $^3J_{H,H}$ = 6.8 Hz), 0.84 (6H, d, 2x(*Ar*)C-CH-CH-C-CH(C$\underline{H_3}$)$_2$, $^3J_{H,H}$ = 6.9 Hz).
^{13}C-NMR (MeOD-*d$_4$*) δ_C, ppm: 172.3 (1C, S-(*Ar*)C-CH-CH-C-CH_2-($\underline{C}$=O)-O), 157.4 (1C, N-CH-N-C-$\underline{C}$-NH_2), 153.9 (1C, N-$\underline{C}$H-N-C-NH_2), 152.8, 152.6 (2C, 2xS-CH_2-(*Ar*)C-CH-CH-$\underline{C}$-$C(CH_3)_3$), 150.9 (1C, N-$\underline{C}$-N-$(CH_2)_2$), 143.1 (1C, N-$\underline{C}$H-N-C-C-NH_2), 138.6, 138.5 (2C, 2xS-CH_2-(*Ar*)$\underline{C}$-CH-CH-C-$C(CH_3)_3$), 138.1 (1C, S-(*Ar*)$\underline{C}$-CH-CH-C-CH_2-(C=O)-O), 135.8 (1C, S-(*Ar*)C-CH-CH-$\underline{C}$-CH_2-(C=O)-O), 134.1 (2C, 2xS-(*Ar*)C-CH-$\underline{C}$H-C-CH_2-(C=O)-O), 130.8, 130.7 (4C, 4xS-CH_2-(*Ar*)C-$\underline{C}$H-CH-C-$C(CH_3)_3$), 130.3 (2C, 2xS-(*Ar*)C-$\underline{C}$H-CH-C-CH_2-(C=O)-O), 125.8, 125.6 (4C, 4xS-CH_2-(*Ar*)C-CH-$\underline{C}$H-C-$C(CH_3)_3$), 120.1 (1C, N-CH-N-$\underline{C}$-C-NH_2), 107.8 (2C, 2xCH_3-(*Ar*)C-CH-CH-$\underline{C}$), 102.5 (2C, 2xCH_3-(*Ar*)$\underline{C}$-CH-CH-C), 86.1 (2C, 2xCH_3-(*Ar*)C-CH-$\underline{C}$H-C), 84.51 (2C, 2xCH_3-(*Ar*)C-$\underline{C}$H-CH-C), 84.47 (2C, 2xCH_3-(*Ar*)C-CH-$\underline{C}$H-C), 83.9 (2C, 2xCH_3-(*Ar*)C-$\underline{C}$H-CH-C), 64.0 (1C, (C=O)-O-CH_2-$\underline{C}H_2$), 44.0 (1C, (C=O)-O-$\underline{C}H_2$-CH_2), 41.2 (1C, S-$\underline{C}H_2$-(*Ar*)C-CH-CH-C-$C(CH_3)_3$), 41.1 (1C, S-$\underline{C}H_2$-(*Ar*)C-CH-CH-C-$C(CH_3)_3$), 40.6 (1C, S-(*Ar*)C-CH-CH-C-$\underline{C}H_2$-(C=O)-O), 35.63, 35.58 (2C, 2xS-CH_2-(*Ar*)C-CH-CH-C-

$\underline{C}(CH_3)_3$), 31.9 (2C, 2x(*Ar*)CH-CH-C-$\underline{C}H(CH_3)_2$), 31.84, 31.82 (6C, 2xS-CH2-(*Ar*)C-CH-CH-C-C($\underline{C}H_3)_3$), 23.5 (2C, (*Ar*)CH-CH-C-CH($\underline{C}H_3)_2$), 22.9 (2C, (*Ar*)CH-CH-C-CH($\underline{C}H_3)_2$), 18.2 (2C, 2x$\underline{C}H_3$-(*Ar*)C-CH-CH).
R_f (CH_2Cl_2/CH_3OH 10:1) = 0.408.
ESI-MS(+): *m/z* found 1158.2926 [M-Cl]$^+$, calcd. for $C_{57}H_{72}N_5O_2Ru_2S_3^+$ 1158.2930.
Elemental analysis (%): calcd. for $C_{57}H_{72}ClN_5O_2Ru_2S_3 \cdot 0.5CH_2Cl_2 \cdot H_2O$ C 55.10, H 6.03, N 5.59; found C 55.09, H 6.01, N 5.11.

5. Synthesis of the compounds **15-26** (family 3)
The nucleic base derivatives **15-18** were prepared and purified by adapting literature procedures [8-10].
Synthesis of 1-propargyl-uracil (1-(prop-2-yn-1-yl)pyrimidine-2,4(1*H*,3*H*)-dione) (15)
To a solution of uracil (pyrimidine-2,4(1*H*,3*H*)-dione) (0.500 g, 4.371 mmol, 1 equiv.) in dry DMF (20 mL) at 60°C, under inert atmosphere (N_2) was added K_2CO_3 (0.302 g, 2.186 mmol, 0.5 equiv.) followed by dropwise addition of propargyl bromide (0.470 mL, 4.371 mmol, 1 equiv.). The mixture was stirred at 60°C for 48 h and the reaction evolution was verified by TLC. The reaction mixture was allowed to cool at r.t. and was concentrated under reduced pressure. Purification by column chromatography (CH_2Cl_2/CH_3OH 10:1 (v/v)) afforded **15** (0.313 g, 2.085 mmol, yield 48%).
^{1}H-NMR (MeOD-*d4*) δ_H, ppm: 7.69 (1H, d, NH-(C=O)-CH-C$\underline{H}$, $^3J_{H,H}$ = 7.9 Hz), 5.71 (1H, d, NH-(C=O)-C$\underline{H}$-CH, $^3J_{H,H}$ = 7.9 Hz), 4.57 (2H, d, NH-(C=O)-N-C$\underline{H}_2$-C≡CH, $^4J_{H,H}$ = 2.5 Hz), 2.91 (1H, t, NH-(C=O)-N-CH_2-C≡C$\underline{H}$, $^4J_{H,H}$ = 2.5 Hz).
^{13}C-NMR (MeOD-*d4*) δ_C, ppm: 166.5 (1C, NH-($\underline{C}$=O)-CH-CH), 152.2 (1C, NH-($\underline{C}$=O)-N-CH_2), 145.7 (1C, NH-(C=O)-CH-$\underline{C}$H), 102.9 (1C, NH-(C=O)-$\underline{C}$H-CH), 77.9 (1C, NH-(C=O)-N-CH_2-$\underline{C}$≡CH), 75.6 (1C, NH-(C=O)-N-CH_2-C≡$\underline{C}$H), 37.9 (1C, NH-(C=O)-N-$\underline{C}H_2$-C≡CH).
R_f (CH_2Cl_2/CH_3OH 10:1) = 0.496.
ESI-MS(+): *m/z* found 151.0502 [M+H$^+$]$^+$, 173.0321 [M+Na$^+$]$^+$, 323.0753 [2M+Na$^+$]$^+$, 473.1179 [3M+Na$^+$]$^+$, calcd. for $C_7H_7N_2O_2^+$ 151.0502, $C_7H_6N_2NaO_2^+$ 173.0321, $C_{14}H_{12}N_4NaO_4^+$ 323.0751, $C_{21}H_{18}N_6NaO_6^+$ 473.1180.
Elemental analysis (%): calcd. for $C_7H_6N_2O_2$ C 56.00, H 4.03, N 18.66; found C 56.05, H 3.96, N 18.66.

Synthesis of 1-propargyl-thymine (5-methyl-1-(prop-2-yn-1-yl)pyrimidine-2,4(1*H*,3*H*)-dione) (16)
To a solution of thymine (2,4-dihydroxy-5-methylpyrimidine) (0.510 g, 3.960 mmol, 1 equiv.) in dry DMF (20 mL) at 60°C under inert atmosphere (N_2) was added K_2CO_3 (0.274 g, 1.980 mmol, 0.5 equiv.) followed by dropwise addition of propargyl bromide (0.43 mL, 3.960 mmol, 1 equiv.). The mixture was stirred at 60°C for 48 h and the reaction evolution was verified by TLC. The reaction mixture was allowed to cool at r.t. and was concentrated under reduced pressure. Purification by column chromatography (CH_2Cl_2/CH_3OH 9:1 (v/v)) afforded **16** (0.280 g, 1.705 mmol, yield 43%).
^{1}H-NMR (MeOD-*d4*) δ_H, ppm: 7.51 (1H, m, NH-(C=O)-N-C$\underline{H}$, $^4J_{H,H}$ = 1.2 Hz), 4.54 (2H, d, NH-(C=O)-N-C$\underline{H}_2$-C≡CH, $^4J_{H,H}$ = 2.5 Hz), 2.88 (1H, t, NH-(C=O)-N-CH_2-C≡C$\underline{H}$, $^4J_{H,H}$ = 2.5 Hz), 1.88 (3H, d, N-CH-C-C$\underline{H}_3$, $^4J_{H,H}$ = 1.1 Hz).
^{13}C-NMR (MeOD-*d4*) δ_C, ppm: 166.6 (1C, ($\underline{C}$=O)-NH-(C=O)-N-CH_2), 152.3 (1C, (C=O)-NH-($\underline{C}$=O)-N-CH_2), 141.5 (1C, (C=O)-N-$\underline{C}$H-C-CH_3), 111.9 (1C, (C=O)-N-CH-$\underline{C}$-CH_3), 78.2 (1C, NH-(C=O)-N-CH_2-$\underline{C}$≡CH), 75.3 (1C, NH-(C=O)-N-CH_2-C≡$\underline{C}$H), 37.6 (1C, NH-(C=O)-N-$\underline{C}H_2$-C≡CH), 12.2 (1C, (C=O)-N-CH-C-$\underline{C}H_3$).
R_f (CH_2Cl_2/CH_3OH 10:1) = 0.600.

ESI-MS(+): *m/z* found 165.0659 $[M+H]^+$, 187.0478 $[M+Na]^+$, calcd. for $C_8H_9N_2O_2^+$ 165.0659, $C_8H_8N_2NaO_2^+$ 187.0478.

Elemental analysis (%): calcd. for $C_8H_8N_2O_2$ C 58.53, H 4.91, N 17.06; found C 58.56, H 4.93, N 17.04.

Synthesis of 1-propargyl-cytosine (4-amino-1-(prop-2-yn-1-yl)pyrimidin-2(1*H*)-one) (17)

To a solution of cytosine (4-aminopyrimidin-2(1*H*)-one) (0.600 g, 5.400 mmol, 1 equiv.) in dry DMF at 60°C (20 mL), under inert atmosphere (N_2) was added K_2CO_3 (0.597 g, 4.319 mmol, 0.5 equiv.), followed by dropwise addition of propargyl bromide (0.56 mL, 5.396 mmol, 1 equiv.). The mixture was stirred at 60°C for 48 h and the reaction evolution was verified by TLC. The reaction mixture was allowed to cool to r.t. and was concentrated under reduced pressure. Purification by column chromatography (CH_2Cl_2/CH_3OH 10:1 (v/v)) afforded **17** (0.206 g, 1.389 mmol, yield 25%).

^{1}H-NMR (MeOD-*d$_4$*) δ_H, ppm: 7.50 (2H, s br, NH_2), 7.49 (1H, d, N-C*H̲*-CH-C-NH_2, $^3J_{H,H}$ = 7.7 Hz), 6.58 (1H, d, N-CH-C*H̲*-C-NH_2, $^3J_{H,H}$ = 7.8 Hz), 4.80 (2H, d, N-(C=O)-N-C*H̲*$_2$-C≡CH, $^4J_{H,H}$ = 2.5 Hz), 2.95 (1H, t, N-(C=O)-N-CH_2-C≡C*H̲*, $^4J_{H,H}$ = 2.5 Hz).

^{13}C-NMR (MeOD-*d$_4$*) δ_C, ppm: 146.6 (1C, N-(C=O)-N-*C̲*-NH_2), 143.0 (1C, N-(*C̲*=O)-N-CH_2), 133.4 (1C, N-*C̲*H-CH-C-NH_2), 98.8 (1C, N-CH-*C̲*H-C-NH_2), 78.0 (1C, N-(C=O)-N-CH_2-*C̲*≡CH), 75.9 (1C, N-(C=O)-N-CH_2-C≡*C̲*H), 38.9 (1C, N-(C=O)-N-*C̲*H_2-C≡CH).

R_f (CH_2Cl_2/CH_3OH 10:1) = 0.465.

ESI-MS(+): *m/z* found 150.0659 $[M+H]^+$, 172.0479 $[M+Na]^+$, 188.0816 $[M+K]^+$, calcd. for $C_7H_8N_3O^+$ 150.0662, $C_7H_7N_3NaO^+$ 172.0481, $C_7H_7KN_3O^+$ 188.0221.

Elemental analysis (%): calcd. for $C_7H_7N_3O$ C 56.37, H 4.73, N 28.17; found C 61.77, H 4.91, N 21.76.

Synthesis of 9-propargyl-adenine (9-(prop-2-yn-1-yl)-9*H*-purin-6-amine) (18)

To a solution of adenine (9*H*-purin-6-amine) (0.600 g, 4.440 mmol, 1 equiv.) in dry DMF (20 mL) at 60°C under inert atmosphere (N_2) was added K_2CO_3 (0.306 g, 2.214 mmol, 0.5 equiv.), followed by dropwise addition of propargyl bromide (0.46 mL, 4.438 mmol, 1 equiv.). The mixture was stirred at 60°C for 48 h and the reaction evolution was verified by TLC. The reaction mixture was allowed to cool to r.t. and was concentrated under reduced pressure. Purification by column chromatography (CH_2Cl_2/CH_3OH 10:1 (v/v)) afforded **18** (0.238 g, 1.374 mmol, yield 31%).

^{1}H-NMR (MeOD-*d$_4$*) δ_H, ppm: 8.22 (1H, s, N-C*H̲*-N-C-NH_2), 8.21 (1H, s, N-C*H̲*-N-C-C-NH_2), 5.06 (2H, d, N-C*H̲*$_2$-C≡CH, $^4J_{H,H}$ = 2.4 Hz), 2.98 (1H, t, N-CH_2-C≡C*H̲*, $^4J_{H,H}$ = 2.5 Hz).

^{13}C-NMR (MeOD-*d$_4$*) δ_C, ppm: 157.4 (1C, N-CH-N-C-*C̲*-NH_2), 153.9 (1C, N-*C̲*H-N-C-NH_2), 150.3 (1C, N-*C̲*-N-CH_2-C≡CH), 141.9 (1C, N-*C̲*H-N-C-C-NH_2), 119.9 (1C, N-CH-N-*C̲*-C-NH_2), 77.6 (1C, N-CH_2-*C̲*≡CH), 75.7 (1C, N-CH_2-C≡*C̲*H), 33.8 (1C, N-C-N-*C̲*H_2-C≡CH).

ESI-MS(+): *m/z* found 174.0773 $[M+H]^+$, 196.0593 $[M+Na]^+$, calcd. for $C_8H_8N_5^+$ 174.0774, $C_8H_7N_5Na^+$ 196.0594.

Elemental analysis (%): calcd. for $C_8H_8N_5$ C 55.16, H 4.63, N 40.21, found C 55.30, H 4.18, N 40.18.

Compounds **19-26**, **28-31**, and **33-39** were prepared and purified by adapting a literature protocols [11,12].

Synthesis of [(η^6-*p*-MeC$_6$H$_4$Pr^i)$_2$Ru$_2$(μ_2-SCH$_2$C$_6$H$_4$-*p*-Bu^t)$_2$(μ_2-SC$_6$H$_4$-*o*-R)]Cl (R = ((1-methylene-1*H*-1,2,3-triazol-4-yl)methyl)-1-pyrimidine-2,4(1*H*,3*H*)-dione) (19)

To a solution of **9** **(EP3-141)** (0.250 g, 0.242 mmol 1 equiv.) in dry DMF (10 mL) under inert atmosphere (N_2) at r.t were added successively **15** (0.043 g, 0.288 mmol, 1.2 equiv.), $Cu_2SO_4 \cdot 5H_2O$ (0.060 g, 0.240 mmol, 1 equiv.) and sodium ascorbate (0.096 g, 0.484 mmol, 2 equiv.). The mixture was

stirred at 50°C overnight and the reaction evolution was verified by TLC. The reaction mixture was cooled to r.t., filtered and the filtrate was diluted with EtOAc (100 mL). The organic phase was successively washed with H_2O (2x100 mL), saturated aq. soln. NH_4Cl, dried over anhydrous Na_2SO_4, filtered and concentrated under reduced pressure. Purification by column chromatography (CH_2Cl_2/CH_3OH 9:1 (v/v)) afforded **19** as an orange solid (0.063 g, 0.054 mmol, yield 22%).

^{1}H-NMR ($CDCl_3$) δ_H, ppm: 9.07 (1H, s, (*Tr*)N-N=N-C-C*H*), 8.76 (1H, s, CH$_2$-N-(C=O)-N*H*-(C=O), 7.76 (1H, d, S-(*Ar*)C-C*H*-CH-CH-CH, $^3J_{H,H}$ = 7.7 Hz), 7.71 (1H, d, CH$_2$-N-CH-CH-(C=O), $^3J_{H,H}$ = 7.8 Hz), 7.57 (2H, d, S-CH$_2$-(*Ar*)C-CH-C*H*-C-C(CH$_3$)$_3$, $^3J_{H,H}$ = 8.1 Hz), 7.49 (2H, d, S-CH$_2$-(*Ar*)C-C*H*-CH-C-C(CH$_3$)$_3$, $^3J_{H,H}$ = 8.1 Hz), 7.40-7.43 (4H, m, S-CH$_2$-(*Ar*)C-CH-C*H*-C-C(CH$_3$)$_3$, S-CH$_2$-(*Ar*)C-C*H*-CH-C-C(CH$_3$)$_3$, $^3J_{H,H}$ = 8.6 Hz), 7.26-7.32 (2H, m, S-(*Ar*)C-CH-CH-C*H*-CH, S-(*Ar*)C-CH-CH-CH-C*H*), 7.21-7.26 (1H, m, S-(*Ar*)C-CH-C*H*-CH-CH), 6.36 (2H, s br, S-(*Ar*)C-C-C*H*$_2$-(*Tr*)N-N=N), 5.63 (1H, d, CH$_2$-N-CH-C*H*-(C=O), $^3J_{H,H}$ = 7.0 Hz), 5.13 (2H, s br, (*Tr*)N-N=N-C-C*H*$_2$-N), 5.06 (2H, d, 2xCH$_3$-(*Ar*)C-CH-C*H*-C, $^3J_{H,H}$ = 5.6 Hz), 5.00 (2H, d, 2xCH$_3$-(*Ar*)C-CH-C*H*-C, $^3J_{H,H}$ = 5.8 Hz), 4.94 (2H, d, 2xCH$_3$-(*Ar*)C-C*H*-CH-C, $^3J_{H,H}$ = 5.7 Hz), 4.66 (2H, d, 2xCH$_3$-(*Ar*)C-C*H*-CH-C, $^3J_{H,H}$ = 5.8 Hz), 3.65 (2H, s, S-C*H*$_2$-(*Ar*)C-CH-CH-C-C(CH$_3$)$_3$), 3.36 (2H, s, S-C*H*$_2$-(*Ar*)C-CH-CH-C-C(CH$_3$)$_3$), 1.87 (2H, sept, 2x(*Ar*)C-CH-CH-C-C*H*(CH$_3$)$_2$, $^3J_{H,H}$ = 6.9 Hz), 1.59 (6H, (6H, s, 2xC*H*$_3$-(*Ar*)C-CH-CH-C), 1.35 (9H, s, S-CH$_2$-(*Ar*)C-CH-CH-C-C(C*H*$_3$)$_3$), 1.33 (9H, s, S-CH$_2$-(*Ar*)C-CH-CH-C-C(C*H*$_3$)$_3$), 0.92 (6H, d, (*Ar*)C-CH-CH-C-CH(C*H*$_3$)$_2$, $^3J_{H,H}$ = 7.0 Hz), 0.90 (6H, d, (*Ar*)C-CH-CH-C-CH(C*H*$_3$)$_2$, $^3J_{H,H}$ = 7.0 Hz).

^{13}C-NMR ($CDCl_3$) δ_C, ppm: 163.5 (1C, CH$_2$-N-CH-CH-(C=O)-NH), 151.9, 151.6 (2C, 2xS-CH$_2$-(*Ar*)C-CH-CH-C-C(CH$_3$)$_3$), 150.9 (1C, CH$_2$-N-(C=O)-NH-CH-CH), 145.1 (1C, CH$_2$-N-CH-CH-(C=O)), 141.9 (1C, (*Tr*)N-N=N-C-CH), 139.2 (1C, S-(*Ar*)C-C-CH-CH-CH-CH), 137.9 (1C, S-(*Ar*)C-C-CH-CH-CH-CH), 136.8, 136.6 (2C, 2xS-CH$_2$-(*Ar*)C-CH-CH-C-C(CH$_3$)$_3$), 133.8 (1C, S-(*Ar*)C-CH-CH-CH-CH), 130.6 (1C, S-(*Ar*)C-CH-CH-CH-CH), 129.8 (2C, 2xS-CH$_2$-(*Ar*)C-CH-CH-C-C(CH$_3$)$_3$), 129.2 (1C, S-(*Ar*)C-CH-CH-CH-CH), 129.1 (1C, S-(*Ar*)C-CH-CH-CH-CH), 129.0 (2C, 2xS-CH$_2$-(*Ar*)C-CH-CH-C-C(CH$_3$)$_3$), 126.8 (1C, (Tr)N-N=N-C-CH), 125.8, 125.6 (4C, 2xS-CH$_2$-(*Ar*)C-CH-CH-C-C(CH$_3$)$_3$), 106.8 (2C, 2xCH$_3$-(*Ar*)C-CH-CH-C), 102.5 (1C, CH$_2$-N-CH-CH-(C=O)-NH), 101.2 (2C, 2xCH$_3$-(*Ar*)C-CH-CH-C), 83.6 (2C, CH$_3$-(*Ar*)C-CH-CH-C), 83.5 (4C, CH$_3$-(*Ar*)C-CH-CH-C, CH$_3$-(*Ar*)C-CH-CH-C), 82.8 (2C, CH$_3$-(*Ar*)C-CH-CH-C), 52.9 (1C, S-(*Ar*)-C-C-CH$_2$-(*Tr*)N-N=N), 42.8 (1C, S-(*Ar*)C-C-CH$_2$-(*Tr*)N-N=N-C-CH$_2$-N), 40.3 (1C, S-CH$_2$-(*Ar*)C-CH-CH-C-C(CH$_3$)$_3$), 39.5 (1C, S-CH$_2$-(*Ar*)C-CH-CH-C-C(CH$_3$)$_3$), 34.9 (2C, 2xS-CH$_2$-(*Ar*)C-CH-CH-C-C(CH$_3$)$_3$), 31.57, 31.52 (6C, 2xS-CH$_2$-(*Ar*)C-CH-CH-C-C(CH$_3$)$_3$), 30.9 (2C, 2x(*Ar*)CH-CH-C-CH(CH$_3$)$_2$), 23.1 (2C, (*Ar*)CH-CH-C-CH(CH$_3$)$_2$), 22.7 (2C, (*Ar*)CH-CH-C-CH(CH$_3$)$_2$), 18.1 (2C, 2xCH$_3$-(*Ar*)C-CH-CH-C).

R_f (CH_2Cl_2/CH_3OH 10:1) = 0.212.

ESI-MS(+): *m/z* found 1144.2764 [M-Cl]$^+$, calcd. for $C_{56}H_{70}N_5O_2Ru_2S_3^+$ 1144.2733.

Elemental analysis (%): calcd. for $C_{56}H_{70}ClN_5O_2Ru_2S_3 \cdot 0.75CH_2Cl_2$ C 54.85, H 5.80, N 5.64; found C 54.81, H 5.81, N 5.69.

Synthesis of [(η^6-*p*-MeC$_6$H$_4$Pr^i)$_2$Ru$_2$(μ_2-SCH$_2$C$_6$H$_4$-*p*-Bu^t)$_2$(μ_2-SC$_6$H$_4$-*o*-R)]Cl (R = 1-((1-methylene-1*H*-1,2,3-triazol-4-yl)methyl)-5-methylpyrimidine-2,4(1*H*,3*H*)-dione) (20)

To a solution of **9** (0.250 g, 0.242 mmol 1 equiv.) in dry DMF (10 mL) under inert atmosphere (N_2) at r.t were added successively **16** (0.047 g, 0.288 mmol, 1.2 equiv.), $Cu_2SO_4 \cdot 5H_2O$ (0.060 g, 0.240 mmol, 1 equiv.) and sodium ascorbate (0.096 g, 0.484 mmol, 2 equiv.). The mixture was stirred at 50°C for 24 h and the reaction evolution was verified by TLC. The reaction mixture was cooled to r.t., filtered and the filtrate was diluted with EtOAc (100 mL). The organic phase was washed successively with H_2O (2x100 mL), saturated aq. soln. NH_4Cl, dried over anhydrous Na_2SO_4, filtered and concentrated under

reduced pressure. Purification by column chromatography (CH_2Cl_2/CH_3OH 9.5:1 (v/v)) afforded **20** as an orange solid (0.055 g, 0.046 mmol, yield 19%).

^{1}H-NMR ($CDCl_3$) δ_H, ppm: 9.10 (1H, s, (*Tr*)N-N=N-C-C*H*), 8.59 (1H, s, CH_2-N-(C=O)-N*H*-(C=O), 7.75 (1H, d, S-(*Ar*)C-C*H*-CH-CH-CH, $^3J_{H,H}$ = 7.7 Hz), 7.57 (2H, d, 2xS-CH_2-(*Ar*)C-CH-C*H*-C-C(CH_3)$_3$, $^3J_{H,H}$ = 8.2 Hz), 7.48 (2H, d, 2xS-CH_2-(*Ar*)C-C*H*-CH-C-C(CH_3)$_3$, $^3J_{H,H}$ = 8.2 Hz), 7.44 (1H, s, CH_2-N-C*H*-C-CH_3), 7.38-7.44 (4H, m, 2xS-CH_2-(*Ar*)C-C*H*-CH-C-C(CH_3)$_3$, 2xS-CH_2-(*Ar*)C-CH-C*H*-C-C(CH_3)$_3$, $^3J_{H,H}$ = 8.6 Hz), 7.27-7.29 (2H, m, S-(*Ar*)C-CH-CH-C*H*-CH, S-(*Ar*)C-CH-CH-CH-C*H*), 7.20-7.24 (1H, m, S-(*Ar*)C-CH-C*H*-CH-CH), 6.40 (2H, s br, S-(*Ar*)C-C-C*H$_2$*-(*Tr*)N-N=N), 5.08 (2H, s br, (*Tr*)N-N=N-C-C*H$_2$*-N), 5.06 (2H, d, 2xCH_3-(*Ar*)C-CH-C*H*-C, $^3J_{H,H}$ = 5.8 Hz), 5.03 (2H, d, 2xCH_3-(*Ar*)C-CH-C*H*-C, $^3J_{H,H}$ = 5.9 Hz), 4.95 (2H, d, 2xCH_3-(*Ar*)C-C*H*-CH-C, $^3J_{H,H}$ = 5.7 Hz), 4.67 (2H, d, 2xCH_3-(*Ar*)C-C*H*-CH-C, $^3J_{H,H}$ = 5.8 Hz), 3.63 (2H, s, S-C*H$_2$*-(*Ar*)C-CH-CH-C-C(CH_3)$_3$), 3.36 (2H, s, S-C*H$_2$*-(*Ar*)C-CH-CH-C-C(CH_3)$_3$), 1.86 (2H, sept, 2x(*Ar*)C-CH-CH-C-C*H*(CH_3)$_2$), $^3J_{H,H}$ = 6.8 Hz), 1.83 (3H, s, CH_2-N-CH-C-C*H$_3$*), 1.58 (6H, s, 2xC*H$_3$*-(*Ar*)C-CH-CH-C), 1.35 (9H, s, S-CH_2-(*Ar*)C-CH-CH-C-C(C*H$_3$*)$_3$), 1.33 (9H, s, S-CH_2-(*Ar*)C-CH-CH-C-C(C*H$_3$*)$_3$), 0.90 (6H, d, (*Ar*)C-CH-CH-C-CH(C*H$_3$*)$_2$, $^3J_{H,H}$ = 6.8 Hz), 0.88 (6H, d, (*Ar*)C-CH-CH-C-CH(C*H$_3$*)$_2$, $^3J_{H,H}$ = 6.8 Hz).

^{13}C-NMR ($CDCl_3$) δ_C, ppm: 164.1 (1C, NH-(*C*=O)-C-CH_3), 151.9, 151.6 (2C, 2xS-CH_2-(*Ar*)C-CH-CH-*C*-C(CH_3)$_3$), 150.9 (1C, CH_2-N-(*C*=O)-NH-(C=O)), 141.5 (1C, (*Tr*)N-N=N-*C*-CH), 140.7 (1C, CH_2-N-*C*H-C-CH_3), 139.3 (1C, S-(*Ar*)*C*-C-CH-CH-CH-CH), 137.9 (1C, S-(*Ar*)C-*C*-CH-CH-CH-CH), 136.8, 136.6 (2C, 2xS-CH_2-(*Ar*)*C*-CH-CH-C-C(CH_3)$_3$), 133.9 (1C, S-(*Ar*)C-*C*H-CH-CH-CH), 130.7 (1C, S-(*Ar*)C-CH-CH-CH-*C*H), 129.8 (2C, 2xS-CH_2-(*Ar*)C-CH-*C*H-C-C(CH_3)$_3$), 129.1 (1C, S-(*Ar*)C-CH-CH-*C*H-CH), 129.0 (3C, 2xS-CH_2-(*Ar*)C-CH-*C*H-C-C(CH_3)$_3$, S-(*Ar*)C-CH-*C*H-CH-CH), 126.6 (1C, (*Tr*)N-N=N-C-*C*H), 125.8, 125.5 (4C, 4xS-CH_2-(*Ar*)C-*C*H-CH-C-C(CH_3)$_3$), 111.0 (1C, CH_2-N-CH-*C*-CH_3), 106.7 (2C, 2xCH_3-(*Ar*)C-CH-CH-*C*), 101.2 (2C, 2xCH_3-(*Ar*)*C*-CH-CH-C), 83.6 (2C, 2xCH_3-(*Ar*)C-CH-*C*H-C), 83.5 (4C, 2xCH_3-(*Ar*)C-*C*H-CH-C, 2xCH_3-(*Ar*)C-CH-*C*H-C), 82.8 (2C, 2xCH_3-(*Ar*)C-*C*H-CH-C), 53.0 (1C, S-(*Ar*)-C-C-*C*H_2-(*Tr*)N-N=N), 42.7 (1C, S-(*Ar*)C-C-CH_2-(*Tr*)N-N=N-C-*C*H_2), 40.4 (1C, S-*C*H_2-(*Ar*)C-CH-CH-C-C(CH_3)$_3$), 39.5 (1C, S-*C*H_2-(*Ar*)C-CH-CH-C-C(CH_3)$_3$), 34.9 (2C, 2xS-CH_2-(*Ar*)C-CH-CH-C-*C*(CH_3)$_3$), 31.57, 31.51 (6C, 2xS-CH_2-(*Ar*)C-CH-CH-C-C(*C*H_3)$_3$), 30.9 (2C, 2x(*Ar*)CH-CH-C-*C*H(CH_3)$_2$), 23.2 (2C, (*Ar*)CH-CH-C-CH(*C*H_3)$_2$), 22.7 (2C, (*Ar*)CH-CH-C-CH(*C*H_3)$_2$), 18.1 (2C, 2x*C*H_3-(*Ar*)C-CH-CH-C), 12.4 (1C, CH_2-N-CH-C-*C*H_3).

R_f (CH_2Cl_2/CH_3OH 10:1) = 0.121.

ESI-MS(+): *m/z* found 1158.2900, calcd. for $C_{57}H_{72}N_5O_2Ru_2S_3^+$ 1158.2930.

Elemental analysis (%): calcd. for $C_{57}H_{72}N_5O_2Ru_2S_3Cl\cdot1.25CH_3OH$ C 56.74, H 6.29, N 5.68; found C 56.70, H 6.24, N 5.26.

Synthesis of [(η^6-*p*-MeC$_6$H$_4$Pr^i)$_2$Ru$_2$(μ_2-SCH$_2$C$_6$H$_4$-*p*-Bu^t)$_2$(μ_2-SC$_6$H$_4$-*o*-R)]Cl (R = 4-amino-1-((1-methylene-1*H*-1,2,3-triazol-4-yl)methyl)pyrimidin-2(1*H*)-one) (21)

To a solution of **9** (0.250 g, 0.242 mmol 1 equiv.) in dry DMF (10 mL) under inert atmosphere (N_2) at r.t were added successively **17** (0.043 g, 0.288 mmol, 1.2 equiv.), $Cu_2SO_4\cdot5H_2O$ (0.060 g, 0.240 mmol, 1 equiv.) and sodium ascorbate (0.096 g, 0.484 mmol, 2 equiv.). The mixture was stirred at 50°C overnight and the reaction evolution was verified by TLC. The reaction mixture was cooled to r.t., filtered and the filtrate was diluted with EtOAc (100 mL). The organic phase was successively washed with H_2O (2x100 mL), saturated aq. soln. NH_4Cl (100 mL), dried over anhydrous Na_2SO_4, filtered and concentrated under reduced pressure. Purification by column chromatography (CH_2Cl_2/CH_3OH 9:1 (v/v)) afforded **21** as an orange solid (0.137 g, 0.116 mmol, yield 48%).

^{1}H-NMR ($CDCl_3$) δ_H, ppm: 9.08 (1H, s, (*Tr*)N-N=N-C-C*H*), 7.73 (1H, d, S-(*Ar*)C-C*H*-CH-CH-CH, $^3J_{H,H}$ = 7.6 Hz), 7.57 (2H, d, 2xS-CH_2-(*Ar*)C-CH-C*H*-C-C(CH_3)$_3$, $^3J_{H,H}$ = 8.2 Hz), 7.49 (2H, d, 2xS-CH_2-

(Ar)C-C$\underline{H}$-CH-C-C(CH_3)$_3$, $^3J_{H,H}$ = 8.2 Hz), 7.45 (1H, d, CH_2-N-C$\underline{H}$-CH-C-NH_2, $^3J_{H,H}$ = 7.8 Hz), 7.37-7.43 (4H, m, 2xS-CH_2-(Ar)C-CH-C$\underline{H}$-C-C(CH_3)$_3$, 2xS-CH_2-(Ar)C-C$\underline{H}$-CH-C-C(CH_3)$_3$, $^3J_{H,H}$ = 8.5 Hz), 7.26-7.32 (2H, m, S-(Ar)C-CH-CH-C$\underline{H}$-CH, S-(Ar)C-CH-CH-CH-C$\underline{H}$), 7.19-7.24 (1H, td, S-(Ar)C-CH-C$\underline{H}$-CH-CH-C, $^3J_{H,H}$ = 6.7 Hz, $^4J_{H,H}$ = 1.9 Hz), 6.47 (1H, d br, CH_2-N-CH-C$\underline{H}$-C-NH_2, $^3J_{H,H}$ = 7.0 Hz), 6.39 (2H, s br, S-(Ar)C-C-C$\underline{H_2}$-(Tr)N-N=N), 5.32 (2H, s, (Tr)N-N=N-C-C$\underline{H_2}$-N), 5.01 (2H, d, 2xCH_3-(Ar)C-CH-C$\underline{H}$-C, $^3J_{H,H}$ = 6.0 Hz), 4.99 (2H, d, 2xCH_3-(Ar)C-CH-C$\underline{H}$-C, $^3J_{H,H}$ = 6.3 Hz), 4.88 (2H, d, 2xCH_3-(Ar)C-C$\underline{H}$-CH-C, $^3J_{H,H}$ = 5.7 Hz), 4.64 (2H, d, 2xCH_3-(Ar)C-C$\underline{H}$-CH-C, $^3J_{H,H}$ = 5.8 Hz), 3.58 (2H, s, S-C$\underline{H_2}$-(Ar)C-CH-CH-C-C(CH_3)$_3$), 3.35 (2H, s, S-C$\underline{H_2}$-(Ar)C-CH-CH-C-C(CH_3)$_3$), 1.84 (2H, sept, 2x(Ar)C-CH-CH-C-C$\underline{H}$(CH_3)$_2$, $^3J_{H,H}$ = 7.0 Hz), 1.54 (6H, s, 2xC$\underline{H_3}$-(Ar)C-CH-CH-C), 1.35 (9H, s, S-CH_2-(Ar)C-CH-CH-C-C(C$\underline{H_3}$)$_3$), 1.32 (9H, s, S-CH_2-(Ar)C-CH-CH-C-C(C$\underline{H_3}$)$_3$), 0.89 (6H, d, (Ar)C-CH-CH-C-CH(C$\underline{H_3}$)$_2$, $^3J_{H,H}$ = 7.1 Hz), 0.87 (6H, d, (Ar)C-CH-CH-C-CH(C$\underline{H_3}$)$_2$, $^3J_{H,H}$ = 7.1 Hz).
^{13}C-NMR ($CDCl_3$) δ_C, ppm: 151.8, 151.5 (2C, 2xS-CH_2-(Ar)C-CH-CH-$\underline{C}$-C(CH_3)$_3$), 146.2 (1C, CH_2-N-($\underline{C}$=O)-N-C-NH_2), 144.9 (1C, CH_2-N-(C=O)-N-$\underline{C}$-NH_2), 141.7 (1C, (Tr)N-N=N-$\underline{C}$-CH), 139.2 (1C, S-(Ar)$\underline{C}$-C-CH-CH-CH-CH), 138.0 (1C, S-(Ar)C-$\underline{C}$-CH-CH-CH-CH), 136.8, 136.5 (2C, 2xS-CH_2-(Ar)$\underline{C}$-CH-CH-C-C(CH_3)$_3$), 133.9 (1C, S-(Ar)C-$\underline{C}$H-CH-CH-CH), 132.2 (1C, CH_2-N-$\underline{C}$H-CH-C-NH_2), 130.8 (1C, S-(Ar)C-CH-CH-CH-$\underline{C}$H), 129.9 (2C, 2xS-CH_2-(Ar)C-CH-$\underline{C}$H-C-C(CH_3)$_3$), 129.2 (1C, S-(Ar)C-CH-CH-$\underline{C}$H-CH), 129.04 (1C, S-(Ar)C-CH-$\underline{C}$H-CH-CH), 129.01 (2C, 2xS-CH_2-(Ar)C-CH-$\underline{C}$H-C-C(CH_3)$_3$), 126.4 (1C, (Tr)N-N=N-C-$\underline{C}$H), 125.7, 125.5 (4C, 4xS-CH_2-(Ar)C-$\underline{C}$H-CH-C-C(CH_3)$_3$), 106.8 (2C, 2xCH_3-(Ar)C-CH-CH-$\underline{C}$), 101.1 (2C, 2xCH_3-(Ar)$\underline{C}$-CH-CH-C), 98.8 (1C, CH_2-N-CH-$\underline{C}$H-C-NH_2), 83.6 (2C, 2xCH_3-(Ar)C-CH-$\underline{C}$H-C), 83.5 (2C, 2xCH_3-(Ar)C-$\underline{C}$H-CH-C), 83.3 (2C, 2xCH_3-(Ar)C-CH-$\underline{C}$H-C), 82.7 (2C, 2xCH_3-(Ar)C-$\underline{C}$H-CH-C), 53.1 (1C, S-(Ar)-C-C-$\underline{C}H_2$-(Tr)N-N=N), 43.8 (1C, (Tr)N-N=N-C-$\underline{C}H_2$), 40.4 (1C, S-$\underline{C}H_2$-(Ar)C-CH-CH-C-C(CH_3)$_3$), 39.5 (1C, S-$\underline{C}H_2$-(Ar)C-CH-CH-C-C(CH_3)$_3$), 34.87, 34.86 (2C, S-CH_2-(Ar)C-CH-CH-C-$\underline{C}$(CH_3)$_3$), 31.6, 31.5 (6C, 2xS-CH_2-(Ar)C-CH-CH-C-C($\underline{C}H_3$)$_3$), 30.9 (2C, 2x(Ar)CH-CH-C-$\underline{C}$H(CH_3)$_2$), 23.1 (2C, (Ar)CH-CH-C-CH($\underline{C}H_3$)$_2$), 22.7 (2C, (Ar)CH-CH-C-CH($\underline{C}H_3$)$_2$), 18.1 (2C, 2x$\underline{C}H_3$-(Ar)C-CH-CH).
R_f (CH_2Cl_2/CH_3OH 10:1) = 0.231.
ESI-MS(+): m/z found 1181.3091 [M-Cl-H+K]$^+$, calcd. for $C_{56}H_{70}KN_6ORu_2S_3^+$ 1181.2492.
Elemental analysis (%): calcd. for $C_{56}H_{71}N_6ORu_2S_3Cl\cdot0.8CH_3OH$ C 56.68, H 6.21, N 6.98; found C 56.67, H 6.22, N 6.69.

Synthesis of [(η^6-p-$MeC_6H_4Pr^i$)$_2Ru_2$(μ_2-$SCH_2C_6H_4$-p-Bu^t)$_2$(μ_2-SC_6H_4-o-R)]Cl (R = 9-((1-methylene-1H-1,2,3-triazol-4-yl)methyl)-9H-purin-6-amine) (22)

To a solution of **9** (0.250 g, 0.242 mmol, 1 equiv.) in dry DMF (10 mL) under inert atmosphere (N_2) at r.t were added successively **18** (0.050 g, 0.288 mmol, 1.2 equiv.), $Cu_2SO_4\cdot5H_2O$ (0.060 g, 0.240 mmol, 1 equiv.) and sodium ascorbate (0.096 g, 0.484 mmol, 2 equiv.). The mixture was stirred at 50°C overnight and the reaction evolution was verified by TLC. The reaction mixture was cooled to r.t., filtered and diluted with EtOAc (100 mL), the organic phase was washed with H_2O (2x100 mL) and saturated aq. soln. NH_4Cl (100 mL). The organic phase was dried over anhydrous Na_2SO_4, filtered and concentrated under reduced pressure. Purification by column chromatography (CH_2Cl_2/CH_3OH 8:2 (v/v)) afforded **22** as an orange solid (0.116 g, 0.096 mmol, yield 40%).
^{1}H-NMR (DMSO-d_6) δ_H, ppm: 8.30 (1H, s, (Tr)N-N=N-C-C$\underline{H}$), 8.28 (1H, s, N-C$\underline{H}$-N-C-NH_2), 8.04 (1H, s, N-C$\underline{H}$-N-C-C-NH_2), 7.72 (1H, d, S-(Ar)C-C$\underline{H}$-CH-CH-CH, $^3J_{H,H}$ = 7.8 Hz), 7.49-7.55 (4H, m, 2xS-CH_2-(Ar)C-CH-C$\underline{H}$-C-C(CH_3)$_3$, 2xS-CH_2-(Ar)C-C$\underline{H}$-CH-C-C(CH_3)$_3$), 7.40-7.47 (4H, m, 2xS-CH_2-(Ar)C-C$\underline{H}$-CH-C-C(CH_3)$_3$, 2xS-CH_2-(Ar)C-CH-C$\underline{H}$-C-C(CH_3)$_3$, $^3J_{H,H}$ = 8.4 Hz), 7.36 (1H, d, S-(Ar)C-CH-CH-CH-C$\underline{H}$-C, $^3J_{H,H}$ = 7.3 Hz), 7.27-7.34 (2H, m, S-(Ar)C-CH-C$\underline{H}$-CH-CH-C, S-(Ar)C-CH-CH-C$\underline{H}$-CH-C, $^3J_{H,H}$ = 7.9 Hz), 7.16 (1C, s br, N-CH-N-C-C-N$\underline{H_2}$), 6.13 (2H, s br, S-(Ar)C-C-C$\underline{H_2}$-(Tr)N-

N=N), 5.54 (2H, s br, (*Tr*)N-N=N-C-C*H*$_2$-N), 5.17 (2H, d, 2xCH$_3$-(*Ar*)C-CH-C*H*-C, $^3J_{H,H}$ = 5.8 Hz), 5.07 (2H, d, 2xCH$_3$-(*Ar*)C-C*H*-CH-C, $^3J_{H,H}$ = 5.8 Hz), 4.95 (2H, d, 2xCH$_3$-(*Ar*)C-CH-C*H*-C, $^3J_{H,H}$ = 5.9 Hz), 4.60 (2H, d, 2xCH$_3$-(*Ar*)C-C*H*-CH-C, $^3J_{H,H}$ = 5.9 Hz), 3.62 (2H, s, S-C*H*$_2$-(*Ar*)C-CH-CH-C-C(CH$_3$)$_3$), 3.43 (2H, s, S-C*H*$_2$-(*Ar*)C-CH-CH-C-C(CH$_3$)$_3$), 1.75 (2H, sept, 2x(*Ar*)C-CH-CH-C-C*H*(CH$_3$)$_2$, $^3J_{H,H}$ = 6.9 Hz), 1.48 (6H, s, 2xC*H*$_3$-(*Ar*)C-CH-CH-C), 1.35 (9H, s, S-CH$_2$-(*Ar*)C-CH-CH-C-C(C*H*$_3$)$_3$), 1.30 (9H, s, S-CH$_2$-(*Ar*)C-CH-CH-C-C(C*H*$_3$)$_3$), 0.80 (6H, d, (*Ar*)C-CH-CH-C-CH(C*H*$_3$)$_2$, $^3J_{H,H}$ = 6.9 Hz), 0.76 (6H, d, (*Ar*)C-CH-CH-C-CH(C*H*$_3$)$_2$, $^3J_{H,H}$ = 6.8 Hz).

^{13}C-NMR (DMSO-*d$_6$*) δ$_C$, ppm: 155.9 (1C, N-CH-N-C-C-NH$_2$), 152.5 (1C, N-CH-N-C-NH$_2$), 150.5, 150.4 (3C, 2xS-CH$_2$-(*Ar*)C-CH-CH-C-C(CH$_3$)$_3$), 149.2 (1C, (*Tr*)N-N=N-CH$_2$-N-C-N), 142.8 (1C, (*Tr*)N-N=N-C-CH), 140.6 (1C, N-CH-N-C-C-NH$_2$), 138.6 (1C, S-(*Ar*)C-C-CH-CH-CH-CH), 137.7 (1C, S-(*Ar*)C-C-CH-CH-CH-CH), 136.9, 136.8 (2C, 2xS-CH$_2$-(*Ar*)C-CH-CH-C-C(CH$_3$)$_3$), 133.8 (1C, S-(*Ar*)C-CH-CH-CH-CH), 129.9 (1C, S-(*Ar*)C-CH-CH-CH-CH), 129.6, 128.9 (4C, 4xS-CH$_2$-(*Ar*)C-CH-CH-C-C(CH$_3$)$_3$), 129.1 (1C, S-(*Ar*)C-CH-CH-CH-CH), 128.5 (1C, S-(*Ar*)C-CH-CH-CH-CH), 125.2, 125.1 (4C, 4xS-CH$_2$-(*Ar*)C-CH-CH-C-C(CH$_3$)$_3$), 124.1 (1C, (*Tr*)N-N=N-C-CH), 118.6 (1C, N-CH-N-C-C-NH$_2$), 105.3 (2C, 2xCH$_3$-(*Ar*)C-CH-CH-C), 101.0 (2C, 2xCH$_3$-(*Ar*)C-CH-CH-C), 83.7 (2C, 2xCH$_3$-(*Ar*)C-CH-CH-C), 82.7 (2C, 2xCH$_3$-(*Ar*)C-CH-CH-C), 82.4 (2C, 2xCH$_3$-(*Ar*)C-CH-CH-C), 82.2 (2C, 2xCH$_3$-(*Ar*)C-CH-CH-C), 51.9 (1C, S-(*Ar*)-C-C-CH$_2$-(*Tr*)N-N=N), 38.5 (1C, S-CH$_2$-(*Ar*)C-CH-CH-C-C(CH$_3$)$_3$), 37.9 (2C, S-CH$_2$-(*Ar*)C-CH-CH-C-C(CH$_3$)$_3$, (*Tr*)N-N=N-C-CH$_2$), 34.43, 34.37 (2C, 2xS-CH$_2$-(*Ar*)C-CH-CH-C-C(CH$_3$)$_3$), 31.21, 31.15 (6C, 2xS-CH$_2$-(*Ar*)C-CH-CH-C-C(CH$_3$)$_3$), 30.0 (2C, 2x(*Ar*)CH-CH-C-CH(CH$_3$)$_2$), 22.6 (2C, (*Ar*)CH-CH-C-CH(CH$_3$)$_2$), 22.0 (2C, (*Ar*)CH-CH-C-CH(CH$_3$)$_2$), 17.3 (2C, 2xCH$_3$-(*Ar*)C-CH-CH).

R*$_f$* (CH$_2$Cl$_2$/CH$_3$OH 10:1) = 0.135.

ESI-MS(+): *m/z* found 1167.3052 [M-Cl]$^+$, 584.1557 [M-Cl+H]$^{2+}$, calcd. for $C_{57}H_{71}N_8Ru_2S_3^+$ 1167.3045 and for $C_{57}H_{72}N_8Ru_2S_3^{2+}$ 584.1559.

Elemental analysis (%): calcd. for $C_{57}H_{71}N_8Ru_2S_3Cl\cdot 0.2CH_2Cl_2\cdot CH_3OH$ C 55.88, H 6.08, N 8.96; found C 55.91, H 6.08, N 8.92.

Synthesis of [(*η*^{6}-*p*-MeC$_6$H$_4$Pr^i)$_2$Ru$_2$(*μ*$_2$-SCH$_2$C$_6$H$_4$-*p*-Bu^t)$_2$(*μ*$_2$-SC$_6$H$_4$-*p*-R)]Cl (R = 1-((1*H*-1,2,3-triazol-4-yl)methyl)pyrimidine-2,4(1*H*,3*H*)-dione) (23)

To a solution of **10** (0.281 g, 0.268 mmol, 1 equiv.) and **15** (0.048 g, 0.322 mmol, 1.2 equiv.) in dry DMF (10 mL) were added successively $CuSO_4\cdot 5H_2O$ (0.067 g, 0.268 mmol, 1 equiv.) and sodium ascorbate (0.106 g, 0.536 mmol, 2 equiv.). The reaction mixture was stirred at 60°C under inert atmosphere (N_2) for further 24 h and the reaction evolution was verified by TLC. The reaction mixture was diluted with EtOAc (100 mL) and washed with H_2O (2×100 mL); the unified aqueous phases were further washed with EtOAc (100 mL). The combined organic phases were washed with brine (100 mL), dried over anhydrous Na_2SO_4, filtered and concentrated to dryness under reduced pressure. Purification by column chromatography (CH_2Cl_2/CH_3OH 9.5:0.5 (v/v)) afforded **23** as an orange solid (0.054 g, 0.046 mmol, yield 17%).

^{1}H-NMR (CDCl$_3$) δ$_H$, ppm: 9.40 (1H, s, (*Tr*)N-N=N-C-C*H*), 8.97 (1H, s, CH$_2$-N-(C=O)-N*H*-(C=O), 8.04 (1H, d, CH$_2$-N-CH-CH-(C=O), $^3J_{H,H}$ = 8.0 Hz), 8.01 (2H, d, 2xS-(*Ar*)C-CH-C*H*-C-(*Tr*)N, $^3J_{H,H}$ = 8.6 Hz), 7.93 (2H, d, 2xS-(*Ar*)C-C*H*-CH-C-(*Tr*)N, $^3J_{H,H}$ = 8.5 Hz), 7.38-7.50 (8H, m, 4xS-CH$_2$-(*Ar*)C-C*H*-CH-C-C(CH$_3$)$_3$, 4xS-CH$_2$-(*Ar*)C-CH-C*H*-C-C(CH$_3$)$_3$, $^3J_{H,H}$ = 8.5 Hz), 5.74 (1H, dd, CH$_2$-N-CH-C*H*-(C=O), $^3J_{H,H}$ = 7.8 Hz, $^4J_{H,H}$ = 1.6 Hz), 5.25 (2H, s br, (*Tr*)N-N=N-C-C*H*$_2$-N-(C=O)-NH), 5.10 (2H, d, 2xCH$_3$-(*Ar*)C-CH-C*H*-C, $^3J_{H,H}$ = 5.7 Hz), 5.01 (2H, d, 2xCH$_3$-(*Ar*)C-CH-C*H*-C, $^3J_{H,H}$ = 5.8 Hz), 4.89 (2H, d, 2xCH$_3$-(*Ar*)C-C*H*-CH-C, $^3J_{H,H}$ = 5.7 Hz), 4.67 (2H, d, 2xCH$_3$-(*Ar*)C-C*H*-CH-C, $^3J_{H,H}$ = 5.8 Hz), 3.61 (2H, s, S-C*H*$_2$-(*Ar*)C-CH-CH-C-C(CH$_3$)$_3$), 3.40 (2H, s, S-C*H*$_2$-(*Ar*)C-CH-CH-C-

C(CH$_3$)$_3$), 1.98 (2H, sept, 2x(*Ar*)C-CH-CH-C-C*H*(CH$_3$)$_2$, $^3J_{H,H}$ = 6.8 Hz), 1.73 (6H, s, 2xC*H*$_3$-(*Ar*)C-CH-CH-C), 1.36 (9H, s, S-CH$_2$-(*Ar*)C-CH-CH-C-C(C*H*$_3$)$_3$), 1.32 (9H, s, S-CH$_2$-(*Ar*)C-CH-CH-C-C(C*H*$_3$)$_3$), 0.96 (6H, d, (*Ar*)C-CH-CH-C-CH(C*H*$_3$)$_2$, $^3J_{H,H}$ = 6.8 Hz), 0.91 (6H, d, (*Ar*)C-CH-CH-C-CH(C*H*$_3$)$_2$, $^3J_{H,H}$ = 6.9 Hz).

^{13}C-NMR (CDCl$_3$) δ_C, ppm: 163.8 (1C, NH-(*C*=O)-CH-CH), 152.1, 152.0 (2C, 2xS-CH$_2$-(*Ar*)C-CH-CH-*C*-C(CH$_3$)$_3$), 151.1 (1C, NH-(*C*=O)-N-CH$_2$), 145.5 (1C, NH-(C=O)-CH-*C*H), 143.3 (1C, (*Tr*)N-N=N-*C*-CH), 138.5 (1C, S-(*Ar*)C-CH-CH-*C*-(*Tr*)N), 137.1 (1C, S-(*Ar*)*C*-CH-CH-C-(*Tr*)N), 136.6, 136.4 (2C, 2xS-CH$_2$-(*Ar*)*C*-CH-CH-C-C(CH$_3$)$_3$), 134.1 (2C, 2xS-(*Ar*)C-*C*H-CH-C-(*Tr*)N), 129.3, 129.1 (4C, 4xS-CH$_2$-(*Ar*)C-*C*H-CH-C-C(CH$_3$)$_3$), 125.8, 125.6 (4C, 4xS-CH$_2$-(*Ar*)C-CH-*C*H-C-C(CH$_3$)$_3$), 123.7 (1C, (*Tr*)N-N=N-C-*C*H), 120.7 (2C, 2xS-(*Ar*)C-CH-*C*H-C-(*Tr*)N), 107.7 (2C, 2xCH$_3$-(*Ar*)C-CH-CH-*C*), 102.7 (1C, NH-(C=O)-*C*H-CH), 100.4 (2C, 2xCH$_3$-(*Ar*)*C*-CH-CH-C), 84.11 (2C, 2xCH$_3$-(*Ar*)C-CH-*C*H-C), 84.07 (2C, 2xCH$_3$-(*Ar*)C-*C*H-CH-C), 83.6 (2C, 2xCH$_3$-(*Ar*)C-CH-*C*H-C), 82.5 (2C, 2xCH$_3$-(*Ar*)C-*C*H-CH-C), 42.6 (1C, S-(*Ar*)C-CH-CH-C-(*Tr*)N-N=N-C-*C*H$_2$-N-(C=O)), 40.0 (1C, S-*C*H$_2$-(*Ar*)C-CH-CH-C-C(CH$_3$)$_3$), 39.6 (1C, S-*C*H$_2$-(*Ar*)C-CH-CH-C-C(CH$_3$)$_3$), 34.91, 34.87 (2C, 2xS-CH$_2$-(*Ar*)C-CH-CH-C-*C*(CH$_3$)$_3$), 31.52, 31.50 (6C, 2xS-CH$_2$-(*Ar*)C-CH-CH-C-C(*C*H$_3$)$_3$), 31.1 (2C, 2x(*Ar*)CH-CH-C-*C*H(CH$_3$)$_2$), 23.1 (2C, (*Ar*)CH-CH-C-CH(*C*H$_3$)$_2$), 22.9 (2C, (*Ar*)CH-CH-C-CH(*C*H$_3$)$_2$), 18.3 (2C, 2x*C*H$_3$-(*Ar*)C-CH-CH).

R*$_f$* (CH$_2$Cl$_2$/CH$_3$OH 10:1) = 0.177.

ESI-MS(+): *m/z* found 1130.2594 [M-Cl]$^+$, calcd. for $C_{55}H_{68}N_5O_2Ru_2S_3^+$ 1130.2617.

Elemental analysis (%): calcd. for $C_{55}H_{68}ClN_5O_2Ru_2S_3\cdot 0.1CH_2Cl_2\cdot 1.7CH_3OH$ C 55.56, H 6.16, N 5.70; found C 55.56, H 6.16, N 5.25.

Synthesis of [(η^6-*p*-MeC$_6$H$_4$Pr^i)$_2$Ru$_2$(μ_2-SCH$_2$C$_6$H$_4$-*p*-Bu^t)$_2$(μ_2-SC$_6$H$_4$-*p*-R)]Cl (R = 5-methyl-1-((1*H*-1,2,3-triazol-4-yl)methyl)pyrimidine-2,4(1*H*,3*H*)-dione) (24)

To a solution of **10** (0.329 g, 0.315 mmol, 1 equiv.), and **16** (0.062 g, 0.378 mmol, 1.2 equiv.) in dry DMF (10 mL), were added successively $CuSO_4\cdot 5H_2O$ (0.079 g, 0.315 mmol, 1 equiv.) and sodium ascorbate (0.125 g, 0.630 mmol, 2 equiv.). The reaction mixture was stirred at 60°C under inert atmosphere (N_2) for further 24 h and the reaction evolution was verified by TLC. The reaction mixture was solubilised in EtOAc (100 mL) and washed with H_2O (2×100 mL); the unified aqueous phases were further washed with EtOAc (100 mL). The combined organic phases were washed with brine (100 mL), dried over anhydrous Na_2SO_4, filtered and concentrated to dryness under reduced pressure. Purification by column chromatography (CH$_2$Cl$_2$/CH$_3$OH 9.5:0.5 (v/v)) afforded **24** as an orange solid (0.112 g, 0.095 mmol, yield 30%).

^{1}H-NMR (CDCl$_3$) δ_H, ppm: 9.41 (1H, s, (*Tr*)N-N=N-C-C*H*), 8.64 (1H, s, CH$_2$-N-(C=O)-N*H*-(C=O), 8.04 (2H, d, 2xS-(*Ar*)C-CH-C*H*-C-(*Tr*)N, $^3J_{H,H}$ = 8.3 Hz), 7.94 (2H, d, 2xS-(*Ar*)C-C*H*-CH-C-(*Tr*)N, $^3J_{H,H}$ = 8.2 Hz), 7.81 (1H, s, CH$_2$-N-*C*H-C-CH$_3$), 7.37-7.50 (8H, m, 4xS-CH$_2$-(*Ar*)C-C*H*-CH-C-C(CH$_3$)$_3$, 4xS-CH$_2$-(*Ar*)C-CH-C*H*-C-C(CH$_3$)$_3$, $^3J_{H,H}$ = 8.6 Hz), 5.19 (2H, s br, (*Tr*)N-N=N-C-C*H*$_2$-N-(C=O)-NH), 5.10 (2H, d, 2xCH$_3$-(*Ar*)C-CH-C*H*-C, $^3J_{H,H}$ = 5.7 Hz), 5.01 (2H, d, 2xCH$_3$-(*Ar*)C-CH-C*H*-C, $^3J_{H,H}$ = 5.8 Hz), 4.89 (2H, d, 2xCH$_3$-(*Ar*)C-C*H*-CH-C, $^3J_{H,H}$ = 5.6 Hz), 4.67 (2H, d, 2xCH$_3$-(*Ar*)C-C*H*-CH-C, $^3J_{H,H}$ = 5.8 Hz), 3.61 (2H, s, S-C*H*$_2$-(*Ar*)C-CH-CH-C-C(CH$_3$)$_3$), 3.40 (2H, s, S-C*H*$_2$-(*Ar*)C-CH-CH-C-C(CH$_3$)$_3$), 1.99 (2H, sept, 2x(*Ar*)C-CH-CH-C-C*H*(CH$_3$)$_2$, $^3J_{H,H}$ = 6.9 Hz), 1.95 (3H, s, CH$_2$-N-CH-C-C*H*$_3$), 1.73 (6H, s, 2xC*H*$_3$-(*Ar*)C-CH-CH-C), 1.36 (9H, s, S-CH$_2$-(*Ar*)C-CH-CH-C-C(C*H*$_3$)$_3$), 1.32 (9H, s, S-CH$_2$-(*Ar*)C-CH-CH-C-C(C*H*$_3$)$_3$), 0.96 (6H, d, (*Ar*)C-CH-CH-C-CH(C*H*$_3$)$_2$, $^3J_{H,H}$ = 6.8 Hz), 0.92 (6H, d, (*Ar*)C-CH-CH-C-CH(C*H*$_3$)$_2$, $^3J_{H,H}$ = 6.9 Hz).

^{13}C-NMR (CDCl$_3$) δ_C, ppm: 164.4 (1C, (*C*=O)-NH-(C=O)-N-CH$_2$), 152.04, 151.96 (2C, 2xS-CH$_2$-(*Ar*)C-CH-CH-*C*-C(CH$_3$)$_3$), 151.2 (1C, (C=O)-NH-(*C*=O)-N-CH$_2$), 143.4 (1C, (*Tr*)N-N=N-*C*-CH),

141.1 (1C, (C=O)-N-$\underline{C}$H-C-CH$_3$), 138.5 (1C, S-(*Ar*)C-CH-CH-$\underline{C}$-(*Tr*)N), 137.0 (1C, S-(*Ar*)$\underline{C}$-CH-CH-C-(*Tr*)N), 136.6, 136.5 (2C, 2xS-CH$_2$-(*Ar*)$\underline{C}$-CH-CH-C-C(CH$_3$)$_3$), 134.1 (2C, 2xS-(*Ar*)C-$\underline{C}$H-CH-C-(*Tr*)N), 129.3, 129.1 (4C, 4xS-CH$_2$-(*Ar*)C-$\underline{C}$H-CH-C-C(CH$_3$)$_3$), 125.7, 125.6 (4C, 4xS-CH$_2$-(*Ar*)C-CH-$\underline{C}$H-C-C(CH$_3$)$_3$), 123.5 (1C, (*Tr*)N-N=N-C-$\underline{C}$H), 120.7 (2C, 2xS-(*Ar*)C-CH-$\underline{C}$H-C-(*Tr*)N), 111.3 (1C, (C=O)-N-CH-$\underline{C}$-CH$_3$), 107.7 (2C, 2xCH$_3$-(*Ar*)C-CH-CH-$\underline{C}$), 100.4 (2C, 2xCH$_3$-(*Ar*)$\underline{C}$-CH-CH-C), 84.10 (2C, 2xCH$_3$-(*Ar*)C-CH-$\underline{C}$H-C), 84.08 (2C, 2xCH$_3$-(*Ar*)C-$\underline{C}$H-CH-C), 83.6 (2C, 2xCH$_3$-(*Ar*)C-CH-$\underline{C}$H-C), 82.5 (2C, 2xCH$_3$-(*Ar*)C-$\underline{C}$H-CH-C), 42.4 (1C, S-(*Ar*)C-CH-CH-C-(*Tr*)N-N=N-C-$\underline{C}$H$_2$-N-(C=O)), 40.0 (1C, S-$\underline{C}$H$_2$-(*Ar*)C-CH-CH-C-C(CH$_3$)$_3$), 39.6 (1C, S-$\underline{C}$H$_2$-(*Ar*)C-CH-CH-C-C(CH$_3$)$_3$), 34.90, 34.86 (2C, 2xS-CH$_2$-(*Ar*)C-CH-CH-C-$\underline{C}$(CH$_3$)$_3$), 31.51, 31.49 (6C, 2xS-CH$_2$-(*Ar*)C-CH-CH-C-C($\underline{C}$H$_3$)$_3$), 31.1 (2C, 2x(*Ar*)CH-CH-C-$\underline{C}$H(CH$_3$)$_2$), 23.1 (2C, (*Ar*)CH-CH-C-CH($\underline{C}$H$_3$)$_2$), 22.9 (2C, (*Ar*)CH-CH-C-CH($\underline{C}$H$_3$)$_2$), 18.3 (2C, 2x$\underline{C}$H$_3$-(*Ar*)C-CH-CH), 12.4 (1C, CH$_2$-N-CH-C-$\underline{C}$H$_3$).
R*f* (CH$_2$Cl$_2$/CH$_3$OH 10:1) = 0.198.
ESI-MS(+): *m/z* found 1144.2745 [M-Cl]$^+$, calcd. for C$_{56}$H$_{70}$N$_5$O$_2$Ru$_2$S$_3^+$ 1144.2773.
Elemental analysis (%): calcd. for C$_{56}$H$_{70}$ClN$_5$O$_2$Ru$_2$S$_3$·0.8CH$_3$OH C 56.63, H 6.13, N 5.81; found C 56.63, H 6.37, N 5.18.

Synthesis of [(η^6-*p*-MeC$_6$H$_4$Pr^i)$_2$Ru$_2$(μ_2-SCH$_2$C$_6$H$_4$-*p*-Bu^t)$_2$(μ_2-SC$_6$H$_4$-*p*-R)]Cl (R = 4-amino-1-((1*H*-1,2,3-triazol-4-yl)methyl)pyrimidin-2(1*H*)-one) (25)

To a solution of **10** (0.287 g, 0.275 mmol, 1 equiv.) and **17** (0.062 g, 0.416 mmol, 1.5 equiv.) in dry DMF (10 mL), were added successively CuSO$_4$·5H$_2$O (0.069 g, 0.275 mmol, 1 equiv.) and sodium ascorbate (0.109 g, 0.550 mmol, 2 equiv.). The reaction mixture was stirred at 60°C under inert atmosphere (N$_2$) for 24 h, the reaction evolution being verified by TLC. The reaction mixture was diluted with EtOAc (100 mL) and washed with H$_2$O (2×100 mL); the unified aqueous phases were further washed with EtOAc (100 mL). The combined organic phases were washed with brine (100 mL), dried over anhydrous Na$_2$SO$_4$, filtered and concentrated to dryness under reduced pressure. Purification by column chromatography (CH$_2$Cl$_2$/CH$_3$OH 9:1 (v/v)) afforded **25** as an orange solid (0.075 g, 0.064 mmol, yield 23%).
^{1}H-NMR (CDCl$_3$) δ_H, ppm: 8.52 (1H, s, (*Tr*)N-N=N-C-C$\underline{H}$), 7.94 (2H, d, 2xS-(*Ar*)C-CH-C$\underline{H}$-C-(*Tr*)N, $^3J_{H,H}$ = 8.7 Hz), 7.83 (2H, d, 2xS-(*Ar*)C-C$\underline{H}$-CH-C-(*Tr*)N, $^3J_{H,H}$ = 8.7 Hz), 7.54 (1H, d, CH$_2$-N-$\underline{C}$H-CH-C-NH$_2$, $^3J_{H,H}$ = 7.4 Hz), 7.38-7.51 (8H, m, 4xS-CH$_2$-(*Ar*)C-C$\underline{H}$-CH-C-C(CH$_3$)$_3$, 4xS-CH$_2$-(*Ar*)C-CH-C$\underline{H}$-C-C(CH$_3$)$_3$, $^3J_{H,H}$ = 8.6 Hz), 6.50 (1H, d, CH$_2$-N-CH-C$\underline{H}$-C-NH$_2$, $^3J_{H,H}$ = 7.3 Hz), 5.13 (2H, d, 2xCH$_3$-(*Ar*)C-CH-C$\underline{H}$-C, $^3J_{H,H}$ = 5.2 Hz), 5.12 (2H, s, (*Tr*)N-N=N-C-C$\underline{H_2}$-N-(C=O)-N), 5.03 (2H, d, 2xCH$_3$-(*Ar*)C-CH-C$\underline{H}$-C, $^3J_{H,H}$ = 5.9 Hz), 4.90 (2H, d, 2xCH$_3$-(*Ar*)C-C$\underline{H}$-CH-C, $^3J_{H,H}$ = 5.8 Hz), 4.67 (2H, d, 2xCH$_3$-(*Ar*)C-C$\underline{H}$-CH-C, $^3J_{H,H}$ = 5.8 Hz), 3.62 (2H, s, S-C$\underline{H_2}$-(*Ar*)C-CH-CH-C-C(CH$_3$)$_3$), 3.44 (2H, s, S-C$\underline{H_2}$-(*Ar*)C-CH-CH-C-C(CH$_3$)$_3$), 1.98 (2H, sept, 2x(*Ar*)C-CH-CH-C-C$\underline{H}$(CH$_3$)$_2$, $^3J_{H,H}$ = 6.8 Hz), 1.74 (6H, s, 2xC$\underline{H_3}$-(*Ar*)C-CH-CH-C), 1.37 (9H, s, S-CH$_2$-(*Ar*)C-CH-CH-C-C($\underline{CH_3)_3}$), 1.33 (9H, s, S-CH$_2$-(*Ar*)C-CH-CH-C-C($\underline{CH_3)_3}$), 0.96 (6H, d, (*Ar*)C-CH-CH-C-CH(C$\underline{H_3}$)$_2$, $^3J_{H,H}$ = 6.8 Hz), 0.92 (6H, d, (*Ar*)C-CH-CH-C-CH(C$\underline{H_3}$)$_2$, $^3J_{H,H}$ = 6.9 Hz).
^{13}C-NMR (CDCl$_3$) δ_C, ppm: 165.7 (1C, N-(C=O)-N-$\underline{C}$-NH$_2$), 156.0 (1C, N-($\underline{C}$=O)-N-CH$_2$), 152.04, 151.96 (2C, 2xS-CH$_2$-(*Ar*)C-CH-CH-$\underline{C}$-C(CH$_3$)$_3$), 145.0 (1C, N-$\underline{C}$H-CH-C-NH$_2$), 144.2 (*Tr*)N-N=N-$\underline{C}$-CH), 138.9 (1C, S-(*Ar*)C-CH-CH-$\underline{C}$-(*Tr*)N), 136.9 (1C, S-(*Ar*)$\underline{C}$-CH-CH-C-(*Tr*)N), 136.6 (2C, 2xS-CH$_2$-(*Ar*)$\underline{C}$-CH-CH-C-C(CH$_3$)$_3$), 134.2 (2C, 2xS-(*Ar*)C-$\underline{C}$H-CH-C-(*Tr*)N), 129.4, 129.2 (4C, 4xS-CH$_2$-(*Ar*)C-$\underline{C}$H-CH-C-C(CH$_3$)$_3$), 125.8, 125.6 (4C, 4xS-CH$_2$-(*Ar*)C-CH-$\underline{C}$H-C-C(CH$_3$)$_3$), 122.5 (1C, (*Tr*)N-N=N-C-$\underline{C}$H), 120.7 (2C, 2xS-(*Ar*)C-CH-$\underline{C}$H-C-(*Tr*)N), 107.7 (2C, 2xCH$_3$-(*Ar*)C-CH-CH-$\underline{C}$), 100.5 (2C, 2xCH$_3$-(*Ar*)$\underline{C}$-CH-CH-C), 96.6 (1C, N-CH-$\underline{C}$H-C-NH$_2$), 84.2 (4C, 2xCH$_3$-(*Ar*)C-$\underline{C}$H-CH-C, 2xCH$_3$-(*Ar*)C-CH-$\underline{C}$H-C), 83.7 (2C, 2xCH$_3$-(*Ar*)C-CH-$\underline{C}$H-C), 82.6 (2C, 2xCH$_3$-(*Ar*)C-$\underline{C}$H-CH-C), 44.9 (1C,

S-(*Ar*)C-CH-CH-C-(*Tr*)N-N=N-C-$\underline{C}H_2$-N-(C=O)), 40.2 (1C, S-$\underline{C}H_2$-(*Ar*)C-CH-CH-C-C$(CH_3)_3$), 39.7 (1C, S-$\underline{C}H_2$-(*Ar*)C-CH-CH-C-C$(CH_3)_3$), 34.9 (2C, 2xS-CH_2-(*Ar*)C-CH-CH-C-$\underline{C}(CH_3)_3$), 31.6 (6C, 2xS-CH_2-(*Ar*)C-CH-CH-C-C$(\underline{C}H_3)_3$), 31.1 (2C, 2x(*Ar*)CH-CH-C-$\underline{C}H(CH_3)_2$), 23.1 (2C, (*Ar*)CH-CH-C-CH$(\underline{C}H_3)_2$), 22.9 (2C, (*Ar*)CH-CH-C-CH$(\underline{C}H_3)_2$), 18.3 (2C, 2x$\underline{C}H_3$-(*Ar*)C-CH-CH).
R*f* (CH_2Cl_2/CH_3OH 9:1) = 0.124.
ESI-MS(+): *m/z* found 1129.2778 $[M-Cl]^+$, calcd. for $C_{55}H_{69}N_6ORu_2S_3^+$ 1129.2776.
Elemental analysis (%): calcd. for $C_{55}H_{69}ClN_6ORu_2S_3\cdot0.2CH_2Cl_2\cdot1.25CH_3OH$ C 55.53, H 6.14, N 6.88; found C 55.57, H 6.14, N 6.71.

Synthesis of $[(\eta^6\text{-}p\text{-}MeC_6H_4Pr^i)_2Ru_2(\mu_2\text{-}SCH_2C_6H_4\text{-}p\text{-}Bu^t)_2(\mu_2\text{-}SC_6H_4\text{-}p\text{-}R)]Cl$ (R = 9-((1*H*-1,2,3-triazol-4-yl)methyl)-9*H*-purin-6-amine) (26)

To a solution of **10** (0.298 g, 0.285 mmol, 1 equiv.) and **18** (0.059 g, 0.342 mmol, 1.2 equiv.) in dry DMF (10 mL), were added successively $CuSO_4\cdot5H_2O$ (0.071 g, 0.285 mmol, 1 equiv.) and sodium ascorbate (0.113 g, 0.570 mmol, 2 equiv.). The reaction mixture was stirred at 60°C under inert atmosphere (N_2) for further 24 h and the reaction evolution was verified by TLC. The reaction mixture was diluted with EtOAc (100 mL) and washed with H_2O (2×100 mL); the unified aqueous phases were further washed with EtOAc (100 mL). The combined organic phases were washed with brine (100 mL), dried over anhydrous Na_2SO_4, filtered and concentrated to dryness under reduced pressure. Purification by column chromatography (CH_2Cl_2/CH_3OH 10:1 (v/v)) afforded **26** as an orange solid (0.091 g, 0.077 mmol, yield 27%).
^{1}H-NMR ($CDCl_3$) δ_H, ppm: 9.16 (1H, s br, (*Tr*)N-N=N-C-C$\underline{H}$), 8.36 (1H, s br, N-C$\underline{H}$-N-C-NH_2), 8.29 (1H, s br, CH_2-N-C$\underline{H}$-NH-C-C-NH_2), 7.95 (2H, d, 2xS-(*Ar*)C-CH-C$\underline{H}$-C-(*Tr*)N, $^3J_{H,H}$ = 8.9 Hz), 7.91 (2H, d, 2xS-(*Ar*)C-C$\underline{H}$-CH-C-(*Tr*)N, $^3J_{H,H}$ = 8.9 Hz), 7.36-7.48 (8H, m, 4xS-CH_2-(*Ar*)C-C$\underline{H}$-CH-C-C$(CH_3)_3$, 4xS-CH_2-(*Ar*)C-CH-C$\underline{H}$-C-C$(CH_3)_3$, $^3J_{H,H}$ = 8.5 Hz), 6.20 (2H, s br, N-CH-N-C-C-N$\underline{H_2}$), 5.66 (2H, s, (*Tr*)N-N=N-C-C$\underline{H_2}$-N-CH-N), 5.07 (2H, d, 2xCH_3-(*Ar*)C-CH-C$\underline{H}$-C, $^3J_{H,H}$ = 5.8 Hz), 4.98 (2H, d, 2xCH_3-(*Ar*)C-CH-C$\underline{H}$-C, $^3J_{H,H}$ = 5.8 Hz), 4.89 (2H, d, 2xCH_3-(*Ar*)C-C$\underline{H}$-CH-C, $^3J_{H,H}$ = 5.8 Hz), 4.63 (2H, d, 2xCH_3-(*Ar*)C-C$\underline{H}$-CH-C, $^3J_{H,H}$ = 5.8 Hz), 3.58 (2H, s, S-C$\underline{H_2}$-(*Ar*)C-CH-CH-C-C$(CH_3)_3$), 3.38 (2H, s, S-C$\underline{H_2}$-(*Ar*)C-CH-CH-C-C$(CH_3)_3$), 1.95 (2H, sept, 2x(*Ar*)C-CH-CH-C-C$\underline{H}(CH_3)_2$, $^3J_{H,H}$ = 6.8 Hz), 1.70 (6H, s, 2xC$\underline{H_3}$-(*Ar*)C-CH-CH-C), 1.33 (9H, s, S-CH_2-(*Ar*)C-CH-CH-C-C(C$\underline{H_3})_3$), 1.29 (9H, s, S-CH_2-(*Ar*)C-CH-CH-C-C(C$\underline{H_3})_3$), 0.92 (6H, d, (*Ar*)C-CH-CH-C-CH(C$\underline{H_3}$)$_2$, $^3J_{H,H}$ = 6.8 Hz), 0.87 (6H, d, (*Ar*)C-CH-CH-C-CH(C$\underline{H_3}$)$_2$, $^3J_{H,H}$ = 6.9 Hz).
^{13}C-NMR ($CDCl_3$) δ_C, ppm: 155.5 (1C, N-CH-N-C-$\underline{C}$-NH_2), 152.9 (1C, N-$\underline{C}$H-N-C-NH_2), 152.0, 151.9 (2C, 2xS-CH_2-(*Ar*)C-CH-CH-$\underline{C}$-C$(CH_3)_3$), 150.0 (1C, N-$\underline{C}$-N-CH_2-(*Tr*)C), 143.6 (*Tr*)N-N=N-$\underline{C}$-CH), 141.2 (1C, N-$\underline{C}$H-N-C-C-NH_2), 138.6 (1C, S-(*Ar*)C-CH-CH-$\underline{C}$-(*Tr*)N), 136.9 (1C, S-(*Ar*)$\underline{C}$-CH-CH-C-(*Tr*)N), 136.6, 136.5 (2C, 2xS-CH_2-(*Ar*)$\underline{C}$-CH-CH-C-C$(CH_3)_3$), 134.1 (2C, 2xS-(*Ar*)C-$\underline{C}$H-CH-C-(*Tr*)N), 129.3, 129.1 (4C, 4xS-CH_2-(*Ar*)C-$\underline{C}$H-CH-C-C$(CH_3)_3$), 125.7, 125.6 (4C, 4xS-CH_2-(*Ar*)C-CH-$\underline{C}$H-C-C$(CH_3)_3$), 122.8 (1C, (*Tr*)N-N=N-C-$\underline{C}$H), 120.7 (2C, 2xS-(*Ar*)C-CH-$\underline{C}$H-C-(*Tr*)N), 119.4 (1C, N-CH-N-$\underline{C}$-C-NH_2), 107.5 (2C, 2xCH_3-(*Ar*)C-CH-CH-$\underline{C}$), 100.4 (2C, 2xCH_3-(*Ar*)$\underline{C}$-CH-CH-C), 84.0 (4C, 2xCH_3-(*Ar*)C-$\underline{C}$H-CH-C, 2xCH_3-(*Ar*)C-CH-$\underline{C}$H-C), 83.7 (2C, 2xCH_3-(*Ar*)C-CH-$\underline{C}$H-C), 82.4 (2C, 2xCH_3-(*Ar*)C-$\underline{C}$H-CH-C), 40.0 (1C, S-$\underline{C}H_2$-(*Ar*)C-CH-CH-C-C$(CH_3)_3$), 39.6 (1C, S-$\underline{C}H_2$-(*Ar*)C-CH-CH-C-C$(CH_3)_3$), 38.5 (1C, S-(*Ar*)C-CH-CH-C-(*Tr*)N-N=N-C-$\underline{C}H_2$-N-C-N), 34.85, 34.81 (2C, 2xS-CH_2-(*Ar*)C-CH-CH-C-$\underline{C}(CH_3)_3$), 31.48, 31.45 (6C, 2xS-CH_2-(*Ar*)C-CH-CH-C-C$(\underline{C}H_3)_3$), 31.1 (2C, 2x(*Ar*)CH-CH-C-$\underline{C}H(CH_3)_2$), 23.0 (2C, (*Ar*)CH-CH-C-CH$(\underline{C}H_3)_2$), 22.8 (2C, (*Ar*)CH-CH-C-CH$(\underline{C}H_3)_2$), 18.2 (2C, 2x$\underline{C}H_3$-(*Ar*)C-CH-CH).
R*f* (CH_2Cl_2/CH_3OH 10:1) = 0.156.
ESI-MS(+): *m/z* found 1153.2896 $[M-Cl]^+$, calcd. for $C_{56}H_{69}N_8Ru_2S_3^+$ 1053.2889.

Elemental analysis (%): calcd. for $C_{56}H_{69}ClN_8Ru_2S_3\cdot0.7CH_2Cl_2$ C 54.59, H 5.69, N 8.98; found C 54.65, H 5.68, N 8.51.

6. Synthesis of compounds **27-33** (family 4)

Synthesis of 6-(azidomethyl)uracil (6-(azidomethyl)pyrimidine-2,4(1*H*,3*H*)-dione) (27)

Compound **27** was prepared by adapting a previously reported procedure [13]. To a solution 6-(chloromethyl)uracil (6-(chloromethyl)pyrimidine-2,4(1*H*,3*H*)-dione) (0.600 g, 3.662 mmol, 1 equiv.) in dry DMF (10 mL) at 50°C under inert atmosphere (N_2) was added NaN_3 (0.476 g, 7.324 mmol, 2 equiv.) and the mixture was stirred overnight. The reaction mixture was allowed to cool to r.t., filtered and washed with CH_2Cl_2. The filtrate was concentrated under reduced pressure affording **27** as white solid (0.133 g, 0.796 mmol, yield 22%).

^{1}H-NMR (DMSO-*d₆*) δ_H, ppm: 10.86 (2H, m br, 2xNH), 5.53 (1H, s, NH-(C=O)-C$\underline{\mathit{H}}$-C-CH_2-N_3), 4.23 (2H, s, NH-(C=O)-CH-C-C$\underline{\mathit{H_2}}$-N_3).

^{13}C-NMR (DMSO-*d₆*) δ_C, ppm: 163.9 (1C, NH-($\underline{\mathit{C}}$=O)-CH-C-CH_2-N_3), 151.4 (1C, NH-(C=O)-CH-$\underline{\mathit{C}}$-CH_2-N_3), 150.5 (1C, NH-($\underline{\mathit{C}}$=O)-NH), 98.7 (1C, NH-(C=O)-$\underline{\mathit{C}}$H-C-CH_2-N_3), 49.4 (1C, NH-(C=O)-CH-C-$\underline{\mathit{C}}H_2$-$N_3$).

R_f (CH_2Cl_2/CH_3OH 10:1) = 0.244.

ESI-MS(+): *m/z* found 199.0705 [M+CH_3OH]$^+$, calcd. for $C_6H_9N_5O_3$ 199.0705.

Elemental analysis (%): calcd. for $C_5H_5N_5O_2\cdot0.3CH_2Cl_2\cdot0.2CH_3OH$ C 33.19, H 3.24, N 35.19; found C 33.22, H 2.74, N 35.18.

Synthesis of [(η^6-*p*-$MeC_6H_4Pr^i$)$_2$$Ru_2$($\mu_2$-$SCH_2C_6H_4$-*p*-$Bu^t$)$_2$($\mu_2$-$SC_6H_4$-*p*-NH-R]Cl (R = 4-(1-((2,6-dioxo-1,2,3,6-tetrahydropyrimidin-4-yl)methyl)-1*H*-1,2,3-triazol-4-yl)-butanamide) (28)

To a solution of **5** (0.221 g, 0.204 mmol, 1 equiv.) in mixture CH_3CN/H_2O (20 mL, 1:1 (v/v)) under inert atmosphere (N_2) at r.t were added successively **27** (0.040 g, 0.239 mmol, 1.2 equiv.), $Cu_2SO_4\cdot5H_2O$ (0.050 g, 0.200 mmol, 1 equiv.) and sodium ascorbate (0.080 g, 0.403 mmol, 2 equiv.). The reaction mixture was stirred at 50°C overnight and the reaction evolution was verified by TLC. The mixture was filtered and the filtrate was diluted with EtOAc (100 mL). The organic phase was washed with H_2O (100 mL), saturated aq. soln. NH_4Cl (100 mL), dried over anhydrous Na_2SO_4, filtered and concentrated under reduced pressure. Purification by column chromatography (CH_2Cl_2/CH_3OH 9:1 (v/v)) afforded **28** as an orange solid (0.080 g, 0.064 mmol, yield 31%).

^{1}H-NMR (DMSO-*d₆*) δ_H, ppm: 11.26 (1H, s br, N$\underline{\mathit{H}}$-(C=O)-NH), 11.12 (1H, s, NH-(C=O)-N$\underline{\mathit{H}}$), 10.14 (1H, s, N$\underline{\mathit{H}}$-(C=O)-$(CH_2)_3$), 8.04 (1H, s, (*Tr*)N-N=N-C-C$\underline{\mathit{H}}$), 7.69 (2H, d, 2xS-(*Ar*)C-CH-C$\underline{\mathit{H}}$-C-NH-(C=O), $^3J_{H,H}$ = 8.6 Hz), 7.61 (2H, d, 2xS-(*Ar*)C-C$\underline{\mathit{H}}$-CH-C-NH-(C=O), $^3J_{H,H}$ = 8.6 Hz), 7.49 (4H, m, 4xS-CH_2-(*Ar*)C-CH-C$\underline{\mathit{H}}$-C-C$(CH_3)_3$, $^3J_{H,H}$ = 8.5 Hz), 7.43 (4H, m, 4xS-CH_2-(*Ar*)C-C$\underline{\mathit{H}}$-CH-C-C$(CH_3)_3$, $^3J_{H,H}$ = 8.3 Hz), 5.34 (2H, d, 2xCH_3-(*Ar*)C-CH-C$\underline{\mathit{H}}$-C, $^3J_{H,H}$ = 5.9 Hz), 5.32 (2H, s br, (*Tr*)N=N-N-C$\underline{\mathit{H_2}}$-C-CH-(C=O)), 5.27 (2H, d, 2xCH_3-(*Ar*)C-CH-C$\underline{\mathit{H}}$-C, $^3J_{H,H}$ = 5.9 Hz), 5.21 (2H, d, 2xCH_3-(*Ar*)C-C$\underline{\mathit{H}}$-CH-C, $^3J_{H,H}$ = 5.7 Hz), 4.94 (1H, s br, (*Tr*)N=N-N-CH_2-C-C$\underline{\mathit{H}}$-(C=O)), 4.73 (2H, d, 2xCH_3-(*Ar*)C-C$\underline{\mathit{H}}$-CH-C, $^3J_{H,H}$ = 5.7 Hz), 3.62 (2H, s, S-C$\underline{\mathit{H_2}}$-(*Ar*)C-CH-CH-C-$(CH_3)_3$), 3.45 (2H, s, S-C$\underline{\mathit{H_2}}$-(*Ar*)C-CH-CH-C-$(CH_3)_3$), 2.71 (2H, t, NH-(C=O)-$(CH_2)_2$-C$\underline{\mathit{H_2}}$-(*Tr*)C, $^3J_{H,H}$ = 7.4 Hz), 2.42 (2H, t, NH-(C=O)-C$\underline{\mathit{H_2}}$-$(CH_2)_2$-(*Tr*)C, $^3J_{H,H}$ = 7.2 Hz), 1.94 (2H, qvint, NH-(C=O)-CH_2-C$\underline{\mathit{H_2}}$-CH_2-(*Tr*)C, $^3J_{H,H}$ = 7.4 Hz), 1.87 (2H, sept, 2x(*Ar*)C-CH-CH-C-C$\underline{\mathit{H}}$$(CH_3)_2$, $^3J_{H,H}$ = 6.8 Hz), 1.72 (6H, s, 2xC$\underline{\mathit{H_3}}$-(*Ar*)C-CH-CH-C), 1.33 (9H, s, S-CH_2-(*Ar*)C-CH-CH-C-C(C$\underline{\mathit{H_3}}$)$_3$), 1.29 (9H, s, S-CH_2-(*Ar*)C-CH-CH-C-C(C$\underline{\mathit{H_3}}$)$_3$), 0.84 (6H, d, (*Ar*)C-CH-CH-C-CH(C$\underline{\mathit{H_3}}$)$_2$, $^3J_{H,H}$ = 6.8 Hz), 0.79 (6H, d, (*Ar*)C-CH-CH-C-CH(C$\underline{\mathit{H_3}}$)$_2$, $^3J_{H,H}$ = 6.8 Hz).

^{13}C-NMR (DMSO-*d₆*) δ_C, ppm: 171.0 (1C, S-(*Ar*)C-CH-CH-C-NH-($\underline{\mathit{C}}$=O)), 163.7 (1C, (*Tr*)C-N=N-N-CH_2-C-CH-($\underline{\mathit{C}}$=O)), 151.2 (1C, (*Tr*)C-N=N-N-CH_2-$\underline{\mathit{C}}$-NH-(C=O)-NH), 150.8 (1C, (*Tr*)C-N=N-N-CH_2-

C-NH-(C=O)-NH), 150.5, 150.3 (2C, 2xS-CH$_2$-(*Ar*)C-CH-CH-C-C(CH$_3$)$_3$), 146.8 (1C, (*Tr*)C-N=N-N-CH$_2$), 139.1 (1C, S-(*Ar*)C-CH-CH-C-NH-(C=O)), 137.0, 136.9 (2C, 2xS-CH$_2$-(*Ar*)C-CH-CH-C-C(CH$_3$)$_3$), 133.0 (2C, 2xS-(*Ar*)C-CH-CH-C-NH-(C=O)), 130.7 (1C, S-(*Ar*)C-CH-CH-C-NH-(C=O)), 129.3, 128.9 (4C, 4xS-CH$_2$-(*Ar*)C-CH-CH-C-C(CH$_3$)$_3$), 125.2, 125.1 (4C, 4xS-CH$_2$-(*Ar*)C-CH-CH-C-C(CH$_3$)$_3$), 123.2 (1C, (*Tr*)C-N=N-N-CH), 118.9 (2C, 2xS-(*Ar*)C-CH-CH-C-NH-C(=O)), 105.6 (2C, 2xCH$_3$-(*Ar*)C-CH-CH-C), 100.9 (2C, 2xCH$_3$-(*Ar*)C-CH-CH-C), 98.3 (1C, (*Tr*)C-N=N-N-CH$_2$-C-CH-(C=O)), 84.4 (2C, 2xCH$_3$-(*Ar*)C-CH-CH-C), 82.9 (4C, 2xCH$_3$-(*Ar*)C-CH-CH-C, 2xCH$_3$-(*Ar*)C-CH-CH-C), 82.1 (2C, 2xCH$_3$-(*Ar*)C-CH-CH-C), 48.7 (1C, (*Tr*)C-N=N-N-CH$_2$-C-CH-(C=O)), 39.5 (1C, S-CH$_2$-(*Ar*)C-CH-CH-C-C(CH$_3$)$_3$), 39.3 (1C, S-CH$_2$-(*Ar*)C-CH-CH-C-C(CH$_3$)$_3$), 35.8 (1C, NH-(C=O)-C*H$_2$*-(CH$_2$)$_2$-(*Tr*)C), 34.40, 34.35 (2C, 2xS-CH$_2$-(*Ar*)C-CH-CH-C-C(CH$_3$)$_3$), 31.2 (6C, 2xS-CH$_2$-(*Ar*)C-CH-CH-C-C(CH$_3$)$_3$), 30.1 (2C, 2x(*Ar*)CH-CH-C-CH(CH$_3$)$_2$), 24.8 (1C, NH-(C=O)-CH$_2$-CH$_2$-CH$_2$-(*Tr*)C), 24.5 (1C, NH-(C=O)-(CH$_2$)$_2$-CH$_2$-(*Tr*)C), 22.9 (2C, (*Ar*)CH-CH-C-CH(CH$_3$)$_2$), 22.0 (2C, (*Ar*)CH-CH-C-CH(CH$_3$)$_2$), 17.5 (2C, 2xCH$_3$-(*Ar*)C-CH-CH).

R*f* (CH$_2$Cl$_2$/CH$_3$OH 10:1) = 0.115.

ESI-MS(+): *m/z* found 1215.3173 [M-Cl]$^+$, calcd. for $C_{59}H_{75}N_6O_3Ru_2S_3^+$ 1215.3139.

Elemental analysis (%): calcd. for $C_{59}H_{75}N_6O_3Ru_2S_3Cl \cdot 3H_2O$ C 54.34, H 6.26, N 6.44; found C 54.30, H 6.61, N 7.30.

Synthesis of [(η^6-*p*-MeC$_6$H$_4$Pr^i)$_2$Ru$_2$(μ_2-SCH$_2$C$_6$H$_4$-*p*-Bu^t)$_2$(μ_2-SC$_6$H$_4$-*p*-NH-R)]Cl (R = 4-(1-((2,6-dioxo-1,2,3,6-tetrahydropyrimidin-4-yl)methyl)-1*H*-1,2,3-triazol-4-yl)-benzamide) (29)

To a solution of **6** (0.147 g, 0.131 mmol, 1 equiv.) in dry DMF (10 mL) under inert atmosphere (N$_2$) at r.t were added successively **27** (0.026 g, 0.155 mmol, 1.2 equiv.), $Cu_2SO_4 \cdot 5H_2O$ (0.032 g, 0.128 mmol, 1 equiv.) and sodium ascorbate (0.052 g, 0.262 mmol, 2 equiv.). The reaction mixture was stirred at 50°C overnight and the reaction evolution was verified by TLC. The mixture was filtered and the filtrate was diluted with EtOAc (100 mL). The organic phase was washed with H$_2$O (2×100 mL), saturated aq. soln. NH$_4$Cl (100 mL), dried over anhydrous Na$_2$SO$_4$, filtered and concentrated under reduced pressure. Purification by column chromatography (CH$_2$Cl$_2$/CH$_3$OH 10:1 (v/v)) afforded **29** as an orange solid (0.122 g, 0.095 mmol, yield 73%).

^{1}H-NMR (CDCl$_3$) δ_H, ppm: 9.82 (1H, s, N*H*-(C=O)-(*Ar*)C-CH-CH-C), 8.37 (1H, s br, (*Tr*)C-N-N=N-C-C*H*), 7.92-8.02 (2H, m br, NH-(C=O)-(*Ar*)C-C*H*-CH-C), 7.82-7.92 (2H, m br, NH-(C=O)-(*Ar*)C-CH-C*H*-C), 7.71 (2H, d, 2xS-(*Ar*)C-CH-C*H*-C-NH-(C=O), $^3J_{H,H}$ = 8.1 Hz), 7.63 (2H, d, 2xS-(*Ar*)C-C*H*-CH-C-NH-(C=O), $^3J_{H,H}$ = 8.2 Hz), 7.28-7.41 (8H, m, 4xS-CH$_2$-(*Ar*)C-C*H*-CH-C-C(CH$_3$)$_3$, 4xS-CH$_2$-(*Ar*)C-CH-C*H*-C-C(CH$_3$)$_3$, $^3J_{H,H}$ = 8.3 Hz), 5.31 (3H, m br, (*Tr*)C-N=N-N-C*H$_2$*-C-NH-(C=O), (*Tr*)C-N=N-N-CH$_2$-C-C*H*-(C=O)), 4.96 (2H, d, 2xCH$_3$-(*Ar*)C-CH-C*H*-C, $^3J_{H,H}$ = 5.6 Hz), 4.84 (2H, d, 2xCH$_3$-(*Ar*)C-CH-C*H*-C, $^3J_{H,H}$ = 5.7 Hz), 4.71 (2H, d, 2xCH$_3$-(*Ar*)C-C*H*-CH-C, $^3J_{H,H}$ = 5.6 Hz), 4.53 (2H, d, 2xCH$_3$-(*Ar*)C-C*H*-CH-C, $^3J_{H,H}$ = 5.7 Hz), 3.49 (2H, s, S-C*H$_2$*-(*Ar*)C-CH-CH-C-(CH$_3$)$_3$), 3.29 (2H, s, S-C*H$_2$*-(*Ar*)C-CH-CH-C-(CH$_3$)$_3$), 1.87 (2H, sept, 2x(*Ar*)C-CH-CH-C-C*H*(CH$_3$)$_2$, $^3J_{H,H}$ = 6.8 Hz), 1.63 (6H s, 2xC*H$_3$*-(*Ar*)C-CH-CH-C), 1.25 (9H, s, S-CH$_2$-(*Ar*)C-CH-CH-C-C(C*H$_3$*)$_3$), 1.23 (9H, s, S-CH$_2$-(*Ar*)C-CH-CH-C-C(C*H$_3$*)$_3$), 0.87 (6H, d, (*Ar*)C-CH-CH-C-CH(C*H$_3$*)$_2$, $^3J_{H,H}$ = 6.8 Hz), 0.83 (6H, d, (*Ar*)C-CH-CH-C-CH(C*H$_3$*)$_2$, $^3J_{H,H}$ = 6.8 Hz).

^{13}C-NMR (CDCl$_3$) δ_C, ppm: 166.5 (1C, S-(*Ar*)C-CH-CH-C-NH-(C=O)), 164.6 (1C, (*Tr*)C-N=N-N-CH$_2$-C-CH-(C=O)), 151.9, 151.8 (2C, 2xS-CH$_2$-(*Ar*)C-CH-CH-C-C(CH$_3$)$_3$), 151.4 (2C, (*Tr*)C-N=N-N-CH$_2$-C-NH-(C=O)-NH, (*Tr*)C-N=N-N-CH$_2$-C-NH-(C=O)-NH), 149.5 (1C, (*Tr*)C-N=N-N-CH$_2$), 139.4 (1C, S-(*Ar*)C-CH-CH-C-NH-(C=O)), 136.44, 136.36 (2C, 2xS-CH$_2$-(*Ar*)C-CH-CH-C-C(CH$_3$)$_3$), 134.2 (1C, NH-(C=O)-(*Ar*)C-CH-CH-C-(*Tr*)C), 133.2 (1C, NH-(C=O)-(*Ar*)C-CH-CH-C-(*Tr*)C), 133.0 (2C, 2xS-(*Ar*)C-CH-CH-C-NH-(C=O)), 132.1 (1C, S-(*Ar*)C-CH-CH-C-NH-(C=O)), 129.1, 128.9 (4C, 4xS-

CH$_2$-(*Ar*)C-CH-CH-C-C(CH$_3$)$_3$), 128.3 (2C, 2xNH-(C=O)-(*Ar*)C-CH-CH-C-(*Tr*)C), 125.8 (2C, 2xNH-(C=O)-(*Ar*)C-CH-CH-C-(*Tr*)C), 125.6, 125.4 (4C, 4xS-CH$_2$-(*Ar*)C-CH-CH-C-C(CH$_3$)$_3$), 120.6 (2C, 2xS-(*Ar*)C-CH-CH-C-NH-C(=O)), 107.3 (2C, 2xCH$_3$-(*Ar*)C-CH-CH-C), 100.3 (3C, 2xCH$_3$-(*Ar*)C-CH-CH-C, (*Tr*)C-N=N-N-CH$_2$-C-CH-(C=O)), 83.8 (2C, 2xCH$_3$-(*Ar*)C-CH-CH-C), 83.55 (2C, 2xCH$_3$-(*Ar*)C-CH-CH-C), 83.52 (2C, 2xCH$_3$-(*Ar*)C-CH-CH-C), 82.2 (2C, 2xCH$_3$-(*Ar*)C-CH-CH-C), 49.4 (1C, (*Tr*)C-N=N-N-CH$_2$-C-CH-(C=O)), 39.8 (1C, S-CH$_2$-(*Ar*)C-CH-CH-C-C(CH$_3$)$_3$), 39.2 (1C, S-CH$_2$-(*Ar*)C-CH-CH-C-C(CH$_3$)$_3$), 34.67, 34.63 (2C, 2xS-CH$_2$-(*Ar*)C-CH-CH-C-C(CH$_3$)$_3$), 30.22, 31.19 (6C, 2xS-CH$_2$-(*Ar*)C-CH-CH-C-C(CH$_3$)$_3$), 30.9 (2C, 2x(*Ar*)CH-CH-C-CH(CH$_3$)$_2$), 22.9 (2C, (*Ar*)CH-CH-C-CH(CH$_3$)$_2$), 22.5 (2C, (*Ar*)CH-CH-C-CH(CH$_3$)$_2$), 17.9 (2C, 2xCH$_3$-(*Ar*)C-CH-CH).

R*f* (CH$_2$Cl$_2$/CH$_3$OH 10:1) = 0.106.

ESI-MS(+): *m/z* found 1249.2995 [M-Cl]$^+$, calcd. for $C_{62}H_{73}N_6O_3Ru_2S_3^+$ 1249.2988.

Elemental analysis (%): calcd. for $C_{62}H_{73}N_6O_3Ru_2S_3Cl\cdot1.9CH_3OH\cdot0.3H_2O$ C 56.84, H 6.06, N 6.22; found C 56.86, H 6.06, N 5.91.

Synthesis of [(η^6-*p*-MeC$_6$H$_4$Pr^i)$_2$Ru$_2$(μ_2-SCH$_2$C$_6$H$_4$-*p*-Bu^t)$_2$(μ_2-SC$_6$H$_4$-*p*-CH$_2$-(C=O)-O-R]Cl (R = 6-((4-(methylamino)-1*H*-1,2,3-triazol-1-yl)methyl)pyrimidine-2,4(1*H*,3*H*)-dione) (30)

To a solution of **7** (0.250 g, 0.233 mmol, 1 equiv.) in dry DMF (10 mL) under inert atmosphere (N$_2$) at r.t were added successively **27** (0.046 g, 0.275 mmol, 1.2 equiv.), $Cu_2SO_4\cdot5H_2O$ (0.058 g, 0.232 mmol, 1 equiv.) and sodium ascorbate (0.092 g, 0.464 mmol, 2 equiv.). The reaction mixture was stirred at 50°C overnight and the reaction evolution was verified by TLC. The mixture was filtered and the filtrate was diluted with EtOAc (100 mL), washed with H_2O (2×100 mL), and saturated aq. soln. NH_4Cl. The organic phase was dried over anhydrous Na_2SO_4 and concentrated under reduced pressure. Purification by column chromatography (CH$_2$Cl$_2$/CH$_3$OH 9:1 (v/v)) afforded click product **30** as an orange solid (0.108 g, 0.088 mmol, yield 38%).

^{1}H-NMR (CDCl$_3$) δ_H, ppm: 12.51 (1H, s, N*H*-(C=O)-NH), 8.84 (1H, s br, (*Tr*)C-N=N-N-C*H*), 8.11 (1H, s, NH-(C=O)-N*H*), 7.73 (2H, d, 2xS-(*Ar*)C-CH-C*H*-C-CH$_2$, $^3J_{H,H}$ = 8.1 Hz), 7.47 (4H, m, 4xS-CH$_2$-(*Ar*)C-CH-C*H*-C-C(CH$_3$)$_3$, $^3J_{H,H}$ = 8.3 Hz), 7.40 (4H, m, 4xS-CH$_2$-(*Ar*)C-C*H*-CH-C-C(CH$_3$)$_3$, $^3J_{H,H}$ = 8.4 Hz), 7.27 (2H, d, 2xS-(*Ar*)C-C*H*-CH-C-CH$_2$, $^3J_{H,H}$ = 8.1 Hz), 5.76 (2H, s, (*Tr*)C-N=N-N-C*H$_2$*-C-CH-(C=O)), 5.54 (1H, s, (*Tr*)C-N=N-N-CH$_2$-C-C*H*-(C=O)), 5.24 (2H, s, (C=O)-O-C*H$_2$*-(*Tr*)C-N-N=N), 5.09 (2H, d, 2xCH$_3$-(*Ar*)C-CH-C*H*-C, $^3J_{H,H}$ = 5.7 Hz), 4.94 (2H, d, 2xCH$_3$-(*Ar*)C-CH-C*H*-C, $^3J_{H,H}$ = 5.9 Hz), 4.90 (2H, d, 2xCH$_3$-(*Ar*)C-C*H*-CH-C, $^3J_{H,H}$ = 5.7 Hz), 4.61 (2H, d, 2xCH$_3$-(*Ar*)C-C*H*-CH-C, $^3J_{H,H}$ = 5.8 Hz), 3.66 (2H, s, S-(*Ar*)C-CH-CH-C-C*H$_2$*-(C=O)), 3.58 (2H, s, S-C*H$_2$*-(*Ar*)C-CH-CH-C-(CH$_3$)$_3$), 3.39 (2H, s, S-C*H$_2$*-(*Ar*)C-CH-CH-C-(CH$_3$)$_3$), 1.92 (2H, sept, 2x(*Ar*)C-CH-CH-C-C*H*(CH$_3$)$_2$, $^3J_{H,H}$ = 6.9 Hz), 1.69 (6H s, 2xC*H$_3$*-(*Ar*)C-CH-CH-C), 1.37 (9H, s, S-CH$_2$-(*Ar*)C-CH-CH-C-C(C*H$_3$*)$_3$), 1.33 (9H, s, S-CH$_2$-(*Ar*)C-CH-CH-C-C(C*H$_3$*)$_3$), 0.92 (6H, d, (*Ar*)C-CH-CH-C-CH(C*H$_3$*)$_2$, $^3J_{H,H}$ = 6.9 Hz), 0.88 (6H, d, (*Ar*)C-CH-CH-C-CH(C*H$_3$*)$_2$, $^3J_{H,H}$ = 6.9 Hz).

^{13}C-NMR (CDCl$_3$) δ_C, ppm: 170.7 (1C, S-(*Ar*)C-CH-CH-C-CH$_2$-(C=O)-O), 163.9 (1C, (*Tr*)C-N=N-N-CH$_2$-C-CH-(C=O)-NH), 152.02, 151.97 (2C, 2xS-CH$_2$-(*Ar*)C-CH-CH-C-C(CH$_3$)$_3$), 150.8 (1C, (*Tr*)C-N=N-N-CH$_2$-C-CH-(C=O)-NH), 150.7 (1C, (*Tr*)C-N=N-N-CH$_2$-C-NH-(C=O)-NH), 142.6 (1C, (*Tr*)C-N=N-N-CH$_2$-C-CH-(C=O)-NH), 136.74, 136.71 (2C, 2xS-CH$_2$-(*Ar*)C-CH-CH-C-C(CH$_3$)$_3$), 136.4 (1C, S-(*Ar*)C-CH-CH-C-CH$_2$-(C=O)-O), 134.7 (1C, S-(*Ar*)C-CH-CH-C-CH$_2$-(C=O)-O), 133.0 (2C, 2xS-(*Ar*)C-CH-C*H*-C-CH$_2$-(C=O)-O), 130.1 (2C, 2xS-(*Ar*)C-C*H*-CH-C-CH$_2$-(C=O)-O), 129.4, 129.1 (4C, 4xS-CH$_2$-(*Ar*)C-CH-CH-C-C(CH$_3$)$_3$), 125.8 (1C, (*Tr*)C-N=N-N-CH), 125.8, 125.6 (4C, 4xS-CH$_2$-(*Ar*)C-CH-CH-C-C(CH$_3$)$_3$), 107.3 (2C, 2xCH$_3$-(*Ar*)C-CH-CH-C), 100.9 (1C, (*Tr*)C-N=N-N-C-CH-(C=O)), 100.6 (2C, 2xCH$_3$-(*Ar*)C-CH-CH-C, 84.1 (2C, 2xCH$_3$-(*Ar*)C-CH-CH-C), 83.86 (2C, 2xCH$_3$-(*Ar*)C-CH-CH-C), 83.83 (2C, 2xCH$_3$-(*Ar*)C-CH-CH-C), 82.5 (2C, 2xCH$_3$-(*Ar*)C-CH-CH-C), 58.9 (1C,

(C=O)-O-$\underline{C}H_2$-(*$\underline{Tr}$*)C-N=N-N), 49.5 (1C, (*Tr*)C-N=N-N-$\underline{C}H_2$-C-NH), 41.2 (1C, S-(*Ar*)C-CH-CH-C-$\underline{C}H_2$-(C=O)-O), 40.1 (1C, S-$\underline{C}H_2$-(*Ar*)C-CH-CH-C-C(CH_3)$_3$, 39.5 (1C, S-$\underline{C}H_2$-(*Ar*)C-CH-CH-C-C(CH_3)$_3$, 35.0, 34.9 (2C, 2xS-CH_2-(*Ar*)C-CH-CH-C-$\underline{C}$(CH_3)$_3$), 31.56, 31.54 (6C, 2xS-CH_2-(*Ar*)C-CH-CH-C-C($\underline{C}H_3$)$_3$), 31.0 (2C, 2x(*Ar*)CH-CH-C-$\underline{C}$H(CH_3)$_2$), 23.1 (2C, (*Ar*)CH-CH-C-CH($\underline{C}H_3$)$_2$), 22.8 (2C, (*Ar*)CH-CH-C-CH($\underline{C}H_3$)$_2$), 18.2 (2C, 2x$\underline{C}H_3$-(*Ar*)C-CH-CH).
R_f (CH_2Cl_2/CH_3OH 10:1) = 0.111.
ESI-MS(+): *m/z* found 1202.2813 [M-Cl]$^+$, calcd. for $C_{58}H_{72}N_5O_4Ru_2S_3^+$ 1202.2828.
Elemental analysis (%): calcd. for $C_{58}H_{72}N_5O_4Ru_2S_3Cl\cdot 2.4CH_3OH$ C 55.21, H 6.26, N 5.33; found C 55.22, H 6.27, N 5.28.

Synthesis of [(η^6-*p*-$MeC_6H_4Pr^i$)$_2$$Ru_2$($\mu_2$-$SCH_2C_6H_4$-*p*-$Bu^t$)$_2$($\mu_2$-$SC_6H_4$-*p*-$CH_2$-(C=O)-NH-R]Cl (R = 6-((4-(methylamino)-1*H*-1,2,3-triazol-1-yl)methyl)pyrimidine-2,4(1*H*,3*H*)-dione) (31)

To a solution of **8** (0.300 g, 0.280 mmol, 1 equiv.) in mixture of CH_3CN/H_2O 1:1 v/v (40 mL) under inert atmosphere (N_2) at r.t were added successively **27** (0.056 g, 0.336 mmol, 1.2 equiv.), $Cu_2SO_4\cdot 5H_2O$ (0.070 g, 0.280 mmol, 1 equiv.) and sodium ascorbate (0.111 g, 0.560 mmol, 2 equiv.). The mixture was stirred at 50°C overnight and the reaction evolution was verified by TLC. The reaction mixture was cooled to r.t., filtered and diluted with EtOAc (2×30 mL). The organic phase was successively washed with H_2O (2×30 mL), dried over anhydrous Na_2SO_4, filtered and concentrated under reduced pressure. Purification by column chromatography (CH_2Cl_2/CH_3OH 9:1 (v/v)) afforded **31** as an orange solid (0.111 g, 0.090 mmol, yield 32%).

^{1}H-NMR (MeOD-d_4) δ_H, ppm: 8.04 (1H, s, (*Tr*)C-N=N-N-C$\underline{H}$), 7.79 (2H, d, 2xS-(*Ar*)C-CH-C$\underline{H}$-C-CH_2-(C=O)-NH, $^3J_{H,H}$ = 8.2 Hz), 7.53 (4H, m, 4xS-CH_2-(*Ar*)C-CH-C$\underline{H}$-C-C(CH_3)$_3$, $^3J_{H,H}$ = 8.4 Hz), 7.47 (4H, m, 4xS-CH_2-(*Ar*)C-C$\underline{H}$-CH-C-C(CH_3)$_3$, $^3J_{H,H}$ = 8.4 Hz), 7.27 (2H, d, 2xS-(*Ar*)C-C$\underline{H}$-CH-C-CH_2-(C=O)-NH, $^3J_{H,H}$ = 8.2 Hz), 5.42 (2H, s, (C=O)-NH-CH_2-(*Tr*)C-N=N-N-C$\underline{H_2}$), 5.26 (2H, d, 2xCH_3-(*Ar*)C-CH-C$\underline{H}$-C, $^3J_{H,H}$ = 5.7 Hz), 5.19 (1H, s, NH-(C=O)-NH-(C=O)-C$\underline{H}$-C), 5.12 (2H, d, 2xCH_3-(*Ar*)C-CH-C$\underline{H}$-C, $^3J_{H,H}$ = 5.8 Hz), 5.05 (2H, d, 2xCH_3-(*Ar*)C-C$\underline{H}$-CH-C, $^3J_{H,H}$ = 5.7 Hz), 4.73 (2H, d, 2xCH_3-(*Ar*)C-C$\underline{H}$-CH-C, $^3J_{H,H}$ = 5.8 Hz), 4.48 (2H, s, (C=O)-NH-C$\underline{H_2}$-(*Tr*)C-N=N-N-CH_2), 3.69 (2H, s, S-C$\underline{H_2}$-(*Ar*)C-CH-CH-C-(CH_3)$_3$), 3.55 (2H, s, S-(*Ar*)C-CH-CH-C-C$\underline{H_2}$-(C=O)-NH), 3.50 (2H, s, S-C$\underline{H_2}$-(*Ar*)C-CH-CH-C-(CH_3)$_3$), 1.87 (2H, sept, 2x(*Ar*)C-CH-CH-C-C$\underline{H}$(CH_3)$_2$, $^3J_{H,H}$ = 6.9 Hz), 1.76 (6H s, 2xC$\underline{H}_3$-(*Ar*)C-CH-CH-C), 1.38 (9H, s, S-CH_2-(*Ar*)C-CH-CH-C-C(C$\underline{H_3}$)$_3$), 1.35 (9H, s, S-CH_2-(*Ar*)C-CH-CH-C-C(C$\underline{H_3}$)$_3$), 0.91 (6H, d, 2x(*Ar*)C-CH-CH-C-CH(C$\underline{H_3}$)$_2$, $^3J_{H,H}$ = 6.9 Hz), 0.88 (6H, d, 2x(*Ar*)C-CH-CH-C-CH(C$\underline{H_3}$)$_2$, $^3J_{H,H}$ = 6.9 Hz).

^{13}C-NMR (MeOD-d_4) δ_C, ppm: 173.4 (1C, S-(*Ar*)C-CH-CH-C-CH_2-($\underline{C}$=O)-NH), 166.5 (1C, NH-(C=O)-NH-($\underline{C}$=O)-CH-C), 153.2 (1C, NH-(C=O)-NH-(C=O)-CH-$\underline{C}$), 152.7, 152.6 (2C, 2xS-CH_2-(*Ar*)C-CH-CH-$\underline{C}$-C(CH_3)$_3$), 152.4 (1C, NH-($\underline{C}$=O)-NH-(C=O)-CH-C), 146.6 (1C, (*Tr*)$\underline{C}$-N=N-N-CH), 138.6, 138.5 (2C, 2xS-CH_2-(*Ar*)$\underline{C}$-CH-CH-C-C(CH_3)$_3$), 137.8 (1C, S-(*Ar*)$\underline{C}$-CH-CH-C-CH_2-(C=O)-NH), 137.3 (1C, S-(*Ar*)C-CH-CH-$\underline{C}$-CH_2-(C=O)-NH), 134.3 (2C, 2xS-(*Ar*)C-CH-$\underline{C}$H-C-CH_2-(C=O)-NH), 130.7 (2C, 2xS-(*Ar*)C-$\underline{C}$H-CH-C-CH_2-(C=O)-NH), 130.5, 130.3 (4C, 4xS-CH_2-(*Ar*)C-$\underline{C}$H-CH-C-C(CH_3)$_3$), 126.7, 126.6 (4C, 4xS-CH_2-(*Ar*)C-CH-$\underline{C}$H-C-C(CH_3)$_3$), 125.5 (1C, (*Tr*)C-N=N-N-$\underline{C}$H), 107.9 (2C, 2xCH_3-(*Ar*)C-CH-CH-$\underline{C}$), 102.5 (2C, 2xCH_3-(*Ar*)$\underline{C}$-CH-CH-C), 100.2 (1C, NH-(C=O)-NH-(C=O)-$\underline{C}$H-C), 86.0 (2C, 2xCH_3-(*Ar*)C-CH-$\underline{C}$H-C), 84.55 (2C, 2xCH_3-(*Ar*)C-$\underline{C}$H-CH-C), 84.52 (2C, 2xCH_3-(*Ar*)C-CH-$\underline{C}$H-C), 83.9 (2C, 2xCH_3-(*Ar*)C-$\underline{C}$H-CH-C), 50.5 (1C, (C=O)-NH-CH_2-(*Tr*)C-N=N-N-$\underline{C}H_2$), 43.2 (1C, S-(*Ar*)C-CH-CH-C-$\underline{C}H_2$-(C=O)-NH), 41.2 (1C, S-$\underline{C}H_2$-(*Ar*)C-CH-CH-C-C(CH_3)$_3$), 40.7 (1C, S-$\underline{C}H_2$-(*Ar*)C-CH-CH-C-C(CH_3)$_3$), 35.8 (1C, (C=O)-NH-$\underline{C}H_2$-(*Tr*)C-N=N-N-CH_2), 35.62, 35.57 (2C, 2xS-CH_2-(*Ar*)C-CH-CH-C-$\underline{C}$(CH_3)$_3$), 32.0 (2C, 2x(*Ar*)CH-CH-C-$\underline{C}$H(CH_3)$_2$), 31.85, 31.82

(6C, 2xS-CH2-(*Ar*)C-CH-CH-C-C($\underline{C}H_3$)3), 23.6 (2C, (*Ar*)CH-CH-C-CH($\underline{C}H_3$)2), 22.9 (2C, (*Ar*)CH-CH-C-CH($\underline{C}H_3$)2), 18.2 (2C, 2x$\underline{C}H_3$-(*Ar*)C-CH-CH).
R*f* (CH_2Cl_2/CH_3OH 10:1) = 0.268.
ESI-MS(+): *m/z* found 1201.2973 $[M-Cl]^+$, calcd. for $C_{58}H_{73}N_6O_3Ru_2S_3^+$ 1201.2988.
Elemental analysis (%): calcd. for $C_{58}H_{73}ClN_6O_3Ru_2S_3\cdot 7.5CH_2Cl_2\cdot 15H_2O$ C 36.71, H 5.55, N 3.92; found: C 36.35, H 5.01, N 4.08.

Synthesis of 9-(2-methanesulfonate ethyl)adenine (2-(6-amino-9*H*-purin-9-yl)ethyl methanesulfonate) (32A) and 9-(2-azidoethyl)adenine (9-(2-azidoethyl)-9*H*-purin-6-amine) (32)

To a solution of 9-(2-hydroxyethyl)adenine (2-(6-amino-9*H*-purin-9-yl)ethan-1-ol) (0.200 g, 1.116 mmol, 1 equiv.) in dry DMF (50 mL) at 0°C under inert atmosphere (N_2), were added successively MsCl (0.22 mL, 2.790 mmol, 2.5 equiv.), and TEA (0.39 mL, 2.790 mmol, 2.5 equiv.). The mixture was further stirred at 0°C for 3 h and then at r.t. overnight. The reaction evolution was verified by TLC, the mixture was concentrated under reduced pressure. The obtained mesylate **32A** was solubilized in dry DMF (20 mL) under inert atmosphere (N_2), NaN_3 (0.145 g, 2.232 mmol, 2 equiv.) was added and the mixture was stirred at 60°C for 48 h. A second portion of NaN_3 (0.145 g, 2.232 mmol, 2 equiv.) was added and the reaction mixture was further stirred for 72 h. The reaction evolution was verified by TLC; the mixture was filtrated and the filtrate was diluted with CH_3OH (5 mL) and hexane (5 mL) and concentrated under reduced pressure. The residue was suspended in CH_2Cl_2 (30 mL) and filtered. The precipitate was solubilized in CH_3OH (30 mL) and concentrated under reduced pressure to afford **32** as a white solid (0.214 g, 1.046 mmol, yield 94%) which was used without further purification.

32A:
^{1}H-NMR (MeOD-*d4*) δ*H*, ppm: 8.24 (1H, s, N-C$\underline{H}$-N-C-NH2), 8.16 (1H, s, N-C$\underline{H}$-N-C-C-NH2), 4.34 (2H, t, N-C$\underline{H_2}$-CH2-O-(SO2)-CH3, $^3J_{H,H}$ = 5.4 Hz), 3.90 (2H, t, N-CH2-C$\underline{H_2}$-O-(SO2)-CH3, $^3J_{H,H}$ = 5.4 Hz), 2.86 (3H, s, N-CH2-CH2-O-(SO2)-C$\underline{H_3}$).
ESI-MS(+): *m/z* found 258.0662 $[M+H]^+$, calcd. for $C_8H_{12}N_5O_3S^+$ 258.0655.

32:
^{1}H-NMR (MeOD-*d4*) δ*H*, ppm: 8.22 (1H, s, N-C$\underline{H}$-N-C-NH2), 8.16 (1H, s, N-C$\underline{H}$-N-C-C-NH2), 4.42 (2H, t, N-C$\underline{H_2}$-CH2-N3, $^3J_{H,H}$ = 5.8 Hz), 3.80 (2H, t, N-CH2-C$\underline{H_2}$-N3, $^3J_{H,H}$ = 5.8 Hz).
^{13}C-NMR (MeOD-*d4*) δ*C*, ppm: 157.5 (1C, N-CH-N-C-$\underline{C}$-NH2), 153.7 (1C, N-$\underline{C}$H-N-C-NH2), 150.8 (1C, N-$\underline{C}$-N-CH2-CH2-N3), 143.0 (1C, CH2-N-$\underline{C}$H-N-C-C-NH2), 120.2 (1C, N-CH-N-$\underline{C}$-C-NH2), 51.4 (1C, N-C-N-$\underline{C}$H2-CH2-N3), 44.3 (1C, N-C-N-CH2-$\underline{C}$H2-N3).
R*f* (CH_2Cl_2/CH_3OH 10:1) = 0.260.
ESI-MS(+): *m/z* found 205.0944 $[M+H]^+$, calcd. for $C_7H_9N_8^+$ 205.0945.

Synthesis of $[(\eta^6\text{-}p\text{-MeC}_6H_4Pr^i)_2Ru_2(\mu_2\text{-SCH}_2C_6H_4\text{-}p\text{-Bu}^t)_2(\mu_2\text{-SC}_6H_4\text{-}p\text{-CH}_2\text{-(C=O)-NH-R})]Cl$ (R = 9-(2-(4-(methylamino)-1*H*-1,2,3-triazol-1-yl)ethyl)-9*H*-purin-6-amine) (33)

To a solution of **8** (0.228 g, 0.213 mmol, 1 equiv.) and **31** (0.130 g, 0.639 mmol, 3 equiv.) in dry DMF (10 mL), were added successively $CuSO_4\cdot 5H_2O$ (0.053 g, 0.213 mmol, 1 equiv.) and sodium ascorbate (0.084 g, 0.426 mmol, 2 equiv.). The reaction mixture was stirred at 60°C under inert atmosphere (N_2) for further 24 h and the reaction evolution was verified by TLC. The reaction mixture was solubilised in EtOAc (100 mL) and washed with H_2O (2×100 mL); the unified aqueous phases were further washed with EtOAc (100 mL). The combined organic phases were washed with brine (100 mL), dried over anhydrous Na_2SO_4, filtered and concentrated to dryness under reduced pressure. Purification by column

chromatography (CH_2Cl_2/CH_3OH 9:1 (v/v)) afforded **33** as an orange solid (0.035 g, 0.027 mmol, yield 13%).

^{1}H-NMR (DMSO-*d*$_6$) δ$_H$, ppm: 8.55 (1H, t, S-(*Ar*)C-CH-CH-C-CH$_2$-(C=O)-N*H*, $^3J_{H,H}$ = 5.6 Hz), 8.13 (1H, s, N-C*H*-N-C-NH$_2$), 7.89 (1H, s, N-C*H*-N-C-C-NH$_2$), 7.82 (1H, s, (*Tr*)C-N=N-N-C*H*), 7.69 (2H, d, 2xS-(*Ar*)C-CH-C*H*-C-CH$_2$-(C=O)-NH, $^3J_{H,H}$ = 8.1 Hz), 7.50 (4H, m, 4xS-CH$_2$-(*Ar*)C-CH-C*H*-C-C(CH$_3$)$_3$, $^3J_{H,H}$ = 8.7 Hz), 7.42 (4H, m, 4xS-CH$_2$-(*Ar*)C-C*H*-CH-C-C(CH$_3$)$_3$, $^3J_{H,H}$ = 8.4 Hz), 7.22 (2H, d, 2xS-(*Ar*)C-C*H*-CH-C-CH$_2$-(C=O)-NH, $^3J_{H,H}$ = 7.1 Hz), 7.21 (2H, s br, N-CH-N-C-N*H$_2$*), 5.36 (2H, d, 2xCH$_3$-(*Ar*)C-CH-C*H*-C, $^3J_{H,H}$ = 5.7 Hz), 5.24 (4H, d, 2xCH$_3$-(*Ar*)C-C*H*-CH-C, 2xCH$_3$-(*Ar*)C-CH-C*H*-C, $^3J_{H,H}$ = 6.1 Hz), 4.84 (2H, t, (*Tr*)C-N=N-N-C*H$_2$*-CH$_2$-N, $^3J_{H,H}$ = 5.6 Hz), 4.68 (2H, d, 2xCH$_3$-(*Ar*)C-C*H*-CH-C, $^3J_{H,H}$ = 5.8 Hz), 4.63 (2H, t, (*Tr*)C-N=N-N-CH$_2$-C*H$_2$*-N, $^3J_{H,H}$ = 5.7 Hz), 4.24 (2H, d, (C=O)-NH-C*H$_2$*-(*Tr*)C-N=N-N-CH$_2$, $^3J_{H,H}$ = 5.5 Hz), 3.63 (2H, s, S-C*H$_2$*-(*Ar*)C-CH-CH-C-(CH$_3$)$_3$), 3.44 (2H, s, S-C*H$_2$*-(*Ar*)C-CH-CH-C-(CH$_3$)$_3$), 3.42 (2H, s, S-(*Ar*)C-CH-CH-C-C*H$_2$*-(C=O)-NH), 1.71-1.79 (2H, m, 2x(*Ar*)C-CH-CH-C-C*H*(CH$_3$)$_2$), 1.75 (6H s, 2xC*H$_3$*-(*Ar*)C-CH-CH-C), 1.33 (9H, s, S-CH$_2$-(*Ar*)C-CH-CH-C-C(C*H$_3$*)$_3$), 1.29 (9H, s, S-CH$_2$-(*Ar*)C-CH-CH-C-C(C*H$_3$*)$_3$), 0.78 (6H, d, 2x(*Ar*)C-CH-CH-C-CH(C*H$_3$*)$_2$, $^3J_{H,H}$ = 6.8 Hz), 0.74 (6H, d, 2x(*Ar*)C-CH-CH-C-CH(C*H$_3$*)$_2$, $^3J_{H,H}$ = 6.8 Hz).

^{13}C-NMR (DMSO-*d*$_6$) δ$_C$, ppm: 169.6 (1C, S-(*Ar*)C-CH-CH-C-CH$_2$-(*C*=O)-NH), 155.9 (1C, N-CH-N-C-*C*-NH$_2$), 152.5 (1C, N-*C*H-N-C-NH$_2$), 150.5, 150.4 (2C, 2xS-CH$_2$-(*Ar*)C-CH-CH-*C*-C(CH$_3$)$_3$), 149.5 (1C, (*Tr*)N-N=N-(CH$_2$)$_2$-N-*C*-N)), 144.7 (1C, (*Tr*)*C*-N=N-N-CH), 140.5 (1C, N-*C*H-N-C-C-NH$_2$), 137.0, 136.8 (2C, 2xS-CH$_2$-(*Ar*)*C*-CH-CH-C-C(CH$_3$)$_3$), 136.1 (1C, S-(*Ar*)*C*-CH-CH-C-CH$_2$-(C=O)-NH), 135.2 (1C, S-(*Ar*)C-CH-CH-*C*-CH$_2$-(C=O)-NH), 132.2 (2C, 2xS-(*Ar*)C-CH-*C*H-C-CH$_2$-(C=O)-NH), 129.29, 129.24 (4C, 4xS-CH$_2$-(*Ar*)C-CH-*C*H-C-C(CH$_3$)$_3$), 128.9 (2C, 2xS-(*Ar*)C-*C*H-CH-C-CH$_2$-(C=O)-NH), 125.2, 125.0 (4C, 4xS-CH$_2$-(*Ar*)C-*C*H-CH-C-C(CH$_3$)$_3$), 123.1 (1C, (*Tr*)C-N=N-N-*C*H), 118.6 (1C, N-CH-N-*C*-C-NH$_2$), 105.2 (2C, 2xCH$_3$-(*Ar*)C-CH-CH-*C*), 101.4 (2C, 2xCH$_3$-(*Ar*)*C*-CH-CH-C), 85.2 (2C, 2xCH$_3$-(*Ar*)C-CH-*C*H-C, 82.6 (2C, 2xCH$_3$-(*Ar*)C-*C*H-CH-C), 82.4 (2C, 2xCH$_3$-(*Ar*)C-CH-*C*H-C), 82.3 (2C, 2xCH$_3$-(*Ar*)C-*C*H-CH-C), 48.4 (1C, (C=O)-NH-CH$_2$-(*Tr*)C-N=N-N-CH$_2$-*C*H$_2$), 42.9 (1C, (C=O)-NH-CH$_2$-(*Tr*)C-N=N-N-*C*H$_2$-CH$_2$), 41.6 (1C, S-(*Ar*)C-CH-CH-C-*C*H$_2$-(C=O)-NH), 39.5 (1C, S-*C*H$_2$-(*Ar*)C-CH-CH-C-C(CH$_3$)$_3$), 39.3 (1C, S-*C*H$_2$-(*Ar*)C-CH-CH-C-C(CH$_3$)$_3$), 34.40, 34.34 (2C, 2xS-CH$_2$-(*Ar*)C-CH-CH-C-*C*(CH$_3$)$_3$), 34.2 (1C, (C=O)-NH-*C*H$_2$-(*Tr*)C-N=N-N-CH$_2$), 31.15, 31.13 (6C, 2xS-CH$_2$-(*Ar*)C-CH-CH-C-C(*C*H$_3$)$_3$), 29.9 (2C, 2x(*Ar*)CH-CH-C-*C*H(CH$_3$)$_2$), 23.0 (2C, (*Ar*)CH-CH-C-CH(*C*H$_3$)$_2$), 21.7 (2C, (*Ar*)CH-CH-C-CH(*C*H$_3$)$_2$), 17.5 (2C, 2x*C*H$_3$-(*Ar*)C-CH-CH).

R*$_f$* (CH_2Cl_2/CH_3OH 9:1) = 0.088.

ESI-MS(+): *m/z* found 1238.3424 [M-Cl]$^+$, calcd. for $C_{60}H_{76}N_9ORu_2S_3^+$ 1238.3416.

Elemental analysis (%): calcd. for $C_{60}H_{76}ClN_9ORu_2S_3 \cdot 0.1CH_2Cl_2 \cdot 1.5CH_3OH$ C 55.64, H 6.23, N 9.48; found C 55.65, H 6.21, N 9.28.

7. Synthesis of compounds **34-39** (family 5)

Synthesis of [(η^6-*p*-MeC$_6$H$_4$Pr^i)$_2$Ru$_2$(μ_2-SCH$_2$C$_6$H$_4$-*p*-Bu^t)$_2$(μ_2-SC$_6$H$_4$-*o*-R)]Cl (R = 1-methylene-1*H*-1,2,3-triazole-4-phenyl) (34)

To a solution of **9** (0.200 g, 0.194 mmol, 1 equiv.) in a mixture CH_3CN/H_2O (20 mL, 1:1 (v/v)) under inert atmosphere (N_2) at room temperature were added successively ethynylbenzene (0.032 mL, 0.292 mmol, 1.5 equiv.), $Cu_2SO_4 \cdot 5H_2O$ (0.049 g, 0.194 g, 1 equiv.) and sodium ascorbate (0.077 g, 0.388 mmol, 2 equiv.). The mixture was stirred at 50°C for 24 h and the reaction evolution was verified by TLC. The mixture was diluted with EtOAc (100 mL), washed with H_2O (100 mL), saturated aq. soln. NH_4Cl, dried over anhydrous Na_2SO_4, filtered and concentrated under reduced pressure. Purification by column chromatography (CH_2Cl_2/CH_3OH 9.5:0.5 (v/v)) afforded **34** as an orange solid (0.198 g, 0.175 mmol, yield 90%).

^{1}H-NMR ($CDCl_3$) δ_H, ppm: 9.40 (1H, s, (*Tr*)N-N=N-C-C*H*), 8.01 (2H, 2x(*Tr*)N-N=N-C-(*Ar*)C-C*H*-CH-CH, $^3J_{H,H}$ = 7.4 Hz), 7.74 (1H, d, S-(*Ar*)C-C*H*-CH-CH-CH, $^3J_{H,H}$ = 7.6 Hz), 7.36-7.46 (10H, m, 4xS-CH_2-(*Ar*)C-CH-C*H*-C-C(CH_3)$_3$, 4xS-CH_2-(*Ar*)C-C*H*-CH-C-C(CH_3)$_3$, 2x(*Tr*)N-N=N-C-(*Ar*)C-CH-C*H*-CH), 7.20-7.36 (4H, m, S-(*Ar*)C-CH-C*H*-CH-CH, S-(*Ar*)C-CH-CH-C*H*-CH, S-(*Ar*)C-CH-CH-CH-C*H*, (*Tr*)N-N=N-C-(*Ar*)C-CH-CH-C*H*), 6.41 (2H, s, S-(*Ar*)C-C-C*H_2*-N), 5.03 (2H, d, 2xCH_3-(*Ar*)C-CH-C*H*-C, $^3J_{H,H}$ = 5.5 Hz), 4.98 (2H, d, 2xCH_3-(*Ar*)C-CH-C*H*-C, $^3J_{H,H}$ = 5.2 Hz), 4.97 (2H, d, 2xCH_3-(*Ar*)C-C*H*-CH-C, $^3J_{H,H}$ = 5.5 Hz), 4.59 (2H, d, 2xCH_3-(*Ar*)C-C*H*-CH-C, $^3J_{H,H}$ = 5.7 Hz), 3.61 (2H, s, C*H_2*-(*Ar*)C-CH-CH-C-C(CH_3)$_3$), 3.33 (2H, s, C*H_2*-(*Ar*)C-CH-CH-C-C(CH_3)$_3$), 1.82 (2H, sept, 2x(*Ar*)C-CH-CH-C-C*H*(CH_3)$_2$, $^3J_{H,H}$ = 6.8 Hz), 1.57 (6H, s, 2xC*H_3*-(*Ar*)C-CH-CH-C), 1.34 (9H, s, S-CH_2-(*Ar*)C-CH-CH-C-C(C*H_3*)$_3$), 1.29 (9H, s, S-CH_2-(*Ar*)C-CH-CH-C-C(C*H_3*)$_3$), 0.91 (6H, d, (*Ar*)C-CH-CH-C-CH(C*H_3*)$_2$, $^3J_{H,H}$ = 6.8 Hz), 0.84 (6H, d, (*Ar*)C-CH-CH-C-CH(C*H_3*)$_2$, $^3J_{H,H}$ = 6.9 Hz).

^{13}C-NMR ($CDCl_3$) δ_C, ppm: 151.8, 151.2 (2C, 2xS-CH_2-(*Ar*)C-CH-CH-*C*-C(CH_3)$_3$), 146.8 (1C, (*Tr*)N-N=N-*C*-(*Ar*)C-CH-CH-CH), 139.7 (1C, S-(*Ar*)*C*-C-CH-CH-CH-CH), 138.6 (1C, S-(*Ar*)C-*C*-CH-CH-CH-CH), 137.0, 136.6 (2C, 2xS-CH_2-(*Ar*)*C*-CH-CH-C-C(CH_3)$_3$), 134.1 (1C, S-(*Ar*)C-*C*H-CH-CH-CH), 131.6 (1C, S-(*Ar*)C-CH-CH-CH-*C*H), 131.1 (1C, (*Tr*)N-N=N-C-(*Ar*)*C*-CH-CH), 129.9 (2C, 2x(*Tr*)N-N=N-C-(*Ar*)C-CH-*C*H-CH), 129.0 (4C, 4xS-CH_2-(*Ar*)C-CH-*C*H-C-C(CH_3)$_3$), 129.0 (1C, S-(*Ar*)C-CH-CH-*C*H-CH), 128.9 (1C, S-(*Ar*)C-CH-*C*H-CH-CH), 128.0 (1C, (*Tr*)N-N=N-C-(*Ar*)C-CH-CH-*C*H), 126.0 (2C, 2x(*Tr*)N-N=N-C-(*Ar*)C-*C*H-CH-CH), 125.6, 125.5 (4C, 4xS-CH_2-(*Ar*)C-*C*H-CH-C-C(CH_3)$_3$), 124.3 (1C, (*Tr*)N-N=N-C-*C*H), 106.8 (2C, 2xCH_3-(*Ar*)C-CH-CH-*C*), 101.3 (2C, 2xCH_3-(*Ar*)*C*-CH-CH-C), 83.8 (2C, 2xCH_3-(*Ar*)C-CH-*C*H-C), 83.6 (2C, 2xCH_3-(*Ar*)C-*C*H-CH-C), 83.3 (2C, 2xCH_3-(*Ar*)C-CH-*C*H-C), 82.7 (2C, 2xCH_3-(*Ar*)C-*C*H-CH-C), 54.0 (1C, s, S-(*Ar*)C-C-*C*H_2-(*Tr*)N-N=N-C),), 40.6 (1C, S-*C*H_2-(*Ar*)C-CH-CH-C-C(CH_3)$_3$), 39.5 (1C, S-*C*H_2-(*Ar*)C-CH-CH-C-C(CH_3)$_3$), 34.9, 34.8 (2C, 2xS-CH_2-(*Ar*)C-CH-CH-C-*C*(CH_3)$_3$), 31.6 (6C, 2xS-CH_2-(*Ar*)C-CH-CH-C-C(*C*H_3)$_3$), 30.8 (2C, 2x(*Ar*)CH-CH-C-*C*H(CH_3)$_2$), 23.0 (2C, (*Ar*)CH-CH-C-CH(*C*H_3)$_2$), 22.8 (2C, (*Ar*)CH-CH-C-CH(*C*H_3)$_2$), 18.1 (2C, 2x*C*H_3-(*Ar*)C-CH-CH).

R_f (CH_2Cl_2/MeOH 9.5:0.5) = 0.339.

ESI-MS(+): *m/z* found 1096.2841 [M-Cl]$^+$, calcd. for $C_{57}H_{70}N_3Ru_2S_3^+$ 1096.2813.

Elemental analysis (%): calcd. for $C_{57}H_{70}N_3Ru_2S_3\cdot0.6CH_2Cl_2\cdot CH_3OH$ C 57.98, H 6.24, N 3.46, found C 57.98, H 6.24, N 4.06.

Synthesis of [(η^6-*p*-$MeC_6H_4Pr^i$)$_2Ru_2$(μ_2-$SCH_2C_6H_4$-*p*-Bu^t)$_2$(μ_2-SC_6H_4-*o*-R)]Cl (R = (1-methylene-1*H*-1,2,3-triazol-4-yl)(4-(phenyl)methanol)) (35)

To a solution of **9** (0.250 g, 0.242 mmol, 1 equiv.) in dry DMF (10 mL) under inert atmosphere (N_2) at r.t were added successively 4-ethynylbenzyl alcohol (0.038 g, 0.287 mmol, 1.2 equiv.), $Cu_2SO_4\cdot5H_2O$ (0.060 g, 0.240 mmol, 1 equiv.) and sodium ascorbate (0.096 g, 0.484 mmol, 2 equiv.). The mixture was stirred at 50°C for 24 h and the reaction evolution was verified by TLC. The mixture was cooled to r.t., filtered and the filtrate was diluted with EtOAc (100 mL). The organic phase was successively washed with H_2O (2×100 mL) and saturated aq. soln. NH_4Cl (100 mL). The organic phase was dried over anhydrous Na_2SO_4, filtered and concentrated to dryness under reduced pressure. Purification by column chromatography (CH_2Cl_2/CH_3OH 10:1 (v/v)), followed by a second purification on analytical TLC plates (CH_2Cl_2/CH_3OH 9.5:0.5 (v/v)) afforded click product **35** as an orange solid (0.017 g, 0.015 mmol, yield 6%).

^{1}H-NMR ($CDCl_3$) δ_H, ppm: 9.36 (1H, s, (*Tr*)N-N=N-C-C*H*), 7.88 (2H, d, 2x(*Tr*)N-N=N-C-(*Ar*)C-C*H*-CH-C, $^3J_{H,H}$ = 8.0 Hz), 7.72 (1H, dd, S-(*Ar*)C-C*H*-CH-CH-CH-C, $^3J_{H,H}$ = 7.7 Hz, $^4J_{H,H}$ = 0.8 Hz), 7.45 (2H, d, 2xS-CH_2-(*Ar*)C-CH-C*H*-C-C(CH_3)$_3$, $^3J_{H,H}$ = 8.2 Hz), 7.36-7.42 (5H, m, 2xS-CH_2-(*Ar*)C-C*H*-CH-C-C(CH_3)$_3$, 2xS-CH_2-(*Ar*)C-CH-C*H*-C-C(CH_3)$_3$, S-(*Ar*)C-CH-CH-CH-C*H*-C), 7.35 (2C, d,

2x(*Tr*)N-N=N-C-(*Ar*)C-CH-C*H*-C, $^3J_{H,H}$ = 8.2 Hz), 7.31 (1H, td, S-(*Ar*)C-CH-CH-C*H*-CH-C, $^3J_{H,H}$ = 7.6 Hz, $^4J_{H,H}$ = 0.9 Hz), 7.20-7.26 (3H, S-(*Ar*)C-CH-C*H*-CH-CH-C, 2xS-CH$_2$-(*Ar*)C-C*H*-CH-C-C(CH$_3$)$_3$, $^3J_{H,H}$ = 8.2 Hz, $^4J_{H,H}$ = 1.3 Hz), 6.42 (2H, s, S-(*Ar*)C-C-C*H$_2$*-(*Tr*)N-N=N), 5.02 (2H, d, CH$_3$-(*Ar*)C-CH-C*H*-C, $^3J_{H,H}$ = 5.7 Hz), 4.97 (2H, d, CH$_3$-(*Ar*)C-CH-C*H*-C, $^3J_{H,H}$ = 6.4 Hz), 4.95 (2H, d, CH$_3$-(*Ar*)C-C*H*-CH-C, $^3J_{H,H}$ = 6.3 Hz), 4.70 (2H, s, (*Tr*)N-N=N-C-(*Ar*)C-CH-CH-C-C*H$_2$*-OH), 4.57 (2H, d, CH$_3$-(*Ar*)C-C*H*-CH-C, $^3J_{H,H}$ = 5.9 Hz), 3.59 (2H, s, S-C*H$_2$*-(*Ar*)C-CH-CH-C-(CH$_3$)$_3$), 3.32 (2H, s, S-C*H$_2$*-(*Ar*)C-CH-CH-C-(CH$_3$)$_3$), 1.80 (2H, sept, 2x(*Ar*)C-CH-CH-C-C*H*(CH$_3$)$_2$, $^3J_{H,H}$ = 6.9 Hz), 1.55 (6H, s, 2xC*H$_3$*-(*Ar*)C-CH-CH-C), 1.32 (9H, s, S-CH$_2$-(*Ar*)C-CH-CH-C-C(C*H$_3$*)$_3$), 1.27 (9H, s, S-CH$_2$-(*Ar*)C-CH-CH-C-C(C*H$_3$*)$_3$), 0.89 (6H, d, (*Ar*)C-CH-CH-C-CH(C*H$_3$*)$_2$, $^3J_{H,H}$ = 6.9 Hz), 0.82 (6H, d, (*Ar*)C-CH-CH-C-CH(C*H$_3$*)$_2$, $^3J_{H,H}$ = 6.9 Hz).

^{13}C-NMR (CDCl$_3$) δ$_C$, ppm: 151.7, 151.2 (2C, 2xS-CH$_2$-(*Ar*)C-CH-CH-*C*-C(CH$_3$)$_3$), 146.6 (1C, (*Tr*)N-N=N-*C*-(*Ar*)C-CH-CH-C), 141.3 (1C, (*Tr*)N-N=N-C-(*Ar*)C-CH-CH-*C*-CH$_2$), 139.7 (1C, S-(*Ar*)*C*-C-CH-CH-CH-CH), 138.6 (1C, S-(*Ar*)C-*C*-CH-CH-CH-CH), 136.9, 136.6 (2C, 2xS-CH$_2$-(*Ar*)*C*-CH-CH-C-C(CH$_3$)$_3$), 134.1 (1C, S-(*Ar*)C-*C*H-CH-CH-CH), 131.5 (1C, S-(*Ar*)C-CH-CH-CH-*C*H), 130.1 (1C, (*Tr*)N-N=N-C-(*Ar*)*C*-CH-CH-C-CH$_2$), 129.9 (2C, 2xS-CH$_2$-(*Ar*)C-CH-*C*H-C-C(CH$_3$)$_3$), 129.02 (2C, 2xS-CH$_2$-(*Ar*)C-CH-*C*H-C-C(CH$_3$)$_3$), 128.95 (1C, S-(*Ar*)C-CH-CH-*C*H-CH), 128.90 (1C, S-(*Ar*)C-CH-*C*H-CH-CH), 127.6 (2C, 2x(*Tr*)N-N=N-C-(*Ar*)C-CH-*C*H-C-CH$_2$), 125.9 (2C, 2x(*Tr*)N-N=N-C-(*Ar*)C-*C*H-CH-C-CH$_2$), 125.6, 125.5 (4C, 4xS-CH$_2$-(*Ar*)C-*C*H-CH-C-C(CH$_3$)$_3$), 124.1 (1C, (*Tr*)N-N=N-C-*C*H), 106.6 (2C, 2xCH$_3$-(*Ar*)C-CH-CH-*C*), 101.3 (2C, 2xCH$_3$-(*Ar*)*C*-CH-CH-C), 83.7 (2C, 2xCH$_3$-(*Ar*)C-CH-*C*H-C), 83.5 (2C, 2xCH$_3$-(*Ar*)C-*C*H-CH-C), 83.3 (2C, 2xCH$_3$-(*Ar*)C-CH-*C*H-C), 82.7 (2C, 2xCH$_3$-(*Ar*)C-*C*H-CH-C), 64.8 (1C, (*Ar*)C-CH-CH-C-*C*H$_2$-OH), 53.9 (1C, S-(*Ar*)C-C-*C*H$_2$-(*Tr*)N-N=N-C), 40.5 (1C, S-*C*H$_2$-(*Ar*)C-CH-CH-C-C(CH$_3$)$_3$), 39.4 (1C, S-*C*H$_2$-(*Ar*)C-CH-CH-C-C(CH$_3$)$_3$), 34.86, 34.76 (2C, 2xS-CH$_2$-(*Ar*)C-CH-CH-C-*C*(CH$_3$)$_3$), 31.5 (6C, 2xS-CH$_2$-(*Ar*)C-CH-CH-C-C(*C*H$_3$)$_3$), 30.8 (2C, 2x(*Ar*)CH-CH-C-*C*H(CH$_3$)$_2$), 23.0 (2C, (*Ar*)CH-CH-C-CH(*C*H$_3$)$_2$), 22.7 (2C, (*Ar*)CH-CH-C-CH(*C*H$_3$)$_2$), 18.1 (2C, 2x*C*H$_3$-(*Ar*)C-CH-CH).

ESI-MS(+): *m/z* found 1126.2928 [M-Cl]$^+$, calcd. for $C_{58}H_{72}N_3ORu_2S_3^+$ 1126.2919.

Elemental analysis (%): calcd. for $C_{58}H_{72}N_3ORu_2S_3Cl{\cdot}0.75C_3H_7NO$ C 59.52, H 6.40, N 4.32; found C 59.79, H 7.21, N 4.39.

Synthesis of [(η^6-*p*-MeC$_6$H$_4$Pr^i)$_2$Ru$_2$(μ_2-SCH$_2$C$_6$H$_4$-*p*-Bu^t)$_2$(μ_2-SC$_6$H$_4$-*o*-CH$_2$R)]Cl (R = (1-methylene-1*H*-1,2,3-triazol-4-yl)methanol) (36)

To a solution of **9** (0.250 g, 0.242 mmol, 1 equiv.) in dry DMF (10 mL) under inert atmosphere (N_2) at r.t were added successively propargyl alcohol (0.016 mL, 0.285 mmol, 1.2 equiv.), $Cu_2SO_4{\cdot}5H_2O$ (0.060 g, 0.240 mmol, 1 equiv.) and sodium ascorbate (0.096 g, 0.484 mmol, 2 equiv.). The mixture was stirred at 50°C for 24 h and the reaction evolution was verified by TLC. The reaction mixture was cooled to r.t., filtered and diluted with EtOAc (100 mL). The organic phase was successively washed with H_2O (2×100 mL), saturated aq. soln. NH_4Cl (100 mL), dried over anhydrous Na_2SO_4, filtered and concentrated under reduced pressure to dryness. Purification by column chromatography (CH_2Cl_2/CH_3OH 10:1 (v/v)) afforded **36** as an orange solid (0.051 g, 0.047 mmol, yield 19%).

^{1}H-NMR (CDCl$_3$) δ$_H$, ppm: 8.70 (1H, s, (*Tr*)N-N=N-C-C*H*), 7.76 (1H, dd, S-(*Ar*)C-C*H*-CH-CH-CH, $^3J_{H,H}$ = 7.7 Hz, $^4J_{H,H}$ = 0.9 Hz), 7.60 (2H, d, 2xS-CH$_2$-(*Ar*)C-C*H*-CH-C-C(CH$_3$)$_3$, $^3J_{H,H}$ = 8.0 Hz), 7.48 (2H, d, 2xS-CH$_2$-(*Ar*)C-CH-C*H*-C-C(CH$_3$)$_3$, $^3J_{H,H}$ = 8.2 Hz), 7.41 (4H, m, 2xS-CH$_2$-(*Ar*)C-C*H*-CH-C-C(CH$_3$)$_3$, 2xS-CH$_2$-(*Ar*)C-CH-C*H*-C-C(CH$_3$)$_3$, $^3J_{H,H}$ = 8.6 Hz), 7.37 (1H, d, S-(*Ar*)C-CH-CH-CH-C*H*, $^3J_{H,H}$ = 7.6 Hz), 7.31 (1H, td, S-(*Ar*)C-CH-CH-C*H*-CH, $^3J_{H,H}$ = 7.6 Hz), 7.23 (1H, td, S-(*Ar*)C-CH-C*H*-CH-CH, $^3J_{H,H}$ = 7.5 Hz, $^4J_{H,H}$ = 1.5 Hz), 6.16 (2H, s br, S-(*Ar*)C-C-C*H$_2$*-(*Tr*)N-N=N-C), 4.99 (2H, d, 2xCH$_3$-(*Ar*)C-CH-CH-C, $^3J_{H,H}$ = 5.6 Hz), 4.95 (2H, d, 2xCH$_3$-(*Ar*)C-CH-CH-C, $^3J_{H,H}$ = 6.1 Hz), 4.93

(2H, d, 2xCH$_3$-(*Ar*)C-C*H*-CH-C, $^3J_{H,H}$ = 6.0 Hz), 4.87 (2H, s br, S-(*Ar*)C-C-CH$_2$-(*Tr*)N-N=N-C-C*H*$_2$-OH), 4.67 (2H, d, 2xCH$_3$-(*Ar*)C-*C*H-CH-C, $^3J_{H,H}$ = 5.8 Hz), 3.70 (2H, s, S-C*H*$_2$-(*Ar*)C-CH-CH-C-(CH$_3$)$_3$), 3.35 (2H, s, S-C*H*$_2$-(*Ar*)C-CH-CH-C-(CH$_3$)$_3$), 1.86 (2H, sept, 2x(*Ar*)C-CH-CH-C-C*H*(CH$_3$)$_2$, $^3J_{H,H}$ = 6.9 Hz), 1.60 (6H, s, 2xC*H*$_3$-(*Ar*)C-CH-CH-C), 1.34 (9H, s, S-CH$_2$-(*Ar*)C-CH-CH-C-C(C*H*$_3$)$_3$), 1.33 (9H, s, S-CH$_2$-(*Ar*)C-CH-CH-C-C(C*H*$_3$)$_3$), 0.91 (6H, d, (*Ar*)C-CH-CH-C-CH(C*H*$_3$)$_2$, $^3J_{H,H}$ = 6.9 Hz), 0.88 (6H, d, (*Ar*)C-CH-CH-C-CH(C*H*$_3$)$_2$, $^3J_{H,H}$ = 6.9 Hz).

^{13}C-NMR (CDCl$_3$) δ_C, ppm: 151.8, 151.5 (2C, 2xS-CH$_2$-(*Ar*)C-CH-CH-*C*-C(CH$_3$)$_3$), 149.0 (1C, (*Tr*)N-N=N-*C*-CH), 139.3 (1C, S-(*Ar*)*C*-C-CH-CH-CH-CH), 138.3 (1C, S-(*Ar*)C-*C*-CH-CH-CH-CH), 136.8, 136.7 (2C, 2xS-CH$_2$-(*Ar*)*C*-CH-CH-C-C(CH$_3$)$_3$), 134.0 (1C, S-(*Ar*)C-*C*H-CH-CH-CH), 131.1 (1C, S-(*Ar*)C-CH-CH-CH-*C*H), 129.9 (2C, 2xS-CH$_2$-(*Ar*)C-CH-*C*H-C-C(CH$_3$)$_3$), 129.2 (2C, S-(*Ar*)C-CH-*C*H-CH-CH, S-(*Ar*)C-CH-CH-*C*H-CH), 129.0 (2C, 2xS-CH$_2$-(*Ar*)C-CH-*C*H-C-C(CH$_3$)$_3$), 125.7, 125.5 (4C, 2xS-CH$_2$-(*Ar*)C-*C*H-CH-C-C(CH$_3$)$_3$), 125.2 (1C, (*Tr*)N-N=N-C-*C*H), 106.7 (2C, 2xCH$_3$-(*Ar*)C-CH-CH-*C*), 101.4 (2C, 2xCH$_3$-(*Ar*)*C*-CH-CH-C), 83.6 (2C, 2xCH$_3$-(*Ar*)C-CH-*C*H-C), 83.4 (4C, 2xCH$_3$-(*Ar*)C-CH-*C*H-C, 2xCH$_3$-(*Ar*)C-*C*H-CH-C), 82.8 (2C, 2xCH$_3$-(*Ar*)C-*C*H-CH-C), 56.7 (1C, S-(*Ar*)C-C-CH$_2$-(*Tr*)N-N=N-C-*C*H$_2$-OH), 52.9 (1C, S-(*Ar*)C-C-*C*H$_2$-(*Tr*)N-N=N-C), 40.3 (1C, S-*C*H$_2$-(*Ar*)C-CH-CH-C-C(CH$_3$)$_3$), 39.6 (1C, S-*C*H$_2$-(*Ar*)C-CH-CH-C-C(CH$_3$)$_3$), 34.9 (2C, 2xS-CH$_2$-(*Ar*)C-CH-CH-C-*C*(CH$_3$)$_3$), 31.6, 31.5 (6C, 2xS-CH$_2$-(*Ar*)C-CH-CH-C-C(*C*H$_3$)$_3$), 30.9 (2C, 2x(*Ar*)CH-CH-C-*C*H(CH$_3$)$_2$), 23.2 (2C, (*Ar*)CH-CH-C-CH(*C*H$_3$)$_2$), 22.6 (2C, (*Ar*)CH-CH-C-CH(*C*H$_3$)$_2$), 18.1 (2C, 2x*C*H$_3$-(*Ar*)C-CH-CH).

R_f (CH$_2$Cl$_2$/CH$_3$OH 10:1) = 0.221.

ESI-MS(+): *m/z* found 1050.2614 [M-Cl]$^+$, calcd. for $C_{52}H_{68}N_3ORu_2S_3^+$ 1050.2606.

Elemental analysis (%): calcd. for $C_{52}H_{68}N_3ORu_2S_3Cl\cdot 2.25CH_3OH$ C 56.32, H 6.71, N 3.63; found C 56.36, H 6.85, N 3.60.

Synthesis of [(η^6-*p*-MeC$_6$H$_4$Pr^i)$_2$Ru$_2$(μ_2-SCH$_2$C$_6$H$_4$-*p*-Bu^t)$_2$(μ_2-SC$_6$H$_4$-*p*-R) (R = 4-phenyl-1*H*-1,2,3-triazole) (37)

To a solution of **10** (0.246 g, 0.235 mmol, 1 equiv.) and ethynylbenzene (0.03 mL, 0.282 mmol, 1.2 equiv.) in dry DMF (10 mL), were added successively $CuSO_4\cdot 5H_2O$ (0.059 g, 0.235 mmol, 1 equiv.) and sodium ascorbate (0.093 g, 0.470 mmol, 2 equiv.). The reaction mixture was stirred at r.t. under inert atmosphere (N$_2$) for further 24 h and the reaction evolution was verified by TLC. The reaction mixture was filtered and the filtrate was diluted with EtOAc (100 mL), washed with H$_2$O (2×100 mL), with brine (100 mL), dried over anhydrous Na$_2$SO$_4$, filtered and concentrated to dryness under reduced pressure. Purification by column chromatography (CH$_2$Cl$_2$/CH$_3$OH 10:1 (v/v)) afforded **37** as an orange solid (0.193 g, 0.186 mmol, yield 79%).

^{1}H-NMR (CDCl$_3$) δ_H, ppm: 9.21 (1H, s, (*Tr*)N-N=N-C-C*H*), 8.16 (2H, d, 2xS-(*Ar*)C-CH-C*H*-C-(*Tr*)N, $^3J_{H,H}$ = 8.7 Hz), 8.08 (2H, d, 2x(*Tr*)N-N=N-C-(*Ar*)C-C*H*-CH-CH, $^3J_{H,H}$ = 7.1 Hz), 8.01 (2H, d, 2xS-(*Ar*)C-C*H*-CH-C-(*Tr*)N, $^3J_{H,H}$ = 8.6 Hz), 7.39-7.51 (10H, m, 4xS-CH$_2$-(*Ar*)C-C*H*-CH-C-C(CH$_3$)$_3$, 4xS-CH$_2$-(*Ar*)C-CH-C*H*-C-C(CH$_3$)$_3$, 2x(*Tr*)N-N=N-C-(*Ar*)C-CH-C*H*-CH, $^3J_{H,H}$ = 8.6 Hz), 7.33 (1H, m, (*Tr*)N-N=N-C-(*Ar*)C-CH-CH-C*H*, $^3J_{H,H}$ = 7.4 Hz), 5.13 (2H, d, 2xCH$_3$-(*Ar*)C-CH-C*H*-C, $^3J_{H,H}$ = 5.7 Hz), 5.03 (2H, d, 2xCH$_3$-(*Ar*)C-CH-C*H*-C, $^3J_{H,H}$ = 5.8 Hz), 4.96 (2H, d, 2xCH$_3$-(*Ar*)C-C*H*-CH-C, $^3J_{H,H}$ = 5.8 Hz), 4.65 (2H, d, 2xCH$_3$-(*Ar*)C-C*H*-CH-C, $^3J_{H,H}$ = 5.9 Hz), 3.61 (2H, s, S-C*H*$_2$-(*Ar*)C-CH-CH-C-C(CH$_3$)$_3$), 3.41 (2H, s, S-C*H*$_2$-(*Ar*)C-CH-CH-C-C(CH$_3$)$_3$), 1.99 (2H, sept, 2x(*Ar*)C-CH-CH-C-C*H*(CH$_3$)$_2$, $^3J_{H,H}$ = 6.9 Hz), 1.75 (6H, s, 2xC*H*$_3$-(*Ar*)C-CH-CH-C), 1.37 (9H, s, S-CH$_2$-(*Ar*)C-CH-CH-C-C(C*H*$_3$)$_3$), 1.33 (9H, s, S-CH$_2$-(*Ar*)C-CH-CH-C-C(C*H*$_3$)$_3$), 0.95 (6H, d, (*Ar*)C-CH-CH-C-CH(C*H*$_3$)$_2$, $^3J_{H,H}$ = 6.9 Hz), 0.91 (6H, d, (*Ar*)C-CH-CH-C-CH(C*H*$_3$)$_2$, $^3J_{H,H}$ = 6.9 Hz).

^{13}C-NMR (CDCl$_3$) δ_C, ppm: 152.0, 151.9 (2C, 2xS-CH$_2$-(*Ar*)C-CH-CH-*C*-C(CH$_3$)$_3$), 148.8 (1C, (*Tr*)N-N=N-*C*-CH), 138.0 (1C, S-(*Ar*)C-CH-CH-*C*-(*Tr*)N), 137.2 (1C, S-(*Ar*)*C*-CH-CH-C-(*Tr*)N), 136.73,

136.65 (2C, 2xS-CH$_2$-(*Ar*)$\underline{C}$-CH-CH-C-C(CH$_3$)$_3$), 134.3 (2C, 2xS-(*Ar*)C-$\underline{C}$H-CH-C-(*Tr*)N), 130.5 (1C, (*Tr*)N-N=N-C-(*Ar*)$\underline{C}$-CH-CH-CH), 129.4, 129.2 (4C, 4xS-CH$_2$-(*Ar*)C-$\underline{C}$H-CH-C-C(CH$_3$)$_3$), 129.0 (2C, 2x(*Tr*)N-N=N-C-(*Ar*)C-CH-$\underline{C}$H-CH), 128.3 (1C, (*Tr*)N-N=N-C-(*Ar*)C-CH-CH-$\underline{C}$H), 126.2 (2C, 2x(*Tr*)N-N=N-C-(*Ar*)C-$\underline{C}$H-CH-CH), 125.7, 125.6 (4C, 4xS-CH$_2$-(*Ar*)C-CH-$\underline{C}$H-C-C(CH$_3$)$_3$), 120.7 (2C, 2xS-(*Ar*)C-CH-$\underline{C}$H-C-(*Tr*)N), 119.2 (1C, (*Tr*)N-N=N-C-$\underline{C}$H), 107.5 (2C, 2xCH$_3$-(*Ar*)C-CH-CH-$\underline{C}$), 100.6 (2C, 2xCH$_3$-(*Ar*)$\underline{C}$-CH-CH-C), 84.07 (2C, 2xCH$_3$-(*Ar*)C-CH-$\underline{C}$H-C), 84.04 (2C, 2xCH$_3$-(*Ar*)C-$\underline{C}$H-CH-C), 83.95 (2C, 2xCH$_3$-(*Ar*)C-CH-$\underline{C}$H-C), 82.5 (2C, 2xCH$_3$-(*Ar*)C-$\underline{C}$H-CH-C), 40.0 (1C, S-$\underline{C}$H$_2$-(*Ar*)C-CH-CH-C-C(CH$_3$)$_3$), 39.6 (1C, S-$\underline{C}$H$_2$-(*Ar*)C-CH-CH-C-C(CH$_3$)$_3$), 34.93, 34.88 (2C, 2xS-CH$_2$-(*Ar*)C-CH-CH-C-$\underline{C}$(CH$_3$)$_3$), 31.55, 31.53 (6C, 2xS-CH$_2$-(*Ar*)C-CH-CH-C-C($\underline{C}$H$_3$)$_3$), 31.1 (2C, 2x(*Ar*)CH-CH-C-$\underline{C}$H(CH$_3$)$_2$), 23.1 (2C, (*Ar*)CH-CH-C-CH($\underline{C}$H$_3$)$_2$), 22.9 (2C, (*Ar*)CH-CH-C-CH($\underline{C}$H$_3$)$_2$), 18.3 (2C, 2x$\underline{C}$H$_3$-(*Ar*)C-CH-CH).
R_f (CH$_2$Cl$_2$/CH$_3$OH 10:1) = 0.314.
ESI-MS(+): *m/z* found 1082.2664 [M-Cl]$^+$, calcd. for $C_{56}H_{68}N_3Ru_2S_3^+$ 1082.2657.
Elemental analysis (%): calcd. for $C_{56}H_{68}ClN_3Ru_2S_3\cdot 0.1CH_2Cl_2\cdot 2CH3OH$ C 58.67, H 6.46, N 3.53; found C 58.67, H 6.48, N 3.14.

Synthesis of [(η^6-*p*-MeC$_6$H$_4$Pr^i)$_2$Ru$_2$(μ_2-SCH$_2$C$_6$H$_4$-*p*-Bu^t)$_2$(μ_2-SC$_6$H$_4$-*p*-R) (R = (1*H*-1,2,3-triazol-4-yl)methanol) (38)

To a solution of **10** (0.300 g, 0.287 mmol, 1 equiv.) and propargyl alcohol (0.02 mL, 0.344 mmol, 1.2 equiv.) in dry DMF (10 mL) were added successively $CuSO_4\cdot 5H_2O$ (0.072 g, 0.287 mmol, 1 equiv.) and sodium ascorbate (0.114 g, 0.574 mmol, 2 equiv.). The reaction mixture was stirred at r.t. under inert atmosphere (N_2) for further 24 h and the reaction evolution was verified by TLC. The reaction mixture was concentrated to dryness under reduced pressure and purification by column chromatography (CH$_2$Cl$_2$/CH$_3$OH 10:1 (v/v)) afforded **38** as an orange solid (0.081 g, 0.076 mmol, yield 26%).
^{1}H-NMR (CDCl$_3$) δ_H, ppm: 8.98 (1H, s, (*Tr*)N-N=N-C-C$\underline{H}$), 7.88-7.95 (4H, m, 2xS-(*Ar*)C-C$\underline{H}$-CH-C-(*Tr*)N, 2xS-(*Ar*)C-CH-C$\underline{H}$-C-(*Tr*)N), 7.39-7.52 (8H, m, 4xS-CH$_2$-(*Ar*)C-C$\underline{H}$-CH-C-C(CH$_3$)$_3$, 4xS-CH$_2$-(*Ar*)C-CH-C$\underline{H}$-C-C(CH$_3$)$_3$, $^3J_{H,H}$ = 8.3 Hz), 5.10 (2H, d, 2xCH$_3$-(*Ar*)C-CH-C$\underline{H}$-C, $^3J_{H,H}$ = 5.5 Hz), 5.00 (2H, d, 2xCH$_3$-(*Ar*)C-CH-C$\underline{H}$-C, $^3J_{H,H}$ = 5.7 Hz), 4.96 (2H, s, S-(*Ar*)C-CH-CH-C-($\underline{Tr}$)N-N=N-C-C$\underline{H_2}$-OH), 4.89 (2H, d, 2xCH$_3$-(*Ar*)C-C$\underline{H}$-CH-C, $^3J_{H,H}$ = 5.5 Hz), 4.67 (2H, d, 2xCH$_3$-(*Ar*)C-C$\underline{H}$-CH-C, $^3J_{H,H}$ = 5.6 Hz), 3.62 (2H, s, S-C$\underline{H_2}$-(*Ar*)C-CH-CH-C-C(CH$_3$)$_3$), 3.42 (2H, s, S-C$\underline{H_2}$-(*Ar*)C-CH-CH-C-C(CH$_3$)$_3$), 1.98 (2H, sept, 2x(*Ar*)C-CH-CH-C-C$\underline{H}$(CH$_3$)$_2$, $^3J_{H,H}$ = 6.8 Hz), 1.75 (6H, s, 2xC$\underline{H_3}$-(*Ar*)C-CH-CH-C), 1.37 (9H, s, S-CH$_2$-(*Ar*)C-CH-CH-C-C(C$\underline{H_3}$)$_3$), 1.33 (9H, s, S-CH$_2$-(*Ar*)C-CH-CH-C-C(C$\underline{H_3}$)$_3$), 0.97 (6H, d, (*Ar*)C-CH-CH-C-CH(C$\underline{H_3}$)$_2$, $^3J_{H,H}$ = 6.8 Hz), 0.92 (6H, d, (*Ar*)C-CH-CH-C-CH(C$\underline{H_3}$)$_2$, $^3J_{H,H}$ = 6.8 Hz).
^{13}C-NMR (CDCl$_3$) δ_C, ppm: 152.1, 152.0 (2C, 2xS-CH$_2$-(*Ar*)C-CH-CH-$\underline{C}$-C(CH$_3$)$_3$), 150.8 (1C, (*Tr*)N-N=N-$\underline{C}$-CH), 138.0 (1C, S-(*Ar*)C-CH-CH-$\underline{C}$-(*Tr*)N), 137.5 (1C, S-(*Ar*)$\underline{C}$-CH-CH-C-(*Tr*)N), 136.7, 136.5 (2C, 2xS-CH$_2$-(*Ar*)$\underline{C}$-CH-CH-C-C(CH$_3$)$_3$), 134.0 (2C, S-(*Ar*)C-$\underline{C}$H-CH-C-(*Tr*)N), 129.4, 129.2 (4C, 4xS-CH$_2$-(*Ar*)C-$\underline{C}$H-CH-C-C(CH$_3$)$_3$), 125.8, 125.7 (4C, 4xS-CH$_2$-(*Ar*)C-CH-$\underline{C}$H-C-C(CH$_3$)$_3$), 122.0 (1C, (*Tr*)N-N=N-C-$\underline{C}$H), 120.7 (2C, 2xS-(*Ar*)C-CH-$\underline{C}$H-C-(*Tr*)N), 107.7 (2C, 2xCH$_3$-(*Ar*)C-CH-CH-$\underline{C}$), 100.5 (2C, 2xCH$_3$-(*Ar*)$\underline{C}$-CH-CH-C), 84.10 (2C, 2xCH$_3$-(*Ar*)C-CH-$\underline{C}$H-C), 84.05 (2C, 2xCH$_3$-(*Ar*)C-$\underline{C}$H-CH-C), 83.7 (2C, 2xCH$_3$-(*Ar*)C-CH-$\underline{C}$H-C), 82.5 (2C, 2xCH$_3$-(*Ar*)C-$\underline{C}$H-CH-C), 56.7 (1C, S-(*Ar*)C-CH-CH-C-($\underline{Tr}$)N-N=N-C-$\underline{C}$H$_2$-OH), 40.1 (1C, S-$\underline{C}$H$_2$-(*Ar*)C-CH-CH-C-C(CH$_3$)$_3$), 39.6 (1C, S-$\underline{C}$H$_2$-(*Ar*)C-CH-CH-C-C(CH$_3$)$_3$), 34.96, 34.92 (2C, 2xS-CH$_2$-(*Ar*)C-CH-CH-C-$\underline{C}$(CH$_3$)$_3$), 31.57, 31.54 (6C, 2xS-CH$_2$-(*Ar*)C-CH-CH-C-C($\underline{C}$H$_3$)$_3$), 31.2 (2C, 2x(*Ar*)CH-CH-C-$\underline{C}$H(CH$_3$)$_2$), 23.1 (2C, (*Ar*)CH-CH-C-CH($\underline{C}$H$_3$)$_2$), 22.9 (2C, (*Ar*)CH-CH-C-CH($\underline{C}$H$_3$)$_2$), 18.3 (2C, 2x$\underline{C}$H$_3$-(*Ar*)C-CH-CH).
R_f (CH$_2$Cl$_2$/CH$_3$OH 10:1) = 0.196.

ESI-MS(+): *m/z* found 1036.2462 [M-Cl]$^+$, calcd. for $C_{51}H_{66}N_3ORu_2S_3^+$ 1036.2450.
Elemental analysis (%): calcd. for $C_{51}H_{66}ClN_3ORu_2S_3 \cdot 0.4CH_2Cl_2$ C 55.88, H 6.09, N 3.80; found C 55.87, H 6.19, N 3.32.

Synthesis of [(η^6-*p*-MeC$_6$H$_4$Pr^i)$_2$Ru$_2$(μ_2-SCH$_2$C$_6$H$_4$-*p*-Bu^t)$_2$(μ_2-SC$_6$H$_4$-*p*-R) (R = 2-(1*H*-1,2,3-triazol-4-yl)pyridine) (39)
To a solution of **10** (0.306 g, 0.293 mmol, 1 equiv.) and 2-ethynylpyridine (0.04 mL, 0.352 mmol, 1.2 equiv.) in dry DMF (10 mL) were added successively $CuSO_4 \cdot 5H_2O$ (0.073 g, 0.293 mmol, 1 equiv.) and sodium ascorbate (0.116 g, 0.586 mmol, 2 equiv.). The reaction mixture was stirred at r.t. under inert atmosphere (N_2) for further 24 h and the reaction evolution was verified by TLC. The reaction mixture was diluted with EtOAc (100 mL) and washed with H_2O (2×100 mL); the unified aqueous phases were further washed with EtOAc (100 mL). The combined organic phases were washed with brine (100 mL), dried over anhydrous Na_2SO_4, filtered and concentrated to dryness under reduced pressure. Purification by column chromatography (CH_2Cl_2/CH_3OH 10:1 (v/v)) afforded **39** as an orange solid (0.030 g, 0.027 mmol, yield 9%).
^{1}H-NMR (DMSO-*d$_6$*) δ$_H$, ppm: 9.41 (1H, s, (*Tr*)N-N=N-C-C*H*), 8.68 (1H, m, (*Tr*)N-N=N-C-(*Py*)C-N-C*H*-CH, $^3J_{H,H}$ = 4.2 Hz), 8.14 (1H, d, (*Tr*)N-N=N-C-(*Py*)C-N-CH-CH-CH-C*H*, $^3J_{H,H}$ = 7.9 Hz), 7.97-8.03 (4H, m, 2xS-(*Ar*)C-CH-C*H*-C-(*Tr*)N, 2xS-(*Ar*)C-C*H*-CH-C-(*Tr*)N), 7.97 (1H, td, (*Tr*)N-N=N-C-(*Py*)C-N-CH-CH-C*H*, $^3J_{H,H}$ = 7.7 Hz, $^4J_{H,H}$ = 1.7 Hz), 7.41-7.53 (9H, m, 4xS-CH$_2$-(*Ar*)C-C*H*-CH-C-C(CH$_3$)$_3$, 4xS-CH$_2$-(*Ar*)C-CH-C*H*-C-C(CH$_3$)$_3$), (*Tr*)N-N=N-C-(*Py*)C-N-CH-C*H*), 5.44 (2H, d, 2xCH$_3$-(*Ar*)C-CH-C*H*-C, $^3J_{H,H}$ = 5.6 Hz), 5.30-5.34 (4H, m, 2xCH$_3$-(*Ar*)C-C*H*-CH-C, 2xCH$_3$-(*Ar*)C-CH-C*H*-C, $^3J_{H,H}$ = 6.5 Hz), 4.77 (2H, d, 2xCH$_3$-(*Ar*)C-C*H*-CH-C, $^3J_{H,H}$ = 5.7 Hz), 3.68 (2H, s, S-C*H$_2$*-(*Ar*)C-CH-CH-C-C(CH$_3$)$_3$), 3.49 (2H, s, S-C*H$_2$*-(*Ar*)C-CH-CH-C-C(CH$_3$)$_3$), 1.90 (2H, sept, 2x(*Ar*)C-CH-CH-C-C*H*(CH$_3$)$_2$, $^3J_{H,H}$ = 6.8 Hz), 1.80 (6H, s, 2xC*H$_3$*-(*Ar*)C-CH-CH-C), 1.34 (9H, s, S-CH$_2$-(*Ar*)C-CH-CH-C-C(C*H$_3$*)$_3$), 1.30 (9H, s, S-CH$_2$-(*Ar*)C-CH-CH-C-C(C*H$_3$*)$_3$), 0.84 (6H, d, (*Ar*)C-CH-CH-C-CH(C*H$_3$*)$_2$, $^3J_{H,H}$ = 6.8 Hz), 0.80 (6H, d, (*Ar*)C-CH-CH-C-CH(C*H$_3$*)$_2$, $^3J_{H,H}$ = 6.8 Hz).
^{13}C-NMR (DMSO-*d$_6$*) δ$_C$, ppm: 150.6, 150.4 (2C, 2xS-CH$_2$-(*Ar*)C-CH-CH-*C*-C(CH$_3$)$_3$), 149.7 (1C, (*Tr*)N-N=N-C-(*Py*)C-N-*C*H-CH), 149.4 (1C, (*Tr*)N-N=N-C-(*Py*)*C*-N-CH-CH), 148.4 (1C, (*Tr*)N-N=N-*C*-CH), 138.8 (1C, S-(*Ar*)C-CH-CH-*C*-(*Tr*)N), 137.4 (1C, (*Tr*)N-N=N-C-(*Py*)C-N-CH-CH-*C*H), 136.9, 136.8 (2C, 2xS-CH$_2$-(*Ar*)*C*-CH-CH-C-C(CH$_3$)$_3$), 135.8 (1C, S-(*Ar*)*C*-CH-CH-C-(*Tr*)N), 134.0 (2C, 2xS-(*Ar*)C-*C*H-CH-C-(*Tr*)N), 129.3, 129.0 (4C, 4xS-CH$_2$-(*Ar*)C-*C*H-CH-C-C(CH$_3$)$_3$), 125.3, 125.1 (4C, 4xS-CH$_2$-(*Ar*)C-CH-*C*H-C-C(CH$_3$)$_3$), 123.5 (1C, (*Tr*)N-N=N-C-(*Py*)C-N-CH-*C*H), 121.1 (1C, (*Tr*)N-N=N-C-*C*H), 120.0 (2C, 2xS-(*Ar*)C-CH-*C*H-C-(*Tr*)N), 119.9 (1C, (*Tr*)N-N=N-C-(*Py*)C-N-CH-CH-CH-*C*H), 105.7 (2C, 2xCH$_3$-(*Ar*)C-CH-CH-*C*), 101.4 (2C, 2xCH$_3$-(*Ar*)*C*-CH-CH-C), 84.8 (2C, 2xCH$_3$-(*Ar*)C-CH-*C*H-C), 83.0 (2C, 2xCH$_3$-(*Ar*)C-*C*H-CH-C, 82.7 (2C, 2xCH$_3$-(*Ar*)C-CH-*C*H-C), 82.3 (2C, 2xCH$_3$-(*Ar*)C-*C*H-CH-C), 39.7 (1C, S-*C*H$_2$-(*Ar*)C-CH-CH-C-C(CH$_3$)$_3$), 39.5 (1C, S-*C*H$_2$-(*Ar*)C-CH-CH-C-C(CH$_3$)$_3$), 34.43, 34.38 (2C, 2xS-CH$_2$-(*Ar*)C-CH-CH-C-*C*(CH$_3$)$_3$), 31.2 (6C, 2xS-CH$_2$-(*Ar*)C-CH-CH-C-C(*C*H$_3$)$_3$), 29.0 (2C, 2x(*Ar*)CH-CH-C-*C*H(CH$_3$)$_2$), 22.9 (2C, (*Ar*)CH-CH-C-CH(*C*H$_3$)$_2$), 21.9 (2C, (*Ar*)CH-CH-C-CH(*C*H$_3$)$_2$), 17.6 (2C, 2x*C*H$_3$-(*Ar*)C-CH-CH).
R*$_f$* (CH_2Cl_2/CH_3OH 10:1) = 0.240.
ESI-MS(+): *m/z* found 1083.2722 [M-Cl]$^+$, calcd. for $C_{55}H_{67}N_4Ru_2S_3^+$ 1083.2609.
Elemental analysis (%): calcd. for $C_{55}H_{67}ClN_4Ru_2S_3 \cdot CH_3OH$ C 58.49, H 6.22, N 4.87; found C 58.46, H 6.47, N 4.38.

8. Stability in DMSO-d_6

The conjugates are well soluble in DMSO, solvent used to prepare standard solutions for biological assays.

To assess their stability, compounds **4**, **24**, **28**, **33** and **39** were dissolved in DMSO-d_6, and two ^{1}H NMR spectra were recorded at 25°C 5 min and at least 100 days after sample preparation (Figures S1 and S2). Between the two experiments, the samples were stored at 0°C. For all compounds, there are no significant changes between the spectrum recorded after 5 min and the spectrum recorded after at least 100 days at 0°C, which indicates a very good stability, and makes this type of derivatives suitable for further biological tests.

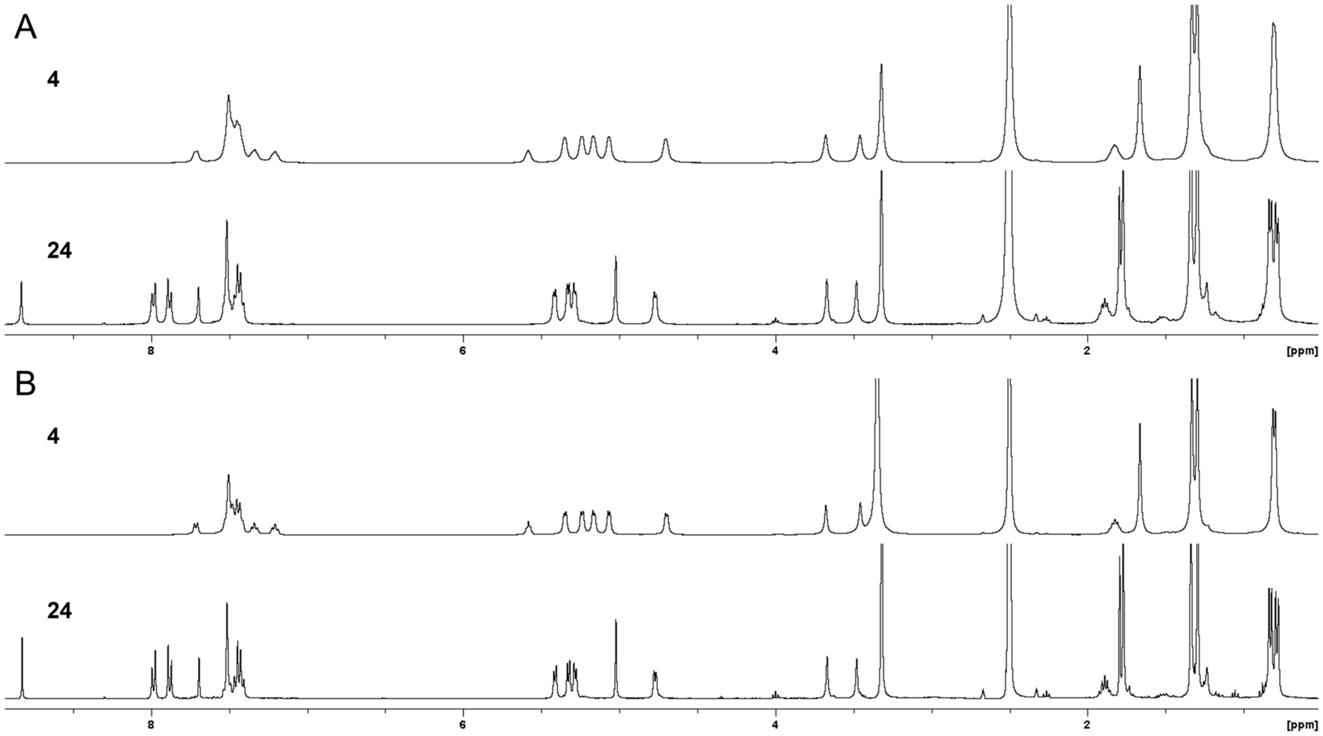

Figure S1. ^{1}H NMR spectra of **4** and **24** recorded in DMSO-d_6 at 25°C; (A) recorded 5 min after sample preparation, and (B) sample after > 100 days storage at 0-5°C in the dark.

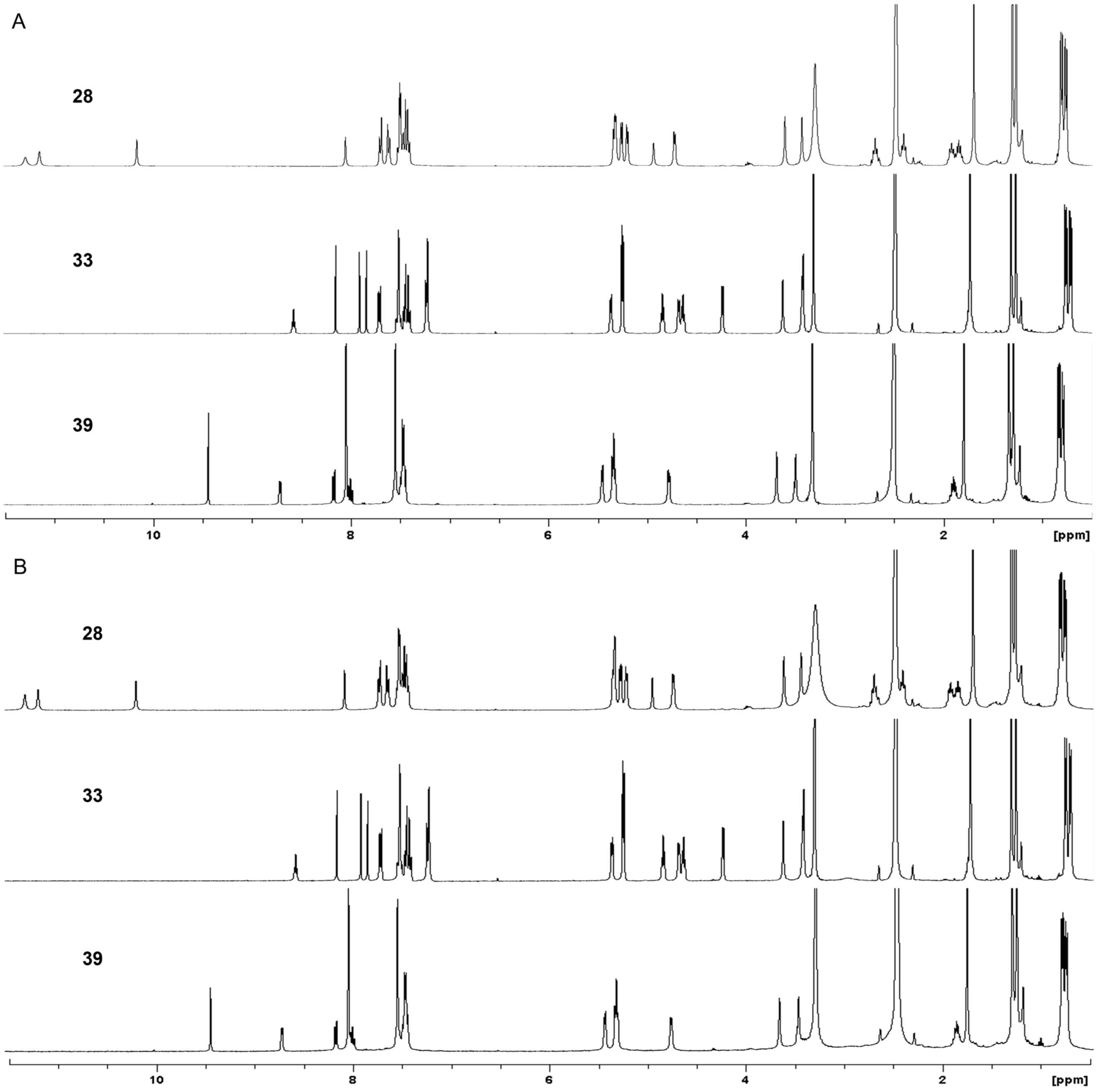

Figure S2. ^{1}H NMR spectra of **28**, **33** and **39** recorded in DMSO-d_6 at 25°C; (A) recorded 5 min after sample preparation, and (B) sample after > 100 days storage at 0-5°C in the dark.

The ability of nucleobase-diruthenium conjugates **23**, **24**, **25** and **26** to form pairs with complementary nucleobases *via* hydrogen bond interactions was investigated using ^{1}H NMR experiments. The spectra were recorded on a Bruker Avance II 400 (400.13 MHz) spectrometer at 298 K. The diruthenium conjugate (ca. 5 mg) was solubilized in 0.5 mL of DMSO-d_6. A sample of the complementary nucleobase in DMSO-d_6 was prepared in parallel. ^{1}H NMR spectra were recorded for both starting solutions. The solution of the complementary nucleobase was added in small aliquots to the solution of diruthenium conjugate to achieve the molar ratios 1:1 and ^{1}H NMR spectra were recorded for each mixture at 5 min after sample preparation.

The most dramatic changes were observed at the addition of guanine to cytosine conjugate **25** (relative molar proportion 1:1, Figure S3) indicating possible interaction *via* three H-bonds. In this case the signals corresponding to guanine at 6.04, 6.25 10.45 and 10.63 ppm were slightly broadened and correspondingly downfield shifted to 6.08, 6.30, 10.52 and 10.69 ppm (Figure S3). Also, upon addition

of guanine, the cytosine signals in conjugate **25** at 7.02 and 7.15 ppm slightly shifted to 7.03 and 7.14 ppm, respectively (Figure S3).

The changes were less striking for adenine-uracil and adenine-thymine interactions (interactions possible *via* two H-bonds). Upon addition of adenine to uracil conjugate **23** (relative molar proportion 1:1, Figure S4), the adenine signal at 7.09 ppm was slightly upfield shifted to 7.07 ppm, while the signal at 12.79 ppm is downfield shifted to 12.82 ppm (Figure S4). In parallel, the uracil signal at 11.35 ppm in conjugate **23** became broader. A similar pattern was observed in case of thymine conjugate **24** and adenine (Figure S5).

At the addition of uracil to adenine conjugate **26** only the uracil signal at 10.80 ppm was downfield shifted to 10.82 ppm (Figure S6).

The addition of thymine to adenine conjugate **26** (relative molar proportion 1:1), led only to slight shifts of the thymine signals from 10.57 and 10.98 ppm to 10.59 and 10.99 ppm, while the adenine signal in conjugate **26** was downfield shifted to from 7.51 to 7.53 ppm (Figure S7).

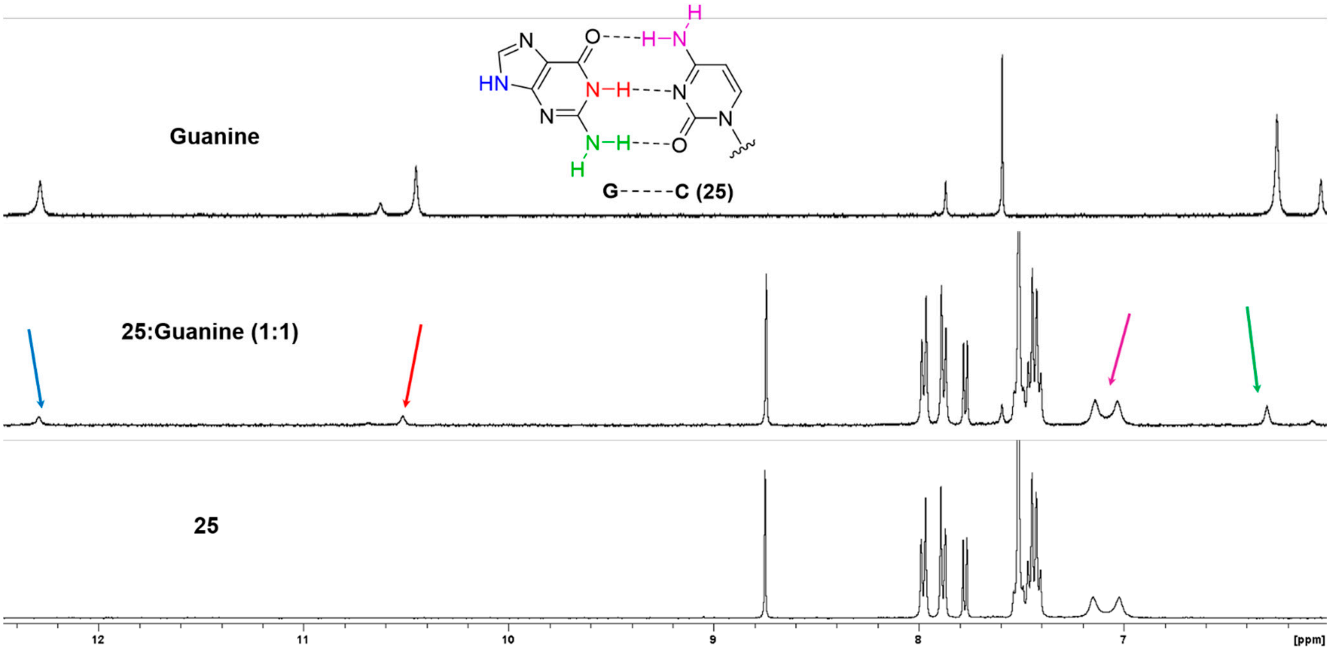

Figure S3. Study of H-bond interactions with complementary nucleobases by ^{1}H NMR at r.t. for cytosine conjugate **25** and guanine.

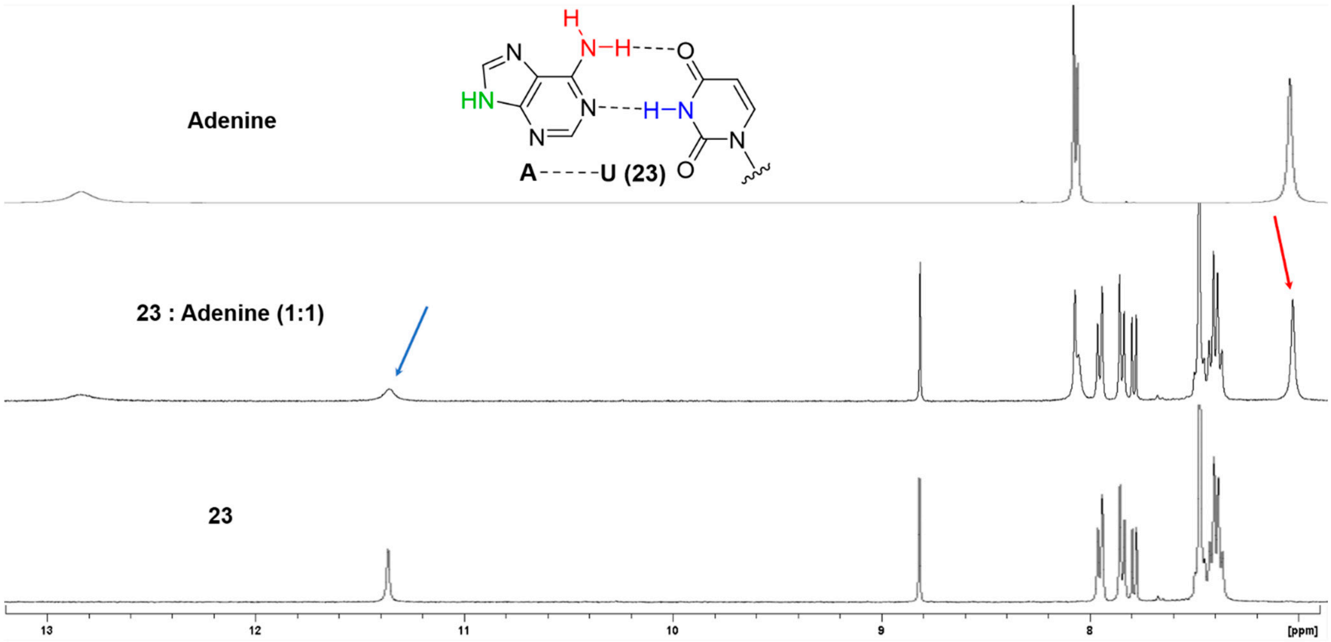

Figure S4. Study of H-bond interactions with complementary nucleobases by ^{1}H NMR for uracil conjugate **23** and adenine.

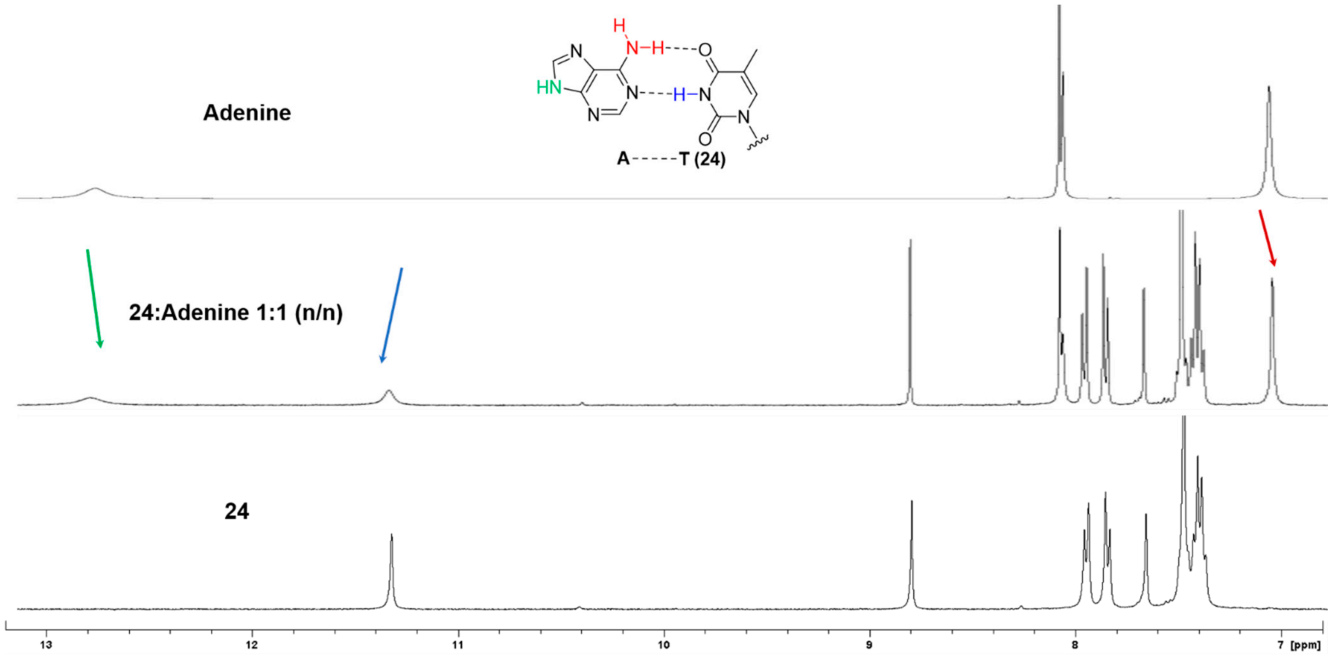

Figure S5. Study of H-bond interactions with complementary nucleobases by ^{1}H NMR at r.t. for thymine conjugate **24** and adenine.

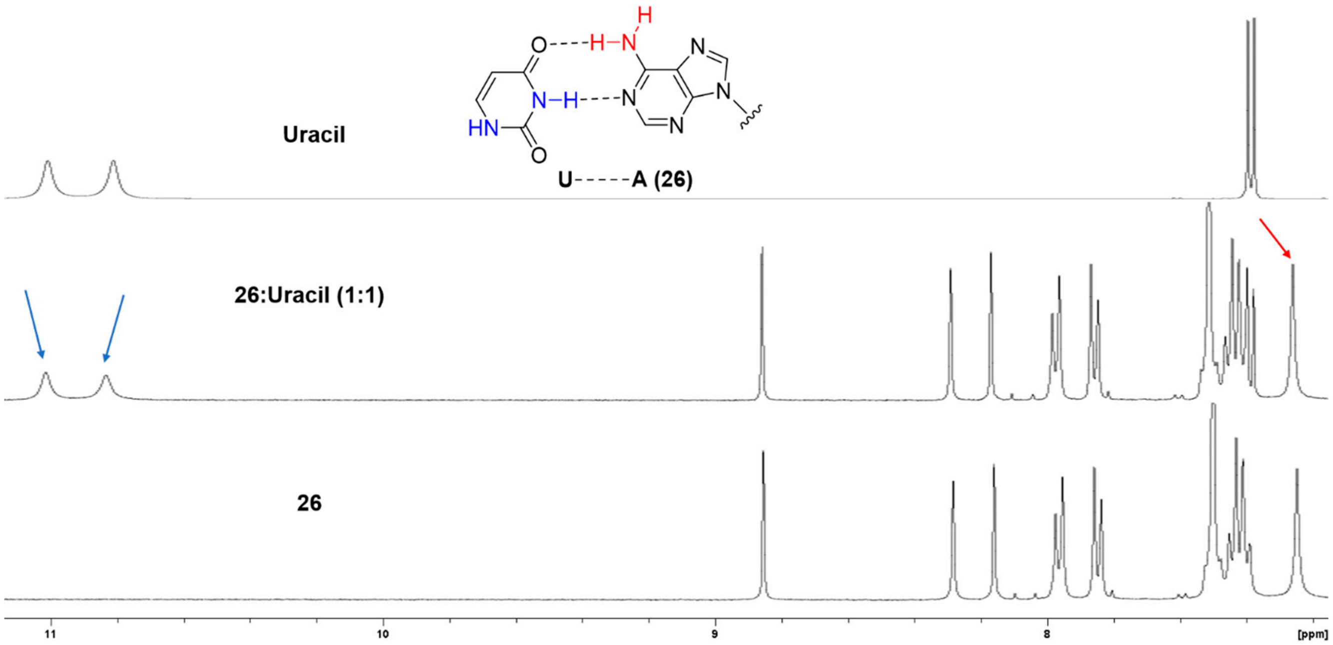

Figure S6. Study of H-bond interactions with complementary nucleobases by ^{1}H NMR for adenine conjugate **26** and uracil.

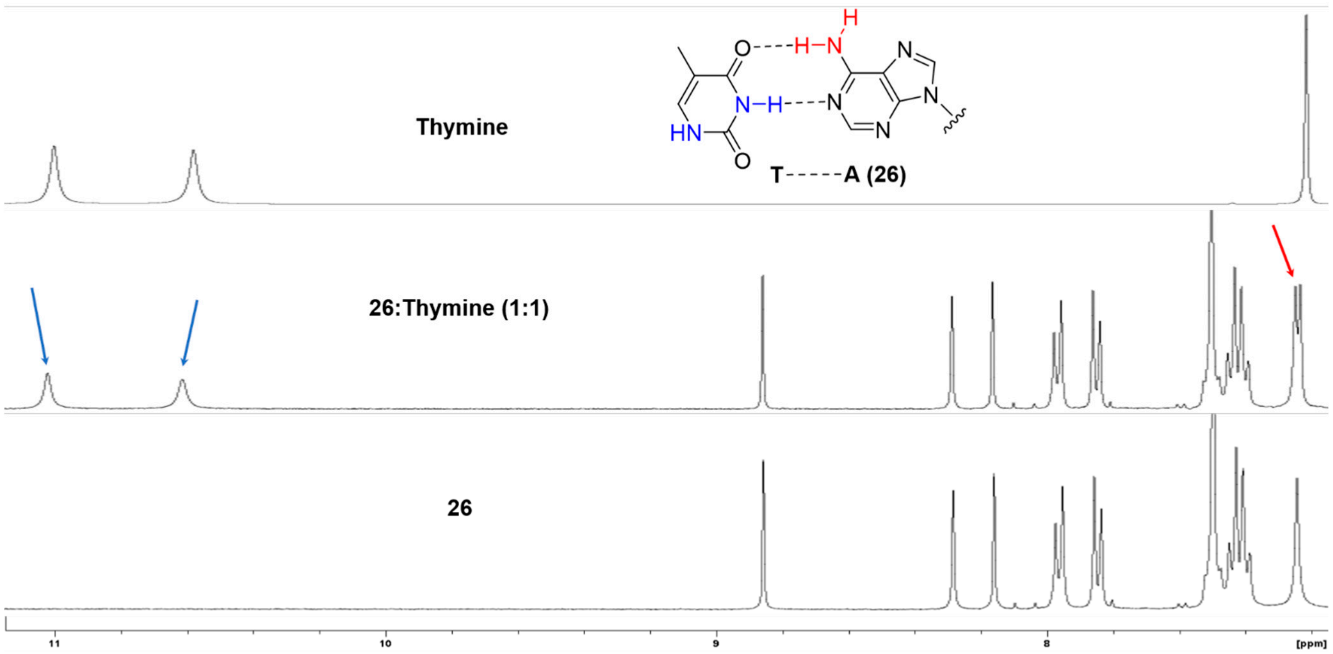

Figure S7. Study of H-bond interactions with complementary nucleobases by ^{1}H NMR for adenine conjugate **26** and thymine.
